# Supplementary material for: Immunogenicity and safety of mRNA-based seasonal influenza vaccines encoding hemagglutinin and neuraminidase
Source: Nat Commun. 2025 Jul 1;16:5933. doi: 10.1038/s41467-025-60938-4 (PMC12216828; doi:10.1038/s41467-025-60938-4)
Supplement: Supplementary file 1 — Supplementary Information [file 41467_2025_60938_MOESM1_ESM.pdf]

SUPPLEMENTARY APPENDIX

Immunogenicity and safety of mRNA-based seasonal influenza vaccines encoding hemagglutinin and neuraminidase

Authors: Amanda K. Rudman Spergel, Ivan T. Lee, Kindra Koslovsky, Kristi Schaefers, Andrei Avanesov, Douglas K. Logan, John Hemmersmeier, David Ensz, Daniel Stadlbauer, Bo Hu, Alicia Pucci, Jignesh Vakil, Robert Paris, Jintanat Ananworanich, Raffael Nachbagauer

Supplementary Methods 3

Analysis populations 3

Hemagglutination inhibition (HAI) assay 3

Neuraminidase inhibition (NAI) assay 4

Supplementary Table 1. Baseline demographics and characteristics (full analysis set). 5

Supplementary Table 2. Unsolicited adverse events after vaccination (safety population). ... 9

Supplementary Table 3. Summary of hemagglutinin antibody responses by hemagglutination inhibition assay through day 181 (per-protocol population). 13

Supplementary Table 4. Summary of neuraminidase antibody responses by neuraminidase inhibition assay through day 181 (per-protocol population). 24

Supplementary Table 5. Approximate HA and NA content in each vaccine. 46

Supplementary Fig. 1. Study arms. 47

Supplementary References 48

Clinical Study Protocol 49

Statistical Analysis Plan 150

Supplementary Methods

Analysis populations

The full analysis set consisted of all randomly assigned participants who received the investigational product. The per-protocol population consisted of all participants in the full analysis set who complied with the injection schedule and the timings of immunogenicity blood sampling to have a baseline and at least one post-injection assessment, did not have influenza infection at baseline through day 29, and had no major protocol deviations that impacted the immune response. The safety population consisted of all randomly assigned participants who received the investigational product. The solicited safety population consisted of all participants in the safety population who contributed any solicited adverse reaction data.

Hemagglutination inhibition (HAI) assay

Sera from participants were tested by HAI assay using standard methods.[1] In brief, receptor-destroying enzyme was used to remove non-specific inhibitors of hemagglutination. Eleven two-fold serial dilutions of treated serum samples were prepared in duplicate 96-well plates. Working dilutions of influenza virus equal to four HA units per well (per World Health Organization recommendations) were added to the serum dilutions and incubated for 60 minutes at room temperature. Guinea pig red blood cell suspension (0.75%) was added to the serum-virus mixture and incubated for 60 minutes at room temperature. Following incubation, plates were read and the geometric mean titer was calculated from duplicate plate readings. The HAI titer was defined as the last dilution at which agglutination of red blood cells was inhibited.

Neuraminidase inhibition (NAI) assay

The NAI enzyme-linked lectin assay (ELLA) was performed using recombinant neuraminidase (NAse) from influenza. Prior to the ELLA testing, the NAse working concentration (70% effective concentration: EC_{70}) was determined using a dose–response of the NAse on fetuin-coated plates and by the selection of the NAse concentration that results in 70% NA activities (i.e. 70% effective concentration).

Heat-inactivated serum samples were then diluted two-fold and were added to the fetuin-coated microplates plates. NAse was added to the serum dilutions and the serum-NAse mixture was incubated overnight in a humidified chamber. Each microplate included a maximum NAse activity (NAse max) and a blank (assay buffer). After overnight incubation, microplates were washed and horseradish peroxidase-labelled lectin from *Arachis hypogaea* (peanut) (PNA-HRP) was added to all wells. After 2 h PNA-HRP incubation, microplates were washed and 3,3’,5,5’-tetramethylbenzidine (TMB) was added to all wells for 30 min. The reaction was stopped by addition of 2N sulfuric acid, and the optical density was read at a wavelength of 450 nm (OD450) using a plate reader and the Softmax Pro.

To determine the titer, the average of the blank OD wells, neuraminidase maximal activity OD, as well as samples OD tested were calculated. Blank OD average was subtracted from neuraminidase maximal activity OD and samples OD reading. The percentage of inhibition was then calculated by the following formula:

$$\% \text{ inhibition} = 100\left(1 - \frac{Sample\ OD_{450}}{NAse_{max}}\right)$$

The titer (IC_{50}) was defined as the highest serum dilution inhibiting at least 50% of the neuraminidase activity.

Supplementary Table 1. Baseline demographics and characteristics (full analysis set)

	Recombinant vaccine *n* = 71	mRNA-1010 50 µg *n* = 71	mRNA-1020 50 µg *n* = 71	mRNA-1020 100 µg *n* = 67	mRNA-1020 150 µg *n* = 70	mRNA-1030 25 µg *n* = 71	mRNA-1030 50 µg *n* = 72	mRNA-1030 100 µg *n* = 72	Overall *N = 565*
**Age, years**									
Mean ± SD	46.2 ± 14.58	46.0 ± 15.03	47.9 ± 16.60	47.5 ± 15.00	49.2 ± 15.98	47.1 ± 14.40	46.9 ± 14.11	47.3 ± 17.13	47.3 ± 15.33
Median (range)	49.0 (21-75)	51.0 (22-72)	49.0 (19-75)	49.0 (19-73)	50.0 (20-75)	50.0 (19-75)	49.5 (20-74)	49.5 (18-75)	49.0 (18-75)
**Age group, years — no.**									
18-49	36	35	36	34	35	35	36	36	283
50-75	35	36	36	35	36	33	36	35	282

**Sex — no. (%)**									
Male	34 (47.9)	38 (53.5)	29 (40.8)	28 (41.8)	31 (44.3)	27 (38.0)	34 (47.2)	33 (45.8)	254 (45.0)
Female	37 (52.1)	33 (46.5)	42 (59.2)	39 (58.2)	39 (55.7)	44 (62.0)	38 (52.8)	39 (54.2)	311 (55.0)
**Race — no. (%)**									
White	59 (83.1)	52 (73.2)	64 (90.1)	55 (82.1)	59 (84.3)	51 (71.8)	54 (75.0)	61 (84.7)	455 (80.5)
Black/African American	6 (8.5)	11 (15.5)	6 (8.5)	10 (14.9)	5 (7.1)	15 (21.1)	13 (18.1)	7 (9.7)	73 (12.9)
Asian	3 (4.2)	3 (4.2)	0	1 (1.5)	3 (4.3)	3 (4.2)	4 (5.6)	4 (5.6)	21 (3.7)
American Indian or Alaska Native	2 (2.8)	3 (4.2)	0	1 (1.5)	1 (1.4)	1 (1.4)	0	0	8 (1.4)
Native Hawaiian or Other Pacific Islander	0	1 (1.4)	0	0	0	0	0	0	1 (0.2)
Other	1 (1.4)	0	0	0	1 (1.4)	0	0	0	2 (0.4)
Not reported	0	0	0	0	0	0	0	0	0 (0)
Multiple	0	1 (1.4)	1 (1.4)	0	1 (1.4)	1 (1.4)	1 (1.4)	0	5 (0.9)

Unknown	0	0	0	0	0	0	0	0	0 (0)
**Ethnicity — no. (%)**									
Hispanic or Latino	7 (9.9)	5 (7.0)	7 (9.9)	7 (10.4)	6 (8.6)	6 (8.5)	6 (8.3)	4 (5.6)	48 (8.5)
Not Hispanic or Latino	63 (88.7)	66 (93.0)	64 (90.1)	60 (89.6)	64 (91.4)	65 (91.5)	65 (90.3)	67 (93.1)	514 (91)
Not reported	1 (1.4)	0	0	0	0	0	0	1 (1.4)	2 (0.4)
Unknown	0	0	0	0	0	0	1 (1.4)	0	1 (0.2)
**Weight, kg**									
Mean ± SD	81.7 ± 14.9	79.3 ± 15.8	77.0 ± 14.7	83.1 ± 17.6	82.3 ± 15.5	78.8 ± 17.1	83.6 ± 18.3	79.6 ± 17.2	80.7 ± 16.5
**Height, cm**									
Mean ± SD	171.3 ± 10.0	170.1 ± 9.4	169.1 ± 9.2	170.6 ± 11.3	169.4 ± 10.7	169.5 ± 9.9	172.5 ± 9.2	169.9 ± 9.6	170.3 ± 9.9
**BMI, kg/m^2**									

Mean ± SD	27.8 ± 4.1	27.2 ± 3.7	27.0 ± 4.8	28.4 ± 4.3	28.6 ± 3.8	27.3 ± 4.6	27.9 ± 4.8	27.4 ± 4.6	27.7 ± 4.4
**Received seasonal influenza vaccine since September 2021 — no. (%)**									
Yes	17 (23.9)	14 (19.7)	19 (26.8)	14 (20.9)	19 (27.1)	13 (18.3)	16 (22.2)	17 (23.6)	129 (22.8)
No	54 (76.1)	57 (80.3)	52 (73.2)	53 (79.1)	51 (72.9)	58 (81.7)	56 (77.8)	55 (76.4)	436 (77.2)

The full analysis set consisted of all randomly assigned participants who received the investigational product.

BMI = body mass index.

Supplementary Table 2. Unsolicited adverse events after vaccination (safety population)

			mRNA-1020				mRNA-1030			
	**Recombinant vaccine** *n* **= 71**	**mRNA-1010 50 µg** *n* **= 71**	**50 µg** *n* **= 71**	**100 µg** *n* **= 67**	**150 µg** *n* **= 70**	**Any dose** ***n* = 208**	**25 µg** *n* **= 71**	**50 µg** *n* **= 72**	**100 µg** *n* **= 72**	**Any dose** ***n* = 215**
**Any unsolicited AE(s) — no. (%)[a]**										
**Up to 28 days**	10 (14.1)	17 (23.9)	15 (21.1)	15 (22.4)	21 (30.0)	51 (24.5)	6 (8.5)	11 (15.3)	12 (16.7)	29 (13.5)
Severe[b]	0	0	0	1 (1.5)	2 (2.9)	3 (1.4)	0	0	0	0
**End of study (day 181)**										
Severe[b]	2 (2.8)	1 (1.4)	1 (1.4)	1 (1.5)	2 (2.9)	4 (1.9)	1 (1.4)	0	2 (2.8)	3 (1.4)
Serious[c]	2 (2.8)	1 (1.4)	1 (1.4)	0	4 (5.7)	5 (2.4)	1 (1.4)	1 (1.4)	2 (2.8)	4 (1.9)
Fatal	1 (1.4)	0	0	0	0	0	0	0	0	0
Of special interest[d]	1 (1.4)	1 (1.4)	1 (1.4)	1 (1.5)	0	2 (1.0)	1 (1.4)	1 (1.4)	0	2 (0.9)

Medically attended[e]	23 (32.4)	20 (28.2)	24 (33.8)	19 (28.4)	23 (32.9)[f]	66 (31.7)[f]	16 (22.5)	18 (25.0)	17 (23.6)	51 (23.7)
Leading to discontinuation of study participation	1 (1.4)	0	0	0	0	0	0	0	0	0
**Any unsolicited AE(s) related to study vaccination[g] — no. (%)[a]**										
**Up to 28 days**	1 (1.4)	8 (11.3)	4 (5.6)	3 (4.5)	8 (11.4)	15 (7.2)	1 (1.4)	3 (4.2)	7 (9.7)	11 (5.1)
Severe[b]	0	0	0	0	0	0	0	0	0	0
**End of study (day 181)**										
Severe[b]	0	0	0	0	0	0	0	0	0	0
Serious[c]	0	0	0	0	0	0	0	0	0	0
Fatal	0	0	0	0	0	0	0	0	0	0
Of special interest[d]	0	0	0	1 (1.5)	0	1 (0.5)	1 (1.4)	0	0	1 (0.5)
Medically attended[e]	0	0	1 (1.4)	1 (1.5)	1 (1.4)[f]	3 (1.4)[f]	2 (2.8)	0	0	2 (0.9)

Leading to discontinuation of study participation	0	0	0	0	0	0	0	0	0	0

AE = adverse event.

[a]no. (%) represents the number and percentage of participants.

[b]A severe AE was defined as any AE that prevented the participant's daily activity and required intensive therapeutic intervention.

[c]A serious AE was defined as any AE that was life-threatening or resulted in death, inpatient hospitalization or prolonged hospitalization, persistent or significant incapacity or substantial disruption of the ability to conduct normal life functions, congenital anomaly or birth defect, or a medically important event.

[d]AEs of special interest were anaphylaxis, myocarditis/pericarditis, thrombocytopenia ($<150\times10^9$ platelet count), and new onset or worsening of specific neurologic diseases (Guillain-Barré syndrome; acute disseminated encephalomyelitis; idiopathic peripheral facial nerve palsy [Bell's palsy]; seizures, including, but not limited to, febrile seizures and/or generalized seizures/convulsions).

[e]A medically attended AE was defined as any AE that led to an unscheduled visit to a healthcare practitioner.

[f]One medically attended AE of pyrexia reported in the mRNA-1020 150 µg group (classified as related to study vaccination) was a confirmed and documented data entry error by the trial site.

[g]The investigator assessed the causality of all AEs and determined them to be not related (not a reasonable possibility of a relationship to the vaccine) or related (a reasonable possibility of a relationship to the vaccine) to study vaccination.

Supplementary Table 3. Summary of hemagglutinin antibody responses by hemagglutination inhibition assay through day 181 (per-protocol population)

	Recombinant vaccine (*n* = 67)	mRNA-1010 50 µg (*n* = 63)	mRNA-1020			mRNA-1030		
			50 µg (*n* = 66)	100 µg (*n* = 64)	150 µg (*n* = 63)	25 µg (*n* = 67)	50 µg (*n* = 66)	100 µg (*n* = 67)
**A/H1N1: anti-H1 antibodies by HAI assay**								
**Baseline (day 1)**								
no.	65	61	63	60	61	67	63	66
GMT (95% CI)	73.00 (53.19, 100.19)	54.65 (41.48, 71.99)	55.25 (40.87, 74.69)	45.09 (34.19, 59.45)	77.74 (58.27, 103.72)	62.07 (45.19, 85.25)	56.82 (42.09, 76.70)	64.14 (47.69, 86.26)
**Day 8**								
no.	59	60	59	58	60	61	62	63
GMT (95% CI)	348.67 (236.32,	242.49 (181.00,	207.90 (151.25,	186.93 (136.89,	344.86 (256.99,	167.43 (122.81,	195.65 (141.71,	281.89 (212.63,

	514.44)	324.88)	285.77)	255.26)	462.78)	228.25)	270.11)	373.72)
GMFR (95% CI)	4.37 (2.79, 6.87)	4.32 (3.18, 5.89)	3.97 (2.89, 5.47)	4.16 (3.00, 5.78)	4.32 (3.11, 6.00)	2.78 (2.04, 3.79)	3.18 (2.24, 4.54)	4.65 (3.43, 6.30)
Participants with seroconversion — no. (%) (95% CI)	27 (47.4) (33.98, 61.03)	31 (53.4) (39.87, 66.66)	32 (57.1) (43.22, 70.29)	28 (51.9) (37.84, 65.66)	30 (50.8) (37.50, 64.11)	23 (37.7) (25.61, 51.04)	21 (35.6) (23.55, 49.13)	34 (54.8) (41.68, 67.52)
**Day 29**								
no.	65	61	63	60	61	67	63	66
GMT (95% CI)	724.69 (527.41, 995.78)	498.43 (392.33, 633.22)	472.81 (345.73, 646.61)	378.34 (267.06, 535.99)	527.55 (404.11, 688.70)	328.44 (243.71, 442.62)	387.92 (291.94, 515.47)	431.61 (325.80, 571.78)
GMFR (95% CI)	9.93 (6.60, 14.94)	9.12 (6.63, 12.55)	8.56 (6.03, 12.15)	8.39 (5.93, 11.88)	6.79 (4.86, 9.48)	5.29 (3.77, 7.42)	6.83 (4.96, 9.39)	6.73 (5.01, 9.04)

Participants with seroconversion — no. (%) (95% CI)	43 (66.2) (53.35, 77.43)	46 (75.4) (62.71, 85.54)	47 (74.6) (62.06, 84.73)	43 (71.7) (58.56, 82.55)	41 (67.2) (54.00, 78.69)	38 (56.7) (44.04, 68.78)	45 (71.4) (58.65, 82.11)	46 (69.7) (57.15, 80.41)
**Day 181**								
no.	59	61	60	60	61	64	63	62
GMT (95% CI)	203.10 (145.63, 283.25)	170.30 (129.94, 223.20)	171.47 (124.45, 236.24)	120.48 (84.40, 171.97)	164.66 (121.59, 222.99)	138.97 (101.24, 190.77)	141.74 (103.95, 193.27)	149.60 (113.99, 196.33)
GMFR (95% CI)	2.71 (1.88, 3.90)	3.07 (2.25, 4.19)	3.34 (2.30, 4.85)	2.63 (1.91, 3.63)	2.08 (1.55, 2.81)	2.24 (1.59, 3.16)	2.64 (1.88, 3.70)	2.36 (1.82, 3.06)
Participants with seroconversion — no. (%) (95% CI)	26 (45.6) (32.36, 59.34)	27 (45.0) (32.12, 58.39)	26 (45.6) (32.36, 59.34)	19 (33.3) (21.40, 47.06)	13 (21.7) (12.07, 34.20)	24 (37.5) (25.70, 50.49)	26 (43.3) (30.59, 56.76)	24 (39.3) (27.07, 52.69)
**A/H3N2: anti-H3 antibodies by HAI assay**								

**Baseline (day 1)**								
no.	65	61	63	60	61	67	63	66
GMT (95% CI)	50.83 (35.91, 71.96)	40.92 (29.78, 56.21)	40.42 (29.42, 55.55)	33.24 (23.49, 47.04)	43.07 (31.93, 58.09)	54.29 (39.89, 73.88)	38.90 (27.87, 54.29)	47.80 (34.60, 66.04)
**Day 8**								
n	59	60	59	58	60	61	62	63
GMT (95% CI)	193.05 (140.03, 266.14)	164.69 (123.53, 219.55)	121.39 (88.43, 166.65)	138.63 (97.30, 197.50)	181.63 (134.14, 245.94)	151.14 (112.49, 203.09)	139.91 (101.30, 193.22)	190.79 (146.44, 248.58)
GMFR (95% CI)	3.25 (2.33, 4.54)	3.84 (2.79, 5.27)	3.22 (2.34, 4.43)	3.75 (2.79, 5.05)	4.07 (3.07, 5.40)	2.78 (2.12, 3.64)	3.58 (2.68, 4.78)	4.02 (2.95, 5.49)
Participants with seroconversion	25 (43.9) (30.74, 57.64)	27 (46.6) (33.34, 60.13)	23 (41.1) (28.10, 55.02)	28 (51.9) (37.84, 65.66)	33 (55.9) (42.40, 68.84)	19 (31.1) (19.90, 44.29)	29 (49.2) (35.89, 62.50)	30 (48.4) (35.50, 61.44)

— no. (%) (95% CI)								
**Day 29**								
no.	65	61	63	60	61	66	63	66
GMT (95% CI)	354.11 (269.13, 465.92)	316.36 (243.84, 410.45)	263.95 (206.13, 338.00)	255.44 (181.20, 360.09)	300.62 (228.60, 395.34)	239.78 (180.19, 319.07)	211.86 (152.96, 293.44)	283.59 (222.40, 361.62)
GMFR (95% CI)	6.97 (5.05, 9.61)	7.73 (5.90, 10.13)	6.53 (4.74, 9.00)	7.68 (5.75, 10.28)	6.98 (5.58, 8.73)	4.44 (3.28, 6.01)	5.45 (4.12, 7.20)	5.93 (4.42, 7.96)
Participants with seroconversion — no. (%) (95% CI)	45 (69.2) (56.55, 80.09)	45 (73.8) (60.93, 84.20)	41 (65.1) (52.03, 76.66)	46 (76.7) (63.96, 86.62)	50 (82.0) (70.02, 90.64)	33 (50.0) (37.43, 62.57)	38 (60.3) (47.20, 72.43)	43 (65.2) (52.42, 76.47)
**Day 181**								
no.	59	61	60	60	61	64	63	62

GMT (95% CI)	178.85 (126.64, 252.58)	131.90 (97.43, 178.57)	116.43 (88.30, 153.51)	111.81 (78.03, 160.20)	131.15 (98.53, 174.58)	149.90 (111.76, 201.04)	134.16 (97.23, 185.12)	146.30 (112.22, 190.74)
GMFR (95% CI)	3.37 (2.44, 4.67)	3.29 (2.49, 4.34)	3.01 (2.27, 3.98)	3.46 (2.64, 4.52)	3.12 (2.50, 3.90)	2.69 (2.04, 3.55)	3.65 (2.71, 4.92)	2.80 (2.11, 3.71)
Participants with seroconversion — no. (%) (95% CI)	28 (49.1) (35.63, 62.71)	22 (36.7) (24.59, 50.10)	24 (42.1) (29.14, 55.92)	25 (43.9) (30.74, 57.64)	30 (50.0) (36.81, 63.19)	22 (34.4) (22.95, 47.30)	27 (45.0) (32.12, 58.39)	24 (39.3) (27.07, 52.69)
**B/Victoria: anti-HA antibodies by HAI assay**								
**Baseline (day 1)**								
no.	65	61	63	60	61	67	63	66
GMT (95% CI)	44.01 (33.60, 57.64)	35.69 (25.83, 49.31)	36.61 (27.12, 49.41)	35.19 (26.36, 46.98)	31.86 (24.75, 41.02)	45.02 (34.27, 59.15)	44.13 (32.61, 59.73)	36.73 (27.76, 48.61)
**Day 8**								

no.	59	60	59	58	60	61	62	63
GMT (95% CI)	140.58 (106.39, 185.76)	80.91 (60.90, 107.49)	83.35 (63.73, 109.00)	101.00 (79.71, 127.98)	91.30 (71.16, 117.13)	93.28 (73.73, 117.99)	114.40 (91.42, 143.16)	100.25 (82.12, 122.40)
GMFR (95% CI)	2.92 (2.08, 4.09)	2.25 (1.75, 2.91)	2.31 (1.89, 2.81)	2.69 (2.12, 3.42)	2.73 (2.19, 3.41)	2.28 (1.86, 2.80)	2.55 (2.04, 3.18)	2.83 (2.21, 3.63)
Participants with seroconversion — no. (%) (95% CI)	20 (35.1) (22.91, 48.87)	14 (24.1) (13.87, 37.17)	16 (28.6) (17.30, 42.21)	21 (38.9) (25.92, 53.12)	23 (39.0) (26.55, 52.56)	16 (26.2) (15.80, 39.07)	20 (33.9) (22.08, 47.39)	24 (38.7) (26.60, 51.93)
**Day 29**								
no.	65	61	63	60	61	67	63	66
GMT (95% CI)	151.68 (116.74, 197.06)	88.57 (69.14, 113.47)	86.88 (68.90, 109.55)	82.86 (64.68, 106.13)	75.56 (58.47, 97.65)	86.86 (70.52, 106.99)	105.92 (85.24, 131.60)	91.71 (73.63, 114.23)
GMFR (95% CI)	3.45	2.48	2.37	2.35	2.37	1.93	2.40	2.50

	(2.62, 4.54)	(1.98, 3.12)	(2.01, 2.80)	(1.99, 2.78)	(1.94, 2.90)	(1.60, 2.32)	(1.92, 3.01)	(2.08, 3.00)
Participants with seroconversion — no. (%) (95% CI)	30 (46.2) (33.70, 58.97)	12 (19.7) (10.60, 31.84)	18 (28.6) (17.89, 41.35)	15 (25.0) (14.72, 37.86)	14 (23.0) (13.15, 35.50)	10 (14.9) (7.40, 25.74)	19 (30.2) (19.23, 43.02)	17 (25.8) (15.78, 38.01)
**Day 181**								
no.	59	61	60	60	61	64	63	62
GMT (95% CI)	98.26 (73.80, 130.81)	64.82 (49.01, 85.73)	80.49 (61.62, 105.14)	74.64 (56.65, 98.34)	55.26 (42.76, 71.41)	83.56 (65.97, 105.82)	91.80 (72.47, 116.28)	63.97 (50.20, 81.52)
GMFR (95% CI)	2.13 (1.67, 2.71)	1.88 (1.45, 2.43)	2.05 (1.67, 2.53)	2.03 (1.71, 2.40)	1.79 (1.45, 2.22)	1.83 (1.47, 2.26)	2.14 (1.70, 2.70)	1.78 (1.51, 2.08)
Participants with seroconversion — no. (%) (95% CI)	14 (24.6) (14.13, 37.76)	11 (18.3) (9.52, 30.44)	14 (24.6) (14.13, 37.76)	13 (22.8) (12.74, 35.84)	10 (16.7) (8.29, 28.52)	15 (23.4) (13.75, 35.69)	15 (25.0) (14.72, 37.86)	9 (14.8) (6.98, 26.17)

B/Yamagata: anti-HA antibodies by HAI assay								
**Baseline (Day 1)**								
no.	65	61	63	60	61	67	63	66
GMT (95% CI)	73.82 (54.66, 99.71)	78.63 (57.35, 107.83)	68.94 (51.42, 92.43)	67.67 (49.44, 92.62)	60.20 (44.15, 82.07)	67.78 (50.98, 90.12)	74.88 (56.03, 100.07)	85.61 (67.38, 108.76)
**Day 8**								
no.	59	60	59	57	60	61	62	63
GMT (95% CI)	250.04 (195.63, 319.58)	188.07 (141.60, 249.80)	171.63 (132.14, 222.91)	227.71 (181.02, 286.45)	209.90 (162.24, 271.56)	138.81 (111.32, 173.07)	195.70 (153.33, 249.78)	216.55 (176.59, 265.56)
GMFR (95% CI)	3.03 (2.17, 4.22)	2.31 (1.76, 3.03)	2.73 (2.06, 3.60)	3.12 (2.38, 4.10)	3.41 (2.62, 4.44)	2.17 (1.77, 2.65)	2.52 (1.94, 3.26)	2.68 (2.17, 3.30)
Participants with seroconversion	18 (31.6) (19.91, 45.24)	18 (31.0) (19.54, 44.54)	21 (37.5) (24.92, 51.45)	23 (43.4) (29.84, 57.72)	26 (44.1) (31.16, 57.60)	19 (31.1) (19.90, 44.29)	21 (35.6) (23.55, 49.13)	24 (38.7) (26.60, 51.93)

— no. (%) (95% CI)								
**Day 29**								
no.	65	61	63	60	61	67	63	66
GMT (95% CI)	277.06 (225.13, 340.96)	245.01 (196.73, 305.14)	249.81 (199.09, 313.45)	246.78 (196.09, 310.57)	243.62 (196.35, 302.26)	191.74 (157.35, 233.65)	237.78 (194.08, 291.31)	255.31 (216.23, 301.44)
GMFR (95% CI)	3.75 (2.89, 4.88)	3.12 (2.49, 3.90)	3.62 (2.88, 4.57)	3.65 (2.94, 4.53)	4.05 (3.25, 5.04)	2.83 (2.31, 3.47)	3.18 (2.64, 3.81)	2.98 (2.46, 3.61)
Participants with seroconversion — no. (%) (95% CI)	31 (47.7) (35.15, 60.46)	22 (36.1) (24.16, 49.37)	33 (52.4) (39.41, 65.12)	27 (45.0) (32.12, 58.39)	33 (54.1) (40.85, 66.94)	25 (37.3) (25.80, 49.99)	26 (41.3) (29.01, 54.38)	27 (40.9) (28.95, 53.71)
**Day 181**								
no.	59	61	60	60	61	64	63	62
GMT (95% CI)	134.94	104.47	113.17	113.74	93.22	90.12	105.33	106.39

	(103.17, 176.51)	(82.63, 132.09)	(87.90, 145.70)	(88.60, 146.02)	(72.19, 120.38)	(69.17, 117.42)	(84.91, 130.68)	(86.72, 130.52)
GMFR (95% CI)	1.62 (1.25, 2.10)	1.36 (1.06, 1.74)	1.67 (1.35, 2.06)	1.57 (1.31, 1.88)	1.53 (1.22, 1.92)	1.36 (1.05, 1.76)	1.45 (1.16, 1.80)	1.26 (1.05, 1.51)
Participants with seroconversion — no. (%) (95% CI)	13 (22.8) (12.74, 35.84)	7 (11.7) (4.82, 22.57)	11 (19.3) (10.05, 31.91)	8 (14.0) (6.26, 25.79)	9 (15.0) (7.10, 26.57)	11 (17.2) (8.90, 28.68)	8 (13.3) (5.94, 24.59)	4 (6.6) (1.82, 15.95)

GMFR = geometric mean fold rise, GMT = geometric mean titer, HAI = hemagglutination inhibition.

Supplementary Table 4. Summary of neuraminidase antibody responses by neuraminidase inhibition assay through day 181 (per-protocol population)

	Recombinant vaccine	mRNA-1010	mRNA-1020			mRNA-1030		
		50 µg	50 µg	100 µg	150 µg	25 µg	50 µg	100 µg
	(*n* = 67)	(*n* = 63)	(*n* = 66)	(*n* = 64)	(*n* = 63)	(*n* = 67)	(*n* = 66)	(*n* = 67)
**A/H1N1: anti-N1 antibodies by NAI assay**								
**Baseline (day 1)**								
no.	67	63	66	64	63	67	66	67
GMT (95% CI)	102.55 (89.99, 116.86)	97.52 (88.57, 107.38)	100.79 (87.80, 115.71)	84.45 (79.22, 90.03)	112.52 (94.00, 134.67)	114.91 (97.97, 134.77)	107.35 (94.88, 121.46)	97.38 (88.80, 106.78)
**Day 8**								
no.	66	63	65	64	63	67	66	67
GMT (95% CI)	109.63 (92.53, 129.89)	96.45 (86.45, 107.62)	145.36 (119.15, 177.33)	136.01 (114.31, 161.83)	184.60 (148.17, 229.99)	119.76 (102.45, 140.00)	124.35 (108.21, 142.90)	114.91 (100.62, 131.23)

GMFR (95% CI)	1.07 0.96, 1.18)	0.99 (0.94, 1.04)	1.44 (1.26, 1.64)	1.61 (1.37, 1.90)	1.64 (1.39, 1.93)	1.04 (0.98, 1.10)	1.16 (1.08, 1.24)	1.18 (1.09, 1.28)
Participants with ≥2-fold rise from baseline — no. (%) (95% CI)	4 (6.1) (1.68, 14.80)	2 (3.2) (0.39, 11.00)	13 (20.0) (11.10, 31.77)	12 (18.8) (10.08, 30.46)	13 (20.6) (11.47, 32.70)	2 (3.0) (0.36, 10.37)	5 (7.6) (2.51, 16.80)	5 (7.5) (2.47, 16.56)
Participants with ≥3-fold rise from baseline — no. (%) (95% CI)	1 (1.5) (0.04, 8.16)	0 (0.00, 5.69)	3 (4.6) (0.96, 12.90)	4 (6.3) (1.73, 15.24)	4 (6.3) (1.76, 15.47)	0 (0.00, 5.36)	0 (0.00, 5.44)	2 (3.0) (0.36, 10.37)
Participants with ≥4-fold	1 (1.5) (0.04,	0 (0.00,	3 (4.6) (0.96,	4 (6.3) (1.73,	4 (6.3) (1.76,	0 (0.00,	0 (0.00,	2 (3.0) (0.36,

rise from baseline — no. (%) (95% CI)	8.16)	5.69)	12.90)	15.24)	15.47)	5.36)	5.44)	10.37)
**Day 29**								
no.	67	63	66	64	63	67	66	67
GMT (95% CI)	103.61 (91.50, 117.33)	97.52 (88.13, 107.91)	219.26 (174.90, 274.86)	198.70 (160.51, 245.98)	268.35 (210.93, 341.40)	163.35 (134.09, 198.99)	195.34 (159.57, 239.11)	170.25 (141.09, 205.43)
GMFR (95% CI)	1.01 (0.93, 1.10)	1.00 (0.96, 1.05)	2.18 (1.80, 2.63)	2.35 (1.92, 2.89)	2.38 (1.96, 2.90)	1.42 (1.23, 1.65)	1.82 (1.57, 2.11)	1.75 (1.49, 2.05)
Participants with ≥2-fold rise from baseline —	1 (1.5) (0.04, 8.04)	1 (1.6) (0.04, 8.53)	27 (40.9) (28.95, 53.71)	26 (40.6) (28.51, 53.63)	30 (47.6) (34.88, 60.59)	14 (20.9) (11.92, 32.57)	27 (40.9) (28.95, 53.71)	24 (35.8) (24.47, 48.47)

no. (%) (95% CI)								
Participants with ≥3-fold rise from baseline — no. (%) (95% CI)	0 (0.00, 5.36)	0 (0.00, 5.69)	12 (18.2) (9.76, 29.61)	11 (17.2) (8.90, 28.68)	13 (20.6) (11.47, 32.70)	4 (6.0) (1.65, 14.59)	7 (10.6) (4.37, 20.64)	6 (9.0) (3.36, 18.48)
Participants with ≥4-fold rise from baseline — no. (%) (95% CI)	0 (0.00, 5.36)	0 (0.00, 5.69)	12 (18.2) (9.76, 29.61)	11 (17.2) (8.90, 28.68)	13 (20.6) (11.47, 32.70)	4 (6.0) (1.65, 14.59)	7 (10.6) (4.37, 20.64)	6 (9.0) (3.36, 18.48)
**Day 181**								

no.	59	62	60	60	61	64	63	62
GMT (95% CI)	104.82 (88.93, 123.55)	93.55 (85.29, 102.62)	149.29 (118.89, 187.46)	126.99 (108.93, 148.05)	161.83 (130.05, 201.37)	136.01 (112.81, 163.97)	151.44 (125.37, 182.92)	135.30 (114.76, 159.51)
GMFR (95% CI)	1.00 (0.94, 1.07)	0.97 (0.92, 1.02)	1.52 (1.26, 1.82)	1.50 (1.30, 1.73)	1.47 (1.28, 1.69)	1.20 (1.07, 1.36)	1.39 (1.22, 1.58)	1.43 (1.25, 1.64)
Participants with ≥2-fold rise from baseline — no. (%) (95% CI)	2 (3.4) (0.41, 11.71)	1 (1.6) (0.04, 8.66)	15 (25.0) (14.72, 37.86)	9 (15.0) (7.10, 26.57)	13 (21.3) (11.86, 33.68)	10 (15.6) (7.76, 26.86)	16 (25.4) (15.27, 37.94)	15 (24.2) (14.22, 36.74)
Participants with ≥3-fold rise from baseline —	0 (0.00, 6.06)	0 (0.00, 5.78)	5 (8.3) (2.76, 18.39)	2 (3.3) (0.41, 11.53)	2 (3.3) (0.40, 11.35)	3 (4.7) (0.98, 13.09)	3 (4.8) (0.99, 13.29)	2 (3.2) (0.39, 11.17)

no. (%) (95% CI)								
Participants with ≥4-fold rise from baseline — no. (%) (95% CI)	0 (0.00, 6.06)	0 (0.00, 5.78)	5 (8.3) (2.76, 18.39)	2 (3.3) (0.41, 11.53)	2 (3.3) (0.40, 11.35)	3 (4.7) (0.98, 13.09)	3 (4.8) (0.99, 13.29)	2 (3.2) (0.39, 11.17)
**A/H3N2: anti-N2 antibodies by NAI assay**								
**Baseline (day 1)**								
no.	67	63	66	64	63	67	66	67
GMT (95% CI)	21.81 (17.15, 27.73)	19.75 (15.92, 24.49)	22.82 (17.83, 29.22)	20.30 (15.60, 26.41)	17.36 (14.30, 21.08)	21.75 (17.15, 27.57)	21.78 (16.99, 27.91)	20.55 (16.39, 25.76)
**Day 8**								
no.	66	63	66	64	63	67	66	67

GMT (95% CI)	26.70 (20.25, 35.21)	19.59 (15.71, 24.42)	64.20 (44.15, 93.35)	74.58 (51.72, 107.55)	79.31 (57.39, 109.59)	55.15 (39.90, 76.24)	62.87 (45.00, 87.83)	73.44 (53.46, 100.89)
GMFR (95% CI)	1.21 (1.01, 1.46)	0.99 (0.90, 1.09)	2.81 (2.17, 3.64)	3.67 (2.69, 5.01)	4.57 (3.32, 6.28)	2.54 (1.97, 3.26)	2.89 (2.20, 3.78)	3.57 (2.72, 4.69)
Participants with ≥2-fold rise from baseline — no. (%) (95% CI)	8 (12.1) (5.38, 22.49)	2 (3.2) (0.39, 11.00)	30 (45.5) (33.14, 58.19)	39 (60.9) (47.93, 72.90)	40 (63.5) (50.40, 75.27)	29 (43.3) (31.22, 55.96)	36 (54.5) (41.81, 66.86)	40 (59.7) (47.00, 71.51)
Participants with ≥3-fold rise from baseline —	3 (4.5) (0.95, 12.71)	0 (0.00, 5.69)	25 (37.9) (26.22, 50.66)	32 (50.0) (37.23, 62.77)	37 (58.7) (45.62, 70.99)	20 (29.9) (19.28, 42.27)	29 (43.9) (31.74, 56.70)	30 (44.8) (32.60, 57.42)

no. (%) (95% CI)								
Participants with ≥4-fold rise from baseline — no. (%) (95% CI)	3 (4.5) (0.95, 12.71)	0 (0.00, 5.69)	18 (27.3) (17.03, 39.64)	24 (37.5) (25.70, 50.49)	26 (41.3) (29.01, 54.38)	14 (20.9) (11.92, 32.57)	21 (31.8) (20.89, 44.44)	20 (29.9) (19.28, 42.27)
**Day 29**								
no.	67	63	66	64	63	67	66	66
GMT (95% CI)	20.49 (16.47, 25.51)	21.09 (16.44, 27.05)	105.23 (78.54, 141.00)	127.60 (92.74, 175.55)	139.16 (104.11, 186.00)	69.21 (50.36, 95.12)	79.56 (57.45, 110.18)	92.57 (70.53, 121.49)
GMFR (95% CI)	0.94 (0.84, 1.05)	1.07 (0.92, 1.24)	4.61 (3.64, 5.84)	6.29 (4.80, 8.23)	8.01 (6.04, 10.64)	3.18 (2.41, 4.21)	3.65 (2.79, 4.79)	4.47 (3.43, 5.83)

Participants with ≥2-fold rise from baseline — no. (%) (95% CI)	1 (1.5) (0.04, 8.04)	4 (6.3) (1.76, 15.47)	43 (65.2) (52.42, 76.47)	50 (78.1) (66.03, 87.49)	48 (76.2) (63.79, 86.02)	38 (56.7) (44.04, 68.78)	40 (60.6) (47.81, 72.42)	47 (71.2) (58.75, 81.70)
Participants with ≥3-fold rise from baseline — no. (%) (95% CI)	0 (0.00, 5.36)	2 (3.2) (0.39, 11.00)	39 (59.1) (46.29, 71.05)	44 (68.8) (55.94, 79.76)	45 (71.4) (58.65, 82.11)	30 (44.8) (32.60, 57.42)	35 (53.0) (40.34, 65.44)	40 (60.6) (47.81, 72.42)
Participants with ≥4-fold rise from baseline —	0 (0.00, 5.36)	2 (3.2) (0.39, 11.00)	29 (43.9) (31.74, 56.70)	34 (53.1) (40.23, 65.72)	38 (60.3) (47.20, 72.43)	20 (29.9) (19.28, 42.27)	25 (37.9) (26.22, 50.66)	24 (36.4) (24.87, 49.13)

no. (%) (95% CI)								
**Day 181**								
no.	59	61	60	59	61	64	63	62
GMT (95% CI)	21.34 (16.79, 27.12)	19.13 (15.27, 23.97)	50.43 (35.78, 71.07)	64.15 (46.15, 89.18)	64.08 (45.54, 90.17)	50.91 (37.88, 68.41)	50.86 (35.48, 72.91)	69.34 (49.33, 97.47)
GMFR (95% CI)	1.03 (0.85, 1.24)	0.98 (0.85, 1.14)	2.33 (1.75, 3.10)	3.20 (2.46, 4.16)	3.65 (2.68, 4.96)	2.28 (1.80, 2.87)	2.27 (1.77, 2.92)	3.43 (2.45, 4.81)
Participants with ≥2-fold rise from baseline — no. (%) (95% CI)	4 (6.8) (1.88, 16.46)	3 (4.9) (1.03, 13.71)	24 (40.0) (27.56, 53.46)	31 (52.5) (39.12, 65.70)	30 (49.2) (36.14, 62.30)	25 (39.1) (27.10, 52.07)	25 (39.7) (27.57, 52.80)	33 (53.2) (40.12, 66.02)

Participants with ≥3-fold rise from baseline — no. (%) (95% CI)	2 (3.4) (0.41, 11.71)	2 (3.3) (0.40, 11.35)	20 (33.3) (21.69, 46.69)	25 (42.4) (29.61, 55.93)	27 (44.3) (31.55, 57.55)	18 (28.1) (17.60, 40.76)	19 (30.2) (19.23, 43.02)	27 (43.5) (30.99, 56.74)
Participants with ≥4-fold rise from baseline — no. (%) (95% CI)	2 (3.4) (0.41, 11.71)	2 (3.3) (0.40, 11.35)	17 (28.3) (17.45, 41.44)	18 (30.5) (19.19, 43.87)	18 (29.5) (18.52, 42.57)	10 (15.6) (7.76, 26.86)	13 (20.6) (11.47, 32.70)	17 (27.4) (16.85, 40.23)
**B/Victoria: anti-NA antibodies by NAI assay**								
**Baseline (day 1)**								
no.	67	63	66	64	63	67	66	67

GMT (95% CI)	75.19 (63.70, 88.74)	79.12 (67.54, 92.70)	72.02 (61.80, 83.94)	70.25 (58.86, 83.84)	72.46 (60.37, 86.96)	75.19 (63.87, 88.51)	73.55 (62.53, 86.52)	73.65 (63.66, 85.20)
**Day 8**								
no.	66	63	65	64	63	67	66	67
GMT (95% CI)	73.55 (63.06, 85.79)	80.00 (67.77, 94.44)	281.56 (226.25, 350.40)	330.57 (267.90, 407.90)	365.16 (293.96, 453.61)	209.38 (176.83, 247.92)	238.47 (196.00, 290.15)	282.64 (238.72, 334.64)
GMFR (95% CI)	0.97 (0.88, 1.06)	1.01 (0.92, 1.11)	3.96 (3.18, 4.93)	4.71 (3.79, 5.85)	5.04 (3.95, 6.43)	2.78 (2.27, 3.41)	3.24 (2.65, 3.97)	3.84 (3.15, 4.67)
Participants with ≥2-fold rise from baseline — no. (%) (95% CI)	2 (3.0) (0.37, 10.52)	6 (9.5) (3.58, 19.59)	57 (87.7) (77.18, 94.53)	54 (84.4) (73.14, 92.24)	55 (87.3) (76.50, 94.35)	50 (74.6) (62.51, 84.47)	51 (77.3) (65.30, 86.69)	56 (83.6) (72.52, 91.51)

Participants with ≥3-fold rise from baseline — no. (%) (95% CI)	0 (0.00, 5.44)	0 (0.00, 5.69)	29 (44.6) (32.27, 57.47)	39 (60.9) (47.93, 72.90)	36 (57.1) (44.05, 69.54)	24 (35.8) (24.47, 48.47)	24 (36.4) (24.87, 49.13)	37 (55.2) (42.58, 67.40)
Participants with ≥4-fold rise from baseline — no. (%) (95% CI)	0 (0.00, 5.44)	0 (0.00, 5.69)	29 (44.6) (32.27, 57.47)	39 (60.9) (47.93, 72.90)	36 (57.1) (44.05, 69.54)	24 (35.8) (24.47, 48.47)	24 (36.4) (24.87, 49.13)	37 (55.2) (42.58, 67.40)
**Day 29**								
no.	67	63	66	64	63	67	66	67
GMT (95% CI)	69.93 (60.15,	78.26 (66.50,	416.08 (354.45,	477.73 (402.55,	470.32 (390.92,	268.39 (232.82,	316.66 (269.21,	316.71 (270.66,

	81.31)	92.10)	488.42)	566.95)	565.83)	309.40)	372.46)	370.58)
GMFR (95% CI)	0.93 (0.86, 1.01)	0.99 (0.90, 1.08)	5.78 (4.80, 6.95)	6.80 (5.67, 8.16)	6.49 (5.43, 7.76)	3.57 (2.95, 4.32)	4.31 (3.63, 5.10)	4.30 (3.64, 5.08)
Participants with ≥2-fold rise from baseline — no. (%) (95% CI)	2 (3.0) (0.36, 10.37)	5 (7.9) (2.63, 17.56)	63 (95.5) (87.29, 99.05)	63 (98.4) (91.60, 99.96)	63 (100) (94.31, 100.00)	56 (83.6) (72.52, 91.51)	62 (93.9) (85.20, 98.32)	62 (92.5) (83.44, 97.53)
Participants with ≥3-fold rise from baseline — no. (%) (95% CI)	0 (0.00, 5.36)	1 (1.6) (0.04, 8.53)	48 (72.7) (60.36, 82.97)	52 (81.3) (69.54, 89.92)	47 (74.6) (62.06, 84.73)	34 (50.7) (38.24, 63.18)	37 (56.1) (43.30, 68.26)	43 (64.2) (51.53, 75.53)

Participants with ≥4-fold rise from baseline — no. (%) (95% CI)	0 (0.00, 5.36)	1 (1.6) (0.04, 8.53)	48 (72.7) (60.36, 82.97)	52 (81.3) (69.54, 89.92)	47 (74.6) (62.06, 84.73)	34 (50.7) (38.24, 63.18)	37 (56.1) (43.30, 68.26)	43 (64.2) (51.53, 75.53)
**Day 181**								
no.	59	62	60	60	61	64	63	62
GMT (95% CI)	82.87 (69.08, 99.41)	79.11 (67.14, 93.21)	236.98 (194.79, 288.30)	251.07 (205.54, 306.67)	214.99 (173.21, 266.86)	161.74 (136.79, 191.24)	195.04 (161.84, 235.06)	182.97 (150.40, 222.60)
GMFR (95% CI)	1.06 (0.93, 1.21)	1.00 (0.92, 1.08)	3.36 (2.78, 4.07)	3.52 (2.89, 4.30)	3.01 (2.48, 3.66)	2.16 (1.79, 2.60)	2.69 (2.33, 3.12)	2.45 (2.12, 2.82)
Participants with ≥2-fold rise from	6 (10.2) (3.82, 20.83)	4 (6.5) (1.79, 15.70)	48 (80.0) (67.67, 89.22)	43 (71.7) (58.56, 82.55)	47 (77.0) (64.50, 86.85)	38 (59.4) (46.37, 71.49)	48 (76.2) (63.79, 86.02)	45 (72.6) (59.77, 83.15)

baseline — no. (%) (95% CI)								
Participants with ≥3-fold rise from baseline — no. (%) (95% CI)	1 (1.7) (0.04, 9.09)	0 (0.00, 5.78)	24 (40.0) (27.56, 53.46)	27 (45.0) (32.12, 58.39)	21 (34.4) (22.73, 47.69)	11 (17.2) (8.90, 28.68)	12 (19.0) (10.25, 30.91)	13 (21.0) (11.66, 33.18)
Participants with ≥4-fold rise from baseline — no. (%) (95% CI)	1 (1.7) (0.04, 9.09)	0 (0.00, 5.78)	24 (40.0) (27.56, 53.46)	27 (45.0) (32.12, 58.39)	21 (34.4) (22.73, 47.69)	11 (17.2) (8.90, 28.68)	12 (19.0) (10.25, 30.91)	13 (21.0) (11.66, 33.18)
**B/Yamagata: anti-NA antibodies by NAI assay**								

**Baseline (day 1)**								
no.	67	63	66	64	63	67	66	67
GMT (95% CI)	183.03 (144.84, 231.30)	227.52 (185.74, 278.70)	191.27 (156.42, 233.90)	186.20 (149.55, 231.82)	170.92 (135.25, 215.99)	198.83 (159.77, 247.43)	210.24 (169.52, 260.74)	205.09 (169.98, 247.46)
**Day 8**								
no.	66	63	66	64	63	67	66	67
GMT (95% CI)	208.04 (162.96, 265.59)	220.14 (180.17, 268.96)	914.65 (749.75, 1115.83)	1064.75 (879.83, 1288.55)	1085.27 (873.94, 1347.70)	640.00 (546.19, 749.92)	823.47 (696.04, 974.22)	828.90 (698.48, 983.68)
GMFR (95% CI)	1.12 (0.97, 1.30)	0.97 (0.87, 1.08)	4.78 (3.72, 6.14)	5.72 (4.45, 7.35)	6.35 (4.76, 8.46)	3.22 (2.64, 3.92)	3.92 (3.13, 4.91)	4.04 (3.27, 4.99)
Participants with ≥2-fold rise from baseline —	6 (9.1) (3.41, 18.74)	6 (9.5) (3.58, 19.59)	58 (87.9) (77.51, 94.62)	59 (92.2) (82.70, 97.41)	59 (93.7) (84.53, 98.24)	56 (83.6) (72.52, 91.51)	60 (90.9) (81.26, 96.59)	58 (86.6) (76.03, 93.67)

no. (%) (95% CI)								
Participants with ≥3-fold rise from baseline — no. (%) (95% CI)	2 (3.0) (0.37, 10.52)	0 (0.00, 5.69)	47 (71.2) (58.75, 81.70)	47 (73.4) (60.91, 83.70)	49 (77.8) (65.54, 87.28)	36 (53.7) (41.12, 66.00)	37 (56.1) (43.30, 68.26)	43 (64.2) (51.53, 75.53)
Participants with ≥4-fold rise from baseline — no. (%) (95% CI)	2 (3.0) (0.37, 10.52)	0 (0.00, 5.69)	47 (71.2) (58.75, 81.70)	47 (73.4) (60.91, 83.70)	49 (77.8) (65.54, 87.28)	36 (53.7) (41.12, 66.00)	37 (56.1) (43.30, 68.26)	43 (64.2) (51.53, 75.53)
**Day 29**								
no.	67	63	66	64	63	67	66	67

GMT (95% CI)	183.03 (146.21, 229.13)	215.34 (174.21, 266.19)	1293.51 (1099.70, 1521.48)	1441.95 (1215.88, 1710.05)	1428.87 (1186.31, 1721.02)	837.52 (718.85, 975.80)	953.89 (823.81, 1104.52)	998.57 (857.69, 1162.59)
GMFR (95% CI)	1.00 (0.92, 1.09)	0.95 (0.86, 1.05)	6.76 (5.41, 8.46)	7.74 (6.35, 9.45)	8.36 (6.82, 10.24)	4.21 (3.48, 5.10)	4.54 (3.79, 5.43)	4.87 (4.07, 5.82)
Participants with ≥2-fold rise from baseline — no. (%) (95% CI)	5 (7.5) (2.47, 16.56)	4 (6.3) (1.76, 15.47)	65 (98.5) (91.84, 99.96)	64 (100) (94.40, 100.00)	63 (100) (94.31, 100.00)	62 (92.5) (83.44, 97.53)	66 (100) (94.56, 100.00)	66 (98.5) (91.96, 99.96)
Participants with ≥3-fold rise from baseline —	1 (1.5) (0.04, 8.04)	1 (1.6) (0.04, 8.53)	55 (83.3) (72.13, 91.38)	58 (90.6) (80.70, 96.48)	60 (95.2) (86.71, 99.01)	46 (68.7) (56.16, 79.44)	45 (68.2) (55.56, 79.11)	54 (80.6) (69.11, 89.24)

no. (%) (95% CI)								
Participants with ≥4-fold rise from baseline — n (%) (95% CI)	1 (1.5) (0.04, 8.04)	1 (1.6) (0.04, 8.53)	55 (83.3) (72.13, 91.38)	58 (90.6) (80.70, 96.48)	60 (95.2) (86.71, 99.01)	46 (68.7) (56.16, 79.44)	45 (68.2) (55.56, 79.11)	54 (80.6) (69.11, 89.24)
**Day 181**								
no.	59	61	60	60	61	64	63	62
GMT (95% CI)	193.09 (150.62, 247.53)	212.57 (173.00, 261.18)	625.38 (517.53, 755.70)	693.91 (561.72, 857.20)	604.65 (489.74, 746.53)	414.99 (343.55, 501.28)	536.70 (450.77, 639.00)	529.22 (436.91, 641.05)
GMFR (95% CI)	1.02 (0.89, 1.18)	0.96 (0.85, 1.07)	3.44 (2.75, 4.31)	3.65 (3.03, 4.39)	3.61 (2.90, 4.50)	2.11 (1.81, 2.47)	2.63 (2.25, 3.09)	2.53 (2.17, 2.95)
Participants with ≥2-fold	8 (13.6) (6.04,	3 (4.9) (1.03,	53 (88.3) (77.43,	56 (93.3) (83.80,	56 (91.8) (81.90,	46 (71.9) (59.24,	54 (85.7) (74.61,	55 (88.7) (78.11,

rise from baseline — no. (%) (95% CI)	24.98)	13.71)	95.18)	98.15)	97.28)	82.40)	93.25)	95.34)
Participants with ≥3-fold rise from baseline — no. (%) (95% CI)	2 (3.4) (0.41, 11.71)	1 (1.6) (0.04, 8.80)	32 (53.3) (40.00, 66.33)	32 (53.3) (40.00, 66.33)	33 (54.1) (40.85, 66.94)	16 (25.0) (15.02, 37.40)	22 (34.9) (23.34, 47.97)	21 (33.9) (22.33, 47.01)
Participants with ≥4-fold rise from baseline — no. (%) (95% CI)	2 (3.4) (0.41, 11.71)	1 (1.6) (0.04, 8.80)	32 (53.3) (40.00, 66.33)	32 (53.3) (40.00, 66.33)	33 (54.1) (40.85, 66.94)	16 (25.0) (15.02, 37.40)	22 (34.9) (23.34, 47.97)	21 (33.9) (22.33, 47.01)

GMFR = geometric mean fold rise, GMT = geometric mean titer, NAI = neuraminidase inhibition.

Supplementary Table 5. Approximate HA and NA content in each vaccine.

Vaccine	Approximate HA content (µg)[a]	Approximate NA content (µg)[a]
mRNA-1010 50 µg	50	0
mRNA-1020 50 µg	25	25
mRNA-1020 100 µg	50	50
mRNA-1020 150 µg	75	75
mRNA-1030 25 µg	19	6
mRNA-1030 50 µg	38	13
mRNA-1030 100 µg	75	25

[a]Approximate micrograms of combined HA and combined NA (A/Wisconsin/588/2019 [H1N1]; A/Cambodia/e0826360/2020 [H3N2]; B/Washington/02/2019 [B/Victoria lineage]; B/Phuket/3073/2013 [B/Yamagata lineage]). Rounded numbers may not add up to exact total dose.

HA = hemagglutinin, NA = neuraminidase.

Supplementary Fig. 1. Study arms.

Study arm	Vaccine	Total dose	Antigen(s)	Valency	Planned enrollment, n			Actual enrollment, n		
					Total	Aged 18-49 y	Aged 50-75 y	Total	Aged 18-49 y	Aged 50-75 y
1	mRNA-1020	50 µg	HA + NA	8	70	35	35	72	36	36
2	mRNA-1020	100 µg	HA + NA	8	70	35	35	71	35	36
3	mRNA-1020	150 µg	HA + NA	8	70	35	35	71	36	35
4	mRNA-1030	25 µg	HA + NA	8	70	35	35	71	35	36
5	mRNA-1030	50 µg	HA + NA	8	70	35	35	72	36	36
6	mRNA-1030	100 µg	HA + NA	8	70	35	35	72	36	36
7	mRNA-1010	50 µg	HA	4	70	35	35	71	35	36
8	Licensed recombinant vaccine	180 µg	HA	4	70	35	35	72	36	36

HA = hemagglutinin, NA = neuraminidase.

Supplementary References

1. Nachbagauer R, *et al*. A universal influenza virus vaccine candidate confers protection against pandemic H1N1 infection in preclinical ferret studies. *NPJ Vaccines* **2**, 26 (2017).

CLINICAL STUDY PROTOCOL

**Protocol Title:**	Phase 1/2, randomized, observer-blind, dose-ranging study to evaluate the safety, reactogenicity, and immunogenicity of mRNA-1020 and mRNA-1030 candidate seasonal influenza vaccines in healthy adults
**Protocol Number:**	mRNA-1020-P101
**Sponsor Name:**	ModernaTX, Inc.
**Legal Registered Address:**	200 Technology Square<br>Cambridge, MA 02139
**Sponsor Contact and Medical Monitor:**	PPD<br>ModernaTX, Inc.<br>200 Technology Square<br>Cambridge, MA 02139<br>Telephone: PPD<br>e-mail: PPD
**Regulatory Agency Identifier Number(s):**	IND: 28201
**Amendment Number:**	1
**Date of Amendment 1:**	16 Feb 2022
**Date of Original Protocol:**	21 Dec 2021

CONFIDENTIAL

All financial and nonfinancial support for this study will be provided by ModernaTX, Inc. The concepts and information contained in this document or generated during the study are considered proprietary and may not be disclosed in whole or in part without the expressed written consent of ModernaTX, Inc. The study will be conducted according to the *International Council for Harmonisation (ICH) of Technical Requirements for Registration of Pharmaceuticals for Human Use, E6(R2) Good Clinical Practice (GCP) Guidance.*

PROTOCOL APPROVAL – SPONSOR SIGNATORY

Study Title: Phase 1/2, randomized, observer-blind, dose-ranging study to evaluate the safety, reactogenicity, and immunogenicity of mRNA-1020 and mRNA-1030 candidate seasonal influenza vaccines in healthy adults

Amendment Number: 1

Approval Date: 16 Feb 2022

Protocol accepted and approved by:

See e-Signature and date at the end of the document.

PPD

Date

ModernaTX, Inc
200 Technology Square
Cambridge, MA 02139
Telephone: PPD

DECLARATION OF INVESTIGATOR

I have read and understood all sections of the protocol entitled Phase 1/2, randomized, observer-blind, dose-ranging study to evaluate the safety, reactogenicity, and immunogenicity of mRNA-1020 and mRNA-1030 candidate seasonal influenza vaccines in healthy adults and the most recent version of the investigator's brochure.

I agree to supervise all aspects of the protocol and to conduct the clinical investigation in accordance with the current Protocol, the *International Council for Harmonisation (ICH) of Technical Requirements for Registration of Pharmaceuticals for Human Use, E6(R2) Good Clinical Practice (GCP) Guidance*, and all applicable government regulations. I will not make changes to the protocol before consulting with ModernaTX, Inc. or implement protocol changes without IRB approval except to eliminate an immediate risk to participants.

I agree to administer study treatment only to participants under my personal supervision or the supervision of a subinvestigator. I will not supply study treatment to any person not authorized to receive it. I also agree that persons debarred from conducting or working on clinical studies by any court or regulatory agency will not be allowed to conduct or work on studies for the Sponsor or a partnership in which the Sponsor is involved. I will immediately disclose it in writing to the Sponsor if any person who is involved in the study is debarred, or if any proceeding for debarment is pending, or, to the best of my knowledge, threatened.

I will not disclose confidential information contained in this document including participant information, to anyone other than the recipient study staffs and members of the IRB. I agree to ensure that this information will not be used for any purpose other than the evaluation or conduct of the clinical investigation without the prior written consent from ModernaTX, Inc. I will not disclose information regarding this clinical investigation or publish results of the investigation without authorization from ModernaTX, Inc.

The signature below provides the necessary assurance that this study will be conducted according to all stipulations of the protocol, including statements regarding confidentiality, and according to local legal and regulatory requirements, US federal regulations, and ICH E6(R2) GCP guidelines.

_______________________________________ _______________________

Signature of Principal Investigator Date

____

Printed Name of Principal Investigator

PROTOCOL AMENDMENT SUMMARY OF CHANGES

DOCUMENT HISTORY	
**Document**	**Date**
Amendment 1	16 Feb 2022
Original Protocol	21 Dec 2021

Amendment 1, 16 Feb 2022: Current Amendment

This amendment is considered to be nonsubstantial based on the criteria set forth in Article 10(a) of Directive 2001/20/EC of the European Parliament and the Council of the European Union because it neither significantly impacts the safety or physical/mental integrity of participants nor the scientific value of the study.

Main Rationale for the Amendment:

The main purpose of this amendment is to enhance clarity and implement additional protocol administrative corrections.

The summary of changes table provided here describes the changes made in Amendment 1 relative to the original protocol, including the sections modified and the corresponding rationales. The synopsis of Amendment 1 has been modified to correspond to changes in the body of the protocol. Minor editorial and grammatical corrections were also made.

Summary of Major Changes from Original Protocol to Protocol Amendment 1:

Section # and Name	Description of Change	Brief Rationale
Section 1.2 (Schema)	• Figure updates	• Updated for alignment with other ongoing trials
Section 1.3 (Schedule of Events)	• Addition of injection site and axillary lymph node assessment (injected arm)	• Updated for alignment with other ongoing trials
Section 2.2.3 (Clinical Studies)	• Updated information for ongoing clinical trials	• To enhance clarity of the ongoing trials
Section 4.1 (General Design)	• New table presentation of the dose levels and their mass ratio	• To enhance clarity of dose levels
Section 5.1 (Inclusion Criteria)<br>Section 11.2 (Appendix 2: Contraceptive Guidance)	• Inclusion criterion 5 was updated to clarify the definition of nonchildbearing potential females added to Appendix 2	• Updated for alignment with other ongoing trials

Section # and Name	Description of Change	Brief Rationale
Section 5.2<br>(Exclusion Criteria)	• Exclusion criterion 1 window was updated to 10 days and to include a positive SARS-CoV-2 test restriction<br>• Exclusion criterion 4 was updated to include "Any change in management of medication within 60 days should be discussed with the study medical team."<br>• Exclusion criteria 11, 13, and 14 window was updated to 180 days	• Updated for alignment with other ongoing trials
Section 6.1<br>(Investigational Products Administered)	• Updated the concentration and sodium acetate molarity of mRNA-1010, mRNA-1020, and mRNA-1030 IP	• Added information missing from original protocol
Section 6.3.4<br>(Investigational Product Packaging and Labeling)	• The fill volume updated to 0.8 mL	• Clarification of current practice
Section 8.1.1<br>(Use of Electronic Diaries)<br>Section 8.4.3<br>(Solicited Adverse Reactions)	• eDiary text updates to reflect current practices	• Alignment with protocol checklist
Section 8.1.5<br>(Physical Examinations)	• Updated requirement to document any lymph node abnormalities for the injection arm	• Clarification of current practice
Section 8.1.7<br>(Assessments for Respiratory Viral Infections)	• Clarification of the window for NP swab collection	• Clarification of current practice
Throughout the Protocol	• Minor formatting and edits were made for consistency	• Minor formatting and edits were made for consistency and clarity

1. PROTOCOL SUMMARY

1.1. Synopsis

Name of Sponsor/Company: ModernaTX, Inc.

Name of Investigational Products: mRNA-1020, mRNA-1030, mRNA-1010

Protocol Title: Phase 1/2, randomized, observer-blind, dose-ranging study to evaluate the safety, reactogenicity, and immunogenicity of mRNA-1020 and mRNA-1030 candidate seasonal influenza vaccines in healthy adults

Protocol Number: mRNA-1020-P101 Amendment 1

Study Period: Up to 7 months

Phase of Development: Phase 1/2

Total Number of Sites: United States only with approximately 20 sites

Objectives and Endpoints

Objectives	Endpoints
**Primary**	
• To evaluate the safety and reactogenicity of mRNA-1020, mRNA-1030, and mRNA-1010	• Frequency and grade of each solicited local and systemic reactogenicity AR during a 7-day follow-up period post-vaccination<br>• Frequency and severity of any unsolicited AEs during the 28-day follow-up period post-vaccination<br>• Frequency of any SAEs, AESIs, MAAEs, and AEs leading to withdrawal from Day 1 through Day 181/EoS<br>• Safety laboratory abnormalities through 7 days post-vaccination
• To evaluate the humoral immunogenicity of mRNA-1020, mRNA-1030, and mRNA-1010 against vaccine-matched influenza A and B strains at Day 29	• GMT and GMFR, comparing Day 29 to Day 1 (baseline), and percentage of participants with seroconversion, defined as a Day 29 titer ≥ 1:40 if baseline is < 1:10 or a 4-fold or greater rise if baseline is ≥ 1:10 in anti-HA antibodies measured by HAI assay<br>• GMT and GMFR of anti-NA measured by NAI assay at Day 1 and Day 29 and percentage of participants with a change in the Day 29 titer of at least 2-/3-/4-fold rise, defined as ≥ 2-/3-/4-fold of the LLOQ if the

	Day 1 titer is < LLOQ; or ≥ 2-/3-/4-fold of the Day 1 titer if the Day 1 titer is ≥ LLOQ
**Secondary**	
• To evaluate the humoral immunogenicity of mRNA-1020, mRNA-1030, and mRNA-1010 against vaccine-matched influenza A and B strains at all evaluable humoral immunogenicity time points	• GMT and GMFR (compared to Day 1) of anti-HA or anti-NA antibodies as measured by HAI, NAI, and/or MN assays
**Exploratory (may be performed)**	
• To evaluate the humoral immunogenicity against vaccine-mismatched influenza A and B strains	• GMT and GMFR (compared to Day 1) of anti-HA or anti-NA antibodies as measured by HAI, NAI, or MN assays against vaccine-mismatched strains
• To evaluate cellular immunogenicity in a subset of participants	• Frequency, magnitude, and phenotype of virus-specific T-cell and B-cell responses measured by flow cytometry or other methods, and to perform targeted repertoire analysis of T-cells and B-cells after vaccination
• To further characterize antibody responses, for example, Fc-mediated function, avidity, or epitope specificity	• Frequency, specificities, or other endpoints to be determined, for the further characterization of antibody responses
• To assess the occurrence of clinical influenza in study participants and characterize their immune response to infection and viral isolates	• Frequency of clinical influenza and immune responses
• Sample collection to perform passive transfer studies in preclinical animal models	• Transfer of human sera into mice with subsequent influenza virus challenge to observe protection from morbidity and mortality conferred by NA-specific antibodies

Abbreviations: AE = adverse event; AESI = adverse event of special interest; AR = adverse reaction; EoS = end of study; Fc = fragment crystallizable; GMFR = geometric mean fold rise; GMT = geometric mean titer; HA = hemagglutinin; HAI = hemagglutination inhibition; LLOQ = lower limit of quantification; MAAE = medically attended adverse event; MN = microneutralization; NA = neuraminidase; NAI = neuraminidase inhibition; SAE = serious adverse event.

Overall Study Design

The study will be a Phase 1/2, randomized, observer-blind, dose-ranging study to evaluate the safety, reactogenicity, and immunogenicity of mRNA-1020 (4 hemagglutinin [HA] and

4 neuraminidase [NA] at 1:1 HA:NA mass ratio) and mRNA-1030 (4 HA and 4 NA at 3:1 HA:NA mass ratio) in healthy adults 18 to 75 years of age.

The vaccines to be tested in the proposed Phase 1/2 study contain mRNAs encoding for the surface glycoproteins of the strains recommended by the World Health Organization (WHO) for 2021/22 Northern Hemisphere cell- or recombinant-based vaccines:

- A/Wisconsin/588/2019(H1N1)pdm09
- A/Cambodia/e0826360/2020(H3N2)
- B/Washington/02/2019 (B/Victoria lineage)
- B/Phuket/3073/2013 (B/Yamagata lineage)

Immunizations for the study are planned during the Northern Hemisphere spring, when influenza infection rates are typically low or declining. Approximately 560 participants will be randomized to one of 8 study arms to receive a single dose of either one of the 2 next-generation candidate vaccines (mRNA-1020 or mRNA-1030) at different dose levels, a single dose of the first-generation candidate vaccine (mRNA-1010), or Flublok, a licensed enhanced seasonal influenza vaccine comparator (8 study arms total). Three different dose levels (50, 100, and 150 µg total mRNA) of the 1:1 HA:NA mass ratio vaccine (mRNA-1020), 3 different dose levels (25, 50, and 100 µg total mRNA) of the 3:1 HA:NA mass ratio vaccine (mRNA-1030), and a single dose level of mRNA-1010 (50 µg total mRNA) will be assessed. The number of participants in each arm and the randomization ratio can be found in the table below.

Study Arms and Dose Levels

Vaccination Group	Group Name	Antigen(s)	Total Dose	N (total)	Age 18-49	Age 50-75
1	**mRNA-1030**	HA+NA	25 µg	70	35	35
2	**mRNA-1020**	HA+NA	50 µg	70	35	35
3	**mRNA-1030**	HA+NA	50 µg	70	35	35
4	**mRNA-1020**	HA+NA	100 µg	70	35	35
5	**mRNA-1030**	HA+NA	100 µg	70	35	35
6	**mRNA-1020**	HA+NA	150 µg	70	35	35
7	**mRNA-1010** (HA only comparator)	HA only	50 µg	70	35	35
8	**Flublok** (active comparator)	HA only	180 µg	70	35	35
Total				560	280	280

Abbreviations: HA = hemagglutinin; mRNA = messenger RNA; NA = neuraminidase.

Generally healthy adults (to include 2 age groups: 18 to < 50 years of age and ≥ 50 to ≤ 75 years of age) will be screened and enrolled. Participants with diagnoses or conditions requiring significant changes in management or medication within the 60 days prior to Day 1

will be excluded. Participants with immunocompromising conditions or medications, or malignancy within 10 years (excluding non-melanoma skin cancer) will also be excluded. Participants who received a licensed or investigational influenza vaccine within the prior 180 days or had tested positive for influenza by a Centers for Disease Control (CDC)-recommended testing method within 180 days prior to Day 1 will be excluded as well. See Study Eligibility Criteria for a complete listing of inclusion and exclusion criteria.

Enrollment will occur in parallel across all 8 study arms. The study will randomize approximately 70 healthy adult participants into each study arm with a balanced age group representation stratified approximately 1:1 into age groups of 18 to < 50 years of age and ≥ 50 to ≤ 75 years of age.

Except for appropriately delegated unblinded pharmacists, vaccine administrators, and monitors, all personnel involved in the conduct of the study will remain blinded to individual treatment assignment until study unblinding. Study visits will consist of a Screening Visit (up to 28 days before the Day 1 visit), Vaccination Visit at Day 1, and subsequent study visits on Day 4, Day 8, Day 29 (Month 1), and Day 181/end of study (EoS; Month 6), with up to 7 months of study participation. Unscheduled visits for potential influenza-like illness (ILI) symptoms will include testing with a multiplex respiratory infection panel (Biofire or similar).

Safety Oversight:

Safety monitoring for this study will include a group of blinded study team members, inclusive of, at a minimum, a Sponsor medical monitor, a contract research organization (CRO) medical monitor, a blinded internal safety team (IST), and an unblinded data safety monitoring board (DSMB). The study team will conduct ongoing blinded safety reviews during the study and will be responsible for notifying the IST and DSMB of potential safety signal events or the triggering of pause rules. An unblinded statistician will support the determination if study pause rules have been met.

The IST will comprise Sponsor physicians. The IST will conduct a scheduled review of safety data after approximately 96 participants (approximately 12 per study arm) (see the Study Arms and Dose Levels table above) have completed the Day 8 visit as well as ad hoc safety data reviews if requested by the study team. Enrollment will continue while these reviews are conducted if no pause rules have been met and the study team has not identified any safety concerns. The IST will also conduct ad hoc reviews as requested by the study medical monitor and the study team. The DSMB, composed of external/independent subject matter experts, will conduct unblinded reviews of safety data on an ad hoc basis if any pause rule is met or as otherwise requested by the study team and/or IST. Details regarding the IST and DSMB

composition, responsibilities, procedures, and frequency of data review will be defined in their respective charters.

Study Duration: Up to 7 months for each participant.

Number of Participants: Approximately 560 participants will be enrolled. See the Study Arms and Dose Levels table above for the number of participants in each cohort.

Study Eligibility Criteria:

Inclusion Criteria:

Each participant must meet all of the following criteria to be enrolled in this study:

1. Adults 18 to 75 years of age at the time of consent (Screening Visit). Participants may be rescreened if they are not medically stable at the Screening Visit.
2. Investigator assessment that participant understands and is willing and physically able to comply with protocol-mandated follow-up, including all procedures.
3. Participant has provided written informed consent for participation in this study, including all evaluations and procedures as specified in this protocol.
4. Body mass index of 18 kg/m^2 to 35 kg/m^2 (inclusive) at the Screening Visit.
5. Female participants of nonchildbearing potential may be enrolled in the study. Nonchildbearing potential is defined as post-menopausal or permanently sterilized. A follicle-stimulating hormone level should be measured at the discretion of the Investigator, if necessary, to confirm postmenopausal status.
6. Female participants of childbearing potential may be enrolled in the study if the participant fulfills all the following criteria:
   - Has a negative pregnancy test at the Screening Visit and on the day of vaccination (Day 1).
   - Has practiced adequate contraception or has abstained from all activities that could result in pregnancy for at least 28 days prior to Day 1. Adequate female contraception is defined as consistent and correct use of a Food and Drug Administration approved contraceptive method in accordance with the product label.
   - Has agreed to continue adequate contraception through 90 days following vaccine administration.
   - Is not currently breastfeeding.

Exclusion Criteria:

Participants meeting any of the following criteria will be excluded from the study:

1. Participant has had close contact to someone with severe acute respiratory syndrome coronavirus 2 (SARS-CoV-2) infection or coronavirus disease 2019 (COVID-19) as defined by the CDC or has had a positive SARS-CoV-2 test in the past 10 days prior to the Screening Visit.
2. Clinical screening laboratory values (total white blood cell [WBC] count, hemoglobin, platelets, alanine aminotransferase, aspartate aminotransferase, creatinine, alkaline phosphatase, and total bilirubin) > Grade 1 in laboratory abnormality.
3. Participant is acutely ill or febrile (temperature ≥ 38.0°C/100.4°F) 72 hours prior to or at the Screening Visit or Day 1. Participants meeting this criterion may be rescheduled within the 28-day screening window and will retain their initially assigned participant number.
4. History of a diagnosis or condition that, in the judgment of the Investigator, is clinically unstable or may affect participant safety, assessment of safety endpoints, assessment of immune response, or adherence to study procedures. Clinically unstable is defined as a diagnosis or condition requiring significant changes in management or medication within the 60 days prior to screening and includes ongoing workup of an undiagnosed illness that could lead to a new diagnosis or condition.
5. History of myocarditis, pericarditis, or myopericarditis.
6. Reported history of congenital or acquired immunodeficiency, immunosuppressive condition, or immune-mediated disease.
7. Dermatologic conditions that could affect local solicited adverse reaction (AR) assessments (eg, tattoos, psoriasis patches affecting skin over the deltoid areas).
8. Reported history of anaphylaxis or severe hypersensitivity reaction after receipt of any components of mRNA- or cell-based influenza (eg, Flublok) vaccine.
9. Reported history of bleeding disorder that is considered a contraindication to intramuscular (IM) injection or phlebotomy.
10. Any medical, psychiatric, or occupational condition, including reported history of substance abuse, that, in the opinion of the Investigator, might pose additional risk due to participation in the study or could interfere with the interpretation of study results. Asymptomatic conditions and conditions with no evidence of end organ involvement (eg, mild hypertension, dyslipidemia) are not exclusionary, provided that they are being appropriately managed and are clinically stable (ie, unlikely to result in symptomatic illness within the time course of this study). Illnesses or conditions may be exclusionary, even if otherwise stable, due to therapies used to treat them (eg, immune-modifying treatments), at the discretion of the Investigator.
11. Participant has received systemic immunosuppressants or immune-modifying drugs for > 14 days in total within 180 days prior to screening (for corticosteroids ≥ 10 mg/day

of prednisone equivalent) or is anticipating the need for immunosuppressive treatment at any time during participation in the study.

12. Participant has received or plans to receive any licensed or authorized vaccine, including COVID-19 vaccines, ≤ 28 days prior to the study injection (Day 1), or plans to receive a licensed or authorized vaccine within 28 days after the study injection.
13. Participant has received a Northern Hemisphere 2021-2022 seasonal influenza vaccine or any other influenza vaccine within 180 days prior to Day 1.
14. Participant tested positive for influenza by CDC-recommended testing methods within 180 days prior to Day 1.
15. Participant has received systemic immunoglobulins or blood products within 90 days prior to the Screening Visit or plans to receive during the study.
16. Diagnosis of malignancy within previous 10 years (excluding nonmelanoma skin cancer).
17. Participant has donated ≥ 450 mL of blood products within 28 days prior to the Screening Visit or plans to donate blood products during the study.
18. Participated in an interventional clinical study within 28 days prior to the Screening Visit based on the medical history interview or plans to do so while participating in this study.
19. Participant is an immediate family member or household member of study personnel, study site staff, or Sponsor personnel.

Study Treatments:

Investigational Product, Dosage, and Mode of Administration: mRNA-1020, mRNA-1030, and mRNA-1010 investigational products (IPs) are lipid nanoparticle (LNP) dispersions encoding the seasonal influenza vaccine antigens, HA and NA (mRNA-1020 and mRNA-1030) or HA only (mRNA-1010) from influenza strains A/Wisconsin/588/2019(H1N1)pdm09, A/Cambodia/e0826360/2020(H3N2), B/Washington/02/2019 (B/Victoria lineage), and B/Phuket/3073/2013 (B/Yamagata lineage). mRNA-1020 contains a 1:1 mass ratio of HA and NA; mRNA-1030 contains a 3:1 mass ratio of HA and NA; and mRNA-1010 contains HA only.

All mRNAs are formulated in LNPs composed of 4 lipids are provided as sterile liquids for injection with a white-to-off white dispersion in appearance. mRNA-1020 and mRNA-1030 IP are filled at a concentration of 0.4 mg/mL [redacted]. mRNA-1010 IP is filled at a concentration of 0.4 mg/mL [redacted]

mRNA-1020 and mRNA-1030 IP will be administered as a single IM injection dose at mRNA total dose levels of 50, 100, 150 µg, and 25, 50, 100 µg, respectively. mRNA-1010 IP will be administered as a single IM injection dose at mRNA total dose level of 50 µg.Flublok, a licensed influenza vaccine, will be administered as single 0.5 mL IM injection.

Procedures and Assessments:

Safety Assessments:

Safety assessments will include monitoring and recording of the following for each participant:

- Solicited local and systemic ARs that occur during the 7 days following study vaccine administration (ie, the day of study injection and 6 subsequent days). Solicited ARs will be recorded daily using electronic diaries.
- Unsolicited adverse events (AEs) observed or reported during the 28 days following vaccine administration (ie, the day of study injection and 27 subsequent days).
- Serious AEs (SAEs), AEs of special interest (AESIs), medically attended AEs (MAAEs), and AEs leading to withdrawal from study participation from Day 1 through Day 181/EoS or withdrawal from the study.
- Results of safety laboratory tests.
- Vital sign measurements.
- Physical examination findings.
- Baseline electrocardiograms (ECGs) results.
- Assessments for causes of respiratory viral infection as needed from Day 1 through Day 181/EoS or through withdrawal from the study.
- Pregnancy and accompanying outcomes in female participants reported after administration of study vaccine.
- Concomitant medications and therapies.

Immunogenicity Assessments:

Blood samples for immunogenicity assessments will be collected at the time points indicated in the Schedule of Events. The following immunogenicity assessments are planned:

- Serum anti-HA antibody level as measured by hemagglutination inhibition assay (primary, secondary, and exploratory endpoints)
- NA-specific antibody level as measured by neuraminidase inhibition assay (primary, secondary, and exploratory endpoints)
- Serum neutralizing antibody level as measured by microneutralization (MN) assay or similar method (exploratory endpoint)
- Cellular immunogenicity (exploratory endpoint)

Clinical Assessments:

All participants will provide nasopharyngeal (NP) swab samples before injection on Day 1 for assessment of asymptomatic infection with respiratory pathogens, including influenza virus and SARS-CoV-2, as influenza or COVID-19 symptoms may confound reactogenicity assessment. In addition, participants who develop symptoms consistent with CDC-defined ILI and/or COVID-19 during the study should be tested by multiplex viral polymerase chain reaction (NP specimen).

Statistical Methods:

General Considerations: All analyses will be performed by treatment arm, unless otherwise specified. Categorical variables, frequencies, and percentages will be presented. Continuous variables will be summarized using descriptive statistics (number of participants, mean, median, standard deviation, minimum, and maximum).

Analysis Populations: The table below describes the analysis datasets.

Populations for Analyses

Set	Description
Randomization Set	The Randomization Set consists of all participants who are randomly assigned.
Full Analysis Set (FAS)[1]	The FAS consists of all randomly assigned participants who receive the IP.
Per-Protocol (PP) Set[2]	The PP Set consists of all participants in the FAS who comply with the injection schedule, comply with the timings of immunogenicity blood sampling to have a baseline and at least 1 postinjection assessment, do not have influenza infection at baseline through

	Day 29 (as documented by PCR testing), and have no major protocol deviations that impact the immune response.
Safety Set[3]	The Safety Set consists of all randomly assigned participants who receive the IP.
Solicited Safety Set[4]	The Solicited Safety Set consists of all participants in the Safety Set who contribute any solicited AR data.

Abbreviations: AR = adverse reaction; IP = investigational product; PCR = polymerase chain reaction.

1. For the FAS, participants will be analyzed according to the group to which they were randomized.
2. The PP Set will be used as the primary analysis set for analyses of immunogenicity unless otherwise specified. Participants will be analyzed according to the group to which they were randomized.
3. The Safety Set will be used for all analyses of safety, except for the solicited ARs. Participants will be included in the vaccination group corresponding to what they actually received.The Solicited Safety Set will be used for the analyses of solicited ARs and participants will be included in the vaccination group corresponding to what they actually received.

Safety Analysis: All safety analyses will be based on the Safety Set, except summaries of solicited ARs, which will be based on the Solicited Safety Set. All safety analyses will be performed by vaccination group. Participants will be included in the vaccination group corresponding to what vaccine they actually received.

Safety and reactogenicity will be assessed by clinical review of all relevant parameters, including solicited ARs (local and systemic ARs), unsolicited AEs (including any clinical safety laboratory abnormalities), treatment-related AEs, severe AEs, SAEs, MAAEs, AEs leading to withdrawal from study participation, AESIs, vital sign measurements, and physical examination findings.

The number and percentage of participants with any solicited local AR or solicited systemic AR during the 7-day follow-up period after the study injection will be summarized. A 2-sided 95% confidence interval (CI) using the Clopper-Pearson method will also be provided for the percentage of participants with any solicited AR.

The number and percentage of participants with unsolicited AEs, treatment-related AEs, severe AEs, SAEs, AESIs, MAAEs, and AEs leading to withdrawal from study participation will be summarized. Unsolicited AEs will be coded according to the Medical Dictionary for Regulatory Activities (MedDRA) for AR terminology and presented by MedDRA system organ class and preferred term.

Solicited ARs will be coded according to the MedDRA for AR terminology. The toxicity grading scale for healthy adult and adolescent volunteers enrolled in preventive vaccine clinical trials will be used in this study with modification for rash, solicited ARs, and vital signs. Unsolicited AEs will be presented by MedDRA system organ class and preferred term.

The number of events of unsolicited AEs/SAEs, AESIs, MAAEs, and AEs leading to withdrawal will be reported in summary tables accordingly. For all other safety parameters, descriptive summary statistics will be provided.

Assessment of safety laboratory tests will be done for this study. The number and percentage of participants who have chemistry and hematology results below or above the normal laboratory ranges will be tabulated by time point. For treatment-emergent safety laboratory test results, the raw values and change from baseline values will be summarized by vaccination group and visit at each time point.

Immunogenicity Analysis: The analyses of immunogenicity will be based on the Per-Protocol (PP) Set. If the number of participants in the full analysis set (FAS) and PP Set differ (defined as the difference divided by the total number of participants in the PP Set) by more than 10%, supportive analyses of immunogenicity may be conducted using the FAS.

For the immunogenicity endpoints, geometric mean of specific antibody titers with corresponding 95% CI at each time point and geometric mean fold rise (GMFR) of specific antibody titers with corresponding 95% CI at each postbaseline time point over preinjection baseline at Day 1 will be provided by treatment arm. Descriptive summary statistics including median, minimum, and maximum will also be provided.

For summarizations of geometric mean titers, antibody titers reported as below lower limit of quantification (LLOQ) will be replaced by 0.5 × LLOQ. Values that are greater than the upper limit of quantification (ULOQ) will be converted to the ULOQ.

For HA, rate of seroconversion is defined as the proportion of participants with either a prevaccination HI titer < 1:10 and a postvaccination HI titer ≥ 1:40 or a prevaccination HI titer ≥ 1:10 and a minimum 4-fold rise in postvaccination HI antibody titer. For NA, an endpoint of interest is the percentage of participants with a change in the Day 29 titer of at least 2-/3-/4-fold rise, defined as ≥ 2-/3-/4-fold of the LLOQ if the Day 1 titer is < LLOQ; or ≥ 2-/3-/4-fold of the Day 1 titer if the Day 1 titer is ≥ LLOQ.

Seroconversion rate from baseline will be provided with a 2-sided 95% CI using the Clopper-Pearson method at each postbaseline time point. For NA, the number and percentage of participants with a ≥ 2, ≥ 3, and ≥ 4-fold rise of serum titers from baseline will be provided with 2-sided 95% CI using the Clopper-Pearson method at each postbaseline time point.

Further details will be described in the Statistical Analysis Plan.

Sample Size Justification: There is no hypothesis testing in this study. The number of proposed participants is considered sufficient to provide a descriptive summary of the safety and immunogenicity of different dose levels of mRNA-1020 or mRNA-1030.

A total of approximately 560 participants will be randomly assigned to receive mRNA-1020, mRNA-1030, mRNA-1010, or Flublok. Among those 560 participants, 70 participants will be randomized into each vaccination group. With 70 participants in each group receiving the IP, there is an approximately 76% probability to observe at least 1 participant with an AE if the true incidence of the AE is 2%; if the true incidence rate is 4%, then the probability to observe an AE is approximately 94%.

Study Analyses:

One interim analysis (IA) is planned in this study. It will be performed after participants have completed the Day 29 Visit. All data relevant to the IA through the Day 29 Visit will be cleaned (ie, data that are as clean as possible).

The IA will be performed by a separate team of unblinded programmers and statisticians. Except for a limited number of Sponsor and CRO personnel who will be unblinded to perform the IA, the study site staff, Investigators, study monitors, and participants will remain blinded until after the final database lock for final analysis.

The final analysis of safety, reactogenicity, and immunogenicity will be performed after all participants have completed all planned study procedures. The results of this analysis will be presented in a final clinical study report, including individual listings.

1.2. Schema

Figure 1: Study Schema

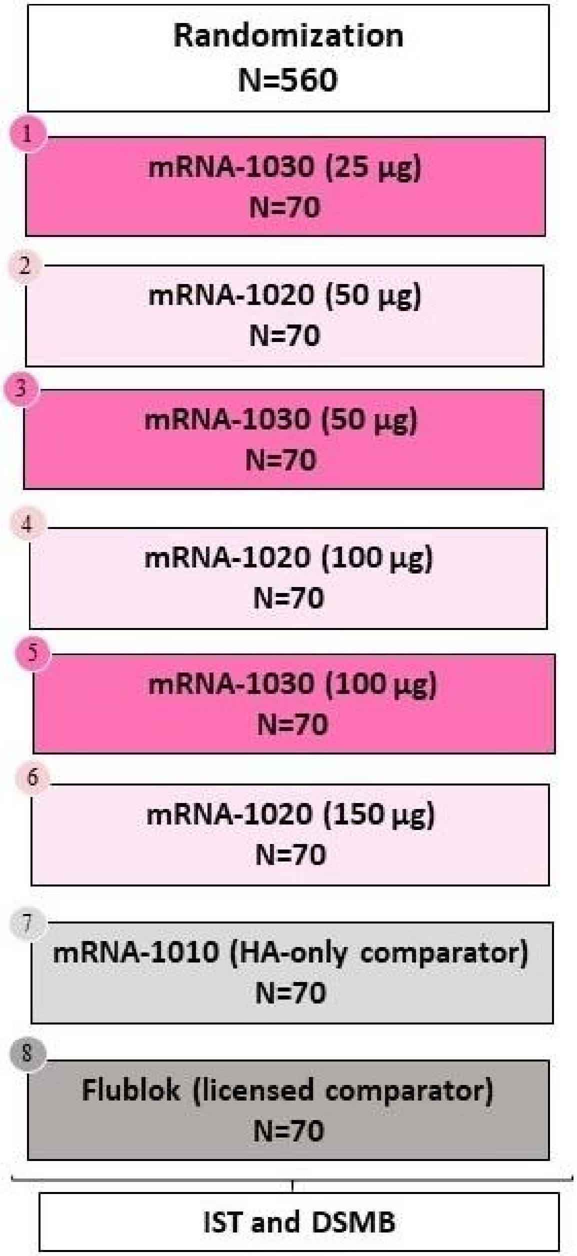

Abbreviations: DSMB = data safety monitoring board; HA = hemagglutinin; IST = internal safety team; V = visit.

1.3. Schedule of Events

Table 1: Schedule of Events

**Visit Number**		1	2	3	4	5, 6, 7, and 8	9	USV
**Type of Visit**	C	C	C	C	C	SC	C	C
**Month Time Point**					M1	M2-M5	M6	Up to M6
**Visit Day**	Screening[a]	D1[a] (Baseline)	D4	D8	D29	D57, D91, D121, D151	D181/EoS	N/A
**Window Allowance (Days)**	-28	-	-2	-2 or +1	-7 to +2	±5	±14	N/A
Informed consent form, demographics, medical history[b]	X							
Inclusion/exclusion criteria	X	X						
Blood collection for safety laboratory samples[c]	X			X				
Full physical examination[d]	X							
Symptom-directed physical examination[d,e]		X		X	X		X	X
Injection site and axillary lymph node assessment (injected arm)		X						
Vital sign measurements[e]	X	X						
ECG[f]		X						
Pregnancy testing[g]	X	X						
Randomization		X						
Study vaccination (including 60-minute postdose observation period)		X[h]						
Blood collection for humoral immunogenicity[i]		X		X	X		X	
Blood collection for cellular immunogenicity[i]		X		X	X			
Optional blood collection for genomics		X						
Optional blood collection for transcriptomics		X		X	X			
Blood sample for biomarker analysis[j]			X					
Nasopharyngeal swab for virus detection[k]		X						X
eDiary activation for recording solicited ARs (7 days)[l]		X						

**Visit Number**		1	2	3	4	5, 6, 7, and 8	9	USV
**Type of Visit**	C	C	C	C	C	SC	C	C
**Month Time Point**					M1	M2-M5	M6	Up to M6
**Visit Day**	Screening[a]	D1[a] (Baseline)	D4	D8	D29	D57, D91, D121, D151	D181/EoS	N/A
**Window Allowance (Days)**	-28	-	-2	-2 or +1	-7 to +2	±5	±14	N/A
Review of eDiary				X				
Follow-up safety call[m]						X		
Recording of all unsolicited AEs		X		X	X			
Recording of SAEs, AESIs, MAAEs, and AEs leading to study withdrawal		X		X	X	X	X	X
Recording of concomitant medications and nonstudy vaccinations[n]	X	X		X	X	X	X	X
Study completion							X	

Abbreviations: AE = adverse event; AESI= adverse event of special interest; ALT = alanine aminotransferase; AR = adverse reaction; AST = aspartate aminotransferase; C = clinic visit; COVID-19 = coronavirus disease 2019; D = day; eCRF = electronic case report form; ECG = electrocardiogram; ED = eDiary review; eDiary = electronic diary; EoS = end of study; IM = intramuscular; M = month; MAAE = medically attended adverse event; N/A = not applicable; NP = nasopharyngeal; PCR = polymerase chain reaction; SAE = serious adverse event; SARS-CoV-2 = severe acute respiratory syndrome coronavirus 2; SC = safety (phone) call; USV = unscheduled visit; WBC = while blood cell.

a Screening and Day 1 will NOT be performed on the same day. Additionally, the Screening Visit may be performed over multiple visits if within the 28-day screening window.

b Verbal confirmation of medical history is acceptable.

c Safety laboratory tests: Total WBC count, hemoglobin, platelets, ALT, AST, creatinine, alkaline phosphatase, and total bilirubin.

d Physical examination: A full physical examination, including height and weight, will be performed at screening; symptom-directed physical examinations may be performed at all other scheduled time points. Interim physical examinations will be performed at the discretion of the Investigator. Any clinically significant finding identified by a healthcare professional during study visits should be reported as an MAAE.

e Vital sign measurements: Systolic and diastolic blood pressure, heart rate, respiratory rate, and body temperature. The preferred route of temperature assessment is oral. Participants must not eat or drink anything hot or cold within 10 minutes before oral temperature is taken. Participants will be seated for at least 5 minutes before all measurements are taken. Vital signs will be collected on the day of vaccination, once before vaccination and approximately 1 hour after vaccination. Vital signs may be collected at other study site visits in conjunction with a symptom-directed physical examination. If any of the vital sign measurements meet the toxicity grading criteria for clinical abnormalities of Grade 3 or greater, the abnormal value and grade will be documented in the AE section of the eCRF (unless there is another known cause of the abnormality that would result in an AE classification). The Investigator will continue to monitor the participant with additional assessments until the vital sign value has reached the reference range, returns to the vital sign value at baseline, is considered stable, or until the Investigator determines that follow-up is no longer medically necessary.

f A 12-lead ECG will be obtained after 10 minutes of supine rest at Visit 1/Day 1 prior to vaccination (see Section 8.1.6).

g A point-of-care urine pregnancy test will be performed at the Screening Visit and before the vaccine dose on Day 1. At the discretion of the Investigator, a pregnancy test either via blood or point-of-care urine can be performed at any time. The follicle-stimulating hormone level may be measured at the Screening Visit, as necessary, and at the discretion of the Investigator, to confirm menopausal status.

h All participants will be randomized to receive a single IM injection.

[i] Samples for humoral immunogenicity and cellular immunogenicity must be collected prior to receipt of vaccination on Day 1. Cellular immunogenicity samples will be collected from approximately 240 participants (30 participants per vaccination group) and assessed in a subset of participants.

[j] Biomarker plasma and biomarker serum samples will be stored for potential future biomarker assessment.

[k] An NP swab specimen for pathogens, including influenza and other respiratory pathogens (eg, SARS-CoV-2) will be collected prior to study injection on Day 1 to assess for pre- or asymptomatic infection prior to vaccination. An NP swab will also be collected through study completion if any signs or symptoms suggesting COVID-19 or other causes of upper or lower respiratory infection occur. For signs or symptoms during the study, a participant will be instructed to contact the study site to have an NP swab collected for testing. The NP swab may be collected as part of a home visit in lieu of a study site visit. In the event that NP swabs during ILI cannot be collected, any available influenza and/or SARS-CoV-2 testing results performed outside of the study should be captured in the eCRF.

[l] The eDiary entries will be recorded by the participant at approximately 1 hour after vaccination while at the clinic with instruction provided by study staff. Study participants will continue to record in the eDiary each day after they leave the clinic, preferably in the evening and at the same time each day, on the day of vaccination and for 6 days following vaccination. If a solicited local or systemic AR starts more than 7 days after dosing, it should be captured on the AE page until no longer reported, not to exceed 28 days after vaccination. If a solicited local or systemic AR continues beyond 7 days after dosing, the participant should notify the site to provide an end date and close out the event. Adverse reactions beyond Day 7 should be reviewed by the study site staff either during the next scheduled telephone call or at the next study site visit.

[m] Trained study personnel will call all participants to collect information related to any SAE, AESIs, MAAEs, AEs leading to withdrawal from study, information on concomitant medications associated with those events, and any nonstudy vaccinations.

[n] All nonstudy vaccinations will be recorded through Day 181/EoS. All concomitant medications and nonstudy vaccinations will be recorded through 28 days after vaccination; all concomitant medications relevant to or for the treatment of an SAE, AESI, or MAAE will be recorded from Day 1 through Day 181/EoS.

TABLE OF CONTENTS

CLICAL STUDY PROTOCOL 1
PROTOCOL APPROVAL – SPONSOR SIGNATORY 2
DECLARATION OF INVESTIGATOR 3
PROTOCOL AMENDMENT SUMMARY OF CHANGES 4
1. PROTOCOL SUMMARY 6
1.1. Synopsis 6
1.2. Schema 18
1.3. Schedule of Events 19
TABLE OF CONTENTS 22
LIST OF TABLES 26
LIST OF FIGURES 26
LIST OF ABBREVIATIONS 27
2. INTRODUCTION 29
2.1. Study Rationale 29
2.2. Background and Overview 29
2.2.1. mRNA-1020 and mRNA-1030 30
2.2.2. Nonclinical Studies 30
2.2.3. Clinical Studies 32
2.3. Benefit/Risk Assessment 32
2.3.1. Known Potential Benefits 32
2.3.2. Risks from Study Participation and Their Mitigation 33
2.3.3. Overall Benefit/Risk Conclusion 34
3. OBJECTIVES AND ENDPOINTS 35
4. STUDY DESIGN 37
4.1. General Design 37
4.2. Scientific Rationale for Study Design 38
4.3. Choice of Vaccine Dose 39
4.4. End of Study Definition 40
5. STUDY POPULATION 41
5.1. Inclusion Criteria 41

5.2. Exclusion Criteria 41
5.3. Lifestyle Restrictions 43
5.4. Screen Failures....43
6. STUDY TREATMENT....44
6.1. Investigational Products Administered....44
6.2. Randomization....44
6.3. Preparation/Handling/Storage/Accountability....45
6.3.1. Preparation of Investigational Product 45
6.3.2. Investigational Product Administration....45
6.3.3. Investigational Product Delivery and Receipt 45
6.3.4. Investigational Product Packaging and Labeling 46
6.3.5. Investigational Product Storage....46
6.3.6. Investigational Product Accountability 47
6.3.7. Investigational Product Handling and Disposal 47
6.4. Investigational Product Compliance....47
6.5. Prior and Concomitant Medications 47
6.5.1. Prior Medications and Therapies....47
6.5.2. Concomitant Medications and Vaccinations 48
6.5.3. Concomitant Medications and Vaccines that May Lead to the Elimination of a Participant from Per-Protocol Analyses....49
6.6. Continued Access to Study Intervention After the End of the Study....49
7. DELAY OR DISCONTINUATION OF STUDY INTERVENTION AND PARTICIPANT DISCONTINUATION/WITHDRAWAL 50
7.1. Pause Rules....50
7.1.1. Pause Rules Based on the Occurrence of a Single Event and Adjudicated by the Data Safety Monitoring Board....50
7.2. Criteria for Delay or Withholding of Study Vaccination 51
7.3. Participant Discontinuation/Withdrawal from the Study 52
7.4. Lost to Follow-up 54
8. STUDY ASSESSMENTS AND PROCEDURES....55
8.1. Safety Assessments and Procedures 56
8.1.1. Use of Electronic Diaries....56
8.1.2. Safety Phone Calls....57

8.1.3. Safety Laboratory Assessments 58
8.1.4. Vital Sign Measurements 58
8.1.5. Physical Examinations 59
8.1.6. Electrocardiogram 59
8.1.7. Assessments for Respiratory Viral Infections 59
8.2. Immunogenicity Assessments 60
8.3. Efficacy Assessments 61
8.4. Safety Definitions and Related Procedures 61
8.4.1. Adverse Event 61
8.4.2. Serious Adverse Events 62
8.4.3. Solicited Adverse Reactions 63
8.4.4. Medically Attended Adverse Events 65
8.4.5. Influenza-Like Illness 66
8.4.6. Adverse Events of Special Interest 66
8.4.7. Recording and Follow-up of Pregnancy 67
8.4.8. Eliciting and Documenting Adverse Events 68
8.4.9. Assessment of Intensity 68
8.4.10. Assessment of Causality 69
8.4.11. Reporting Adverse Events 69
8.4.12. Reporting Serious Adverse Events 70
8.4.13. Reporting of Adverse Events of Special Interest 71
8.4.14. Time Period and Frequency for Collecting Adverse Event and Serious Adverse Event Information 71
8.4.15. Method of Detecting Adverse Events and Serious Adverse Events 72
8.4.16. Follow-up of Adverse Events, Serious Adverse Events, and Adverse Events of Special Interest 72
8.4.17. Regulatory Reporting Requirements for Serious Adverse Events 72
8.5. Safety Monitoring 73
8.6. Treatment of Overdose 73
8.7. Pharmacokinetics 73
8.8. Pharmacodynamics 73
8.9. Biomarkers 73
8.10. Health Economics 73

9. STATISTICAL ANALYSIS PLAN ... 74
9.1. Blinding and Responsibility for Analyses ... 74
9.1.1. Breaking the Blind ... 75
9.2. Statistical Hypotheses ... 75
9.3. Sample Size Determination ... 75
9.4. Analysis Sets ... 76
9.5. Statistical Methods ... 76
9.5.1. Baseline Characteristics and Demographics ... 76
9.5.2. Safety Analyses ... 77
9.5.3. Immunogenicity Analyses ... 78
9.5.4. Exploratory Analyses ... 79
9.5.5. Subgroup Analyses ... 79
9.6. Study Analyses ... 79
10. REFERENCES ... 80
11. SUPPORTING DOCUMENTATION AND OPERATIONAL CONSIDERATIONS ... 84
11.1. APPENDIX 1: Study Governance Considerations ... 85
11.1.1. Regulatory and Ethical Considerations ... 85
11.1.2. Study Monitoring ... 85
11.1.3. Audits and Inspections ... 86
11.1.4. Financial Disclosure ... 87
11.1.5. Recruitment Procedures ... 87
11.1.6. Informed Consent Process ... 87
11.1.7. Protocol Amendments ... 88
11.1.8. Protocol Deviations ... 89
11.1.9. Data Protection ... 89
11.1.10. Sample Retention and Future Biomedical Research ... 90
11.1.11. Safety Oversight ... 90
11.1.12. Dissemination of Clinical Study Data ... 91
11.1.13. Data Quality Assurance and Quality Control ... 91
11.1.14. Data Collection and Management ... 93
11.1.15. Source Documents ... 93
11.1.16. Retention of Records ... 93

11.1.17. Study and Site Closure....94
11.1.18. Publication Policy....94
11.2. APPENDIX 2: Contraceptive Guidance....96
11.3. APPENDIX 3: Adverse Events of Special Interest Terms....98
11.4. APPENDIX 4: CDC Working Case Definition of Pericarditis, Myocarditis, and Myopericarditis Occurring After Receipt of COVID-19 mRNA Vaccines....99

LIST OF TABLES

Table 1: Schedule of Events....19
Table 2: Study Objectives and Endpoints....35
Table 3: Mass Ratios for Dose Levels....38
Table 4: Study Arms and Dose Levels....38
Table 5: Pause Rule Criteria, Events, and Thresholds - Single Event....50
Table 6: Pause Rule Criteria, Events, and Thresholds – Proportion of Participants....51
Table 7: Solicited Adverse Reactions and Grades....64
Table 8: Analysis Sets....76
Table 9: Analysis Strategy for Safety Parameters....78
Table 10: Adverse Events of Special Interest....98
Table 11: Case Definitions of Probable and Confirmed Myocarditis, Pericarditis, and Myopericarditis....99

LIST OF FIGURES

Figure 1: Study Schema....18

LIST OF ABBREVIATIONS

The following abbreviations and terms are used in this study protocol.

Abbreviation or Specialist Term	Definition
AE	adverse event
AESI	adverse events of special interest
CDC	United States Centers for Disease Control and Prevention
CFR	Code of Federal Regulations
CI	confidence interval
COVID-19	coronavirus disease 2019
CRO	contract research organization
DHHS	Department of Health and Human Services
DSMB	data safety monitoring board
ECG or EKG	electrocardiogram
eCRF	electronic case report form
EDC	electronic data capture
EoS	end of study
FAS	Full Analysis Set
FDA	Food and Drug Administration
FSH	follicle-stimulating hormone
GCP	Good Clinical Practice
GMFR	geometric mean fold rise
GMT	geometric mean titer
HA	hemagglutinin
HAI	hemagglutination inhibition
HCP	healthcare practitioner
IA	interim analysis
IB	investigator’s brochure
ICF	informed consent form
ICH	International Council for Harmonisation

Abbreviation or Specialist Term	Definition
ILI	influenza-like illness
IM	intramuscular(ly)
IND	investigational new drug application
IRB	institutional review board
IRT	interactive response technology
IST	internal safety team
LLOQ	lower limit of quantification
LNP	lipid nanoparticle
LTFU	lost to follow-up
MAAE	medically attended adverse event
MedDRA	Medical Dictionary for Regulatory Activities
mRNA	messenger ribonucleic acid
MN	microneutralization
NA	neuraminidase
NAI	neuraminidase inhibition
NP	nasopharyngeal
PCR	polymerase chain reaction
PP	per-protocol
QA	quality assurance
RT-PCR	reverse transcription polymerase chain reaction
SAE	serious adverse event
SAP	statistical analysis plan
SARS-CoV-2	severe acute respiratory syndrome coronavirus 2
SoE	schedule of events
ULOQ	upper limit of quantification
USP	United States Pharmacopeia
WBC	white blood cells

2. INTRODUCTION

2.1. Study Rationale

Seasonal influenza viruses are estimated by the World Health Organization (WHO) to cause 3 to 5 million cases of severe illness and up to 650,000 deaths each year resulting in a severe challenge to public health (WHO 2018). Influenza epidemics occur each year and follow a seasonal circulation pattern with increased cases during the winter months in the Northern and Southern Hemispheres, respectively (Riedel et al 2019). Since influenza viruses continuously change through a process termed antigenic drift, the circulating viruses are actively monitored by a worldwide monitoring network coordinated by the WHO (Monto 2018). Based on the observed circulation patterns and antigenic changes, an expert panel recommends influenza virus strains to be used for vaccine manufacturing twice per year (once for the Northern Hemisphere [NH] and once for the Southern Hemisphere [SH]). Influenza A and influenza B viruses are the most relevant influenza viruses for human infection. Therefore, current vaccine recommendations include 1 influenza A H1N1 strain, 1 influenza A H3N2 strain, and 2 influenza B strains (covering the B/Victoria and B/Yamagata lineages).

Currently licensed seasonal influenza virus vaccines rarely exceed 60% overall effectiveness and are poorly effective during years when the circulating viruses do not match the strains selected for the vaccine antigens (Centers for Disease Control and Prevention [CDC] 2020a). Influenza vaccines based on messenger RNA (mRNA)-technology could provide several benefits compared to current vaccines, including the ability to respond to strain changes more quickly, avoidance of mutations that may be acquired during vaccine production in eggs or cell culture, and stronger immune responses as well as improved protection in older adults (Rockman et al 2020). The Sponsor is planning to test 2 next-generation development candidates (mRNA-1020 and mRNA-1030) in a Phase 1/2 study, which will identify a candidate to take forward into the pivotal study(ies). A group receiving the Sponsor's first-generation development candidate mRNA-1010, as well as a group receiving a licensed enhanced seasonal influenza vaccine (eg, Flublok), will be included as control groups. The vaccines will be administered as a single dose and will aim to elicit protection from all seasonal influenza viruses covered by the vaccine.

2.2. Background and Overview

ModernaTX, Inc. (the Sponsor) has developed a rapid-response, proprietary vaccine platform based on an mRNA delivery system. The platform is based on the principle and observations that cells in vivo can take up mRNA, translate it, and then express protein viral antigen(s) on the cell surface. The delivered mRNA does not enter the cellular nucleus or interact with the genome, is non-replicating, and is expressed transiently.

Two mRNA vaccines against the severe acute respiratory syndrome coronavirus 2 (SARS-CoV-2), including Pfizer Inc.'s BNT162b2 (Polack et al 2020) and the Sponsor's mRNA-1273 (NCT04283461, NCT04405076, and NCT04470427), have been approved by the United States (US) Food and Drug Administration (FDA).

2.2.1. mRNA-1020 and mRNA-1030

The Sponsor is using its mRNA-based platform to develop a novel lipid nanoparticle (LNP)-encapsulated, mRNA-based seasonal vaccine against disease caused by influenza virus types A and B. All 3 proposed development candidates (mRNA-1010, mRNA-1020, and mRNA-1030) contain mRNAs encoding for the hemagglutinins (HAs) of the 4 strains recommended by the WHO for cell- or recombinant-based vaccines, but the two next-generation candidates (mRNA-1020 and mRNA-1030) are differentiated by the inclusion of mRNAs encoding for the neuraminidases (NAs) of the respective viruses at different HA:NA mass ratios (1:1 and 3:1, for mRNA-1020 and mRNA-1030, respectively). Equal amounts of mRNAs encoding for each of the 4 different strains will be used for both the HA and NA components.

The Sponsor is conducting a Phase 1/2 study of next-generation seasonal influenza mRNA vaccines to establish preliminary safety and immunogenicity data to support the initiation of the pivotal study(ies) for the lead candidate. The design of the Phase 1/2 study includes immunogenicity objectives for both the HA and NA components that represent surrogate markers for protection against influenza illness. The rationale for this approach is based on the established precedent of using HA-based immunologic surrogates for clinical assessment and licensure of influenza vaccines (Department of Health and Human Services [DHHS] 2007a, Dunning et al 2016, and European Medicines Agency [EMA] 2016) and the available literature indicating that NA-based immune responses contribute to protection against influenza disease in clinical studies and animal models of influenza virus immunogenicity and challenge (Dunning et al 2016, Memoli et al 2016, Murphy et al 1972, and Weiss et al 2020).

2.2.2. Nonclinical Studies

Using preclinical material, a mouse study (MOD-4218) was performed to evaluate the immunogenicity of multivalent influenza vaccines containing mRNAs encoding the 4 HA and 4 NA of the seasonal influenza strains recommended by the WHO. The goal was to compare the immunogenicity of different ratios of HA:NA, including a 1:1 HA:NA ratio (mRNA-1020) and 3:1 HA:NA ratio (mRNA-1030). The secondary goal of this study was to compare different seasonal vaccine compositions (NH 2020/2021 [NH20/21] composition versus SH 2021 [SH2021] composition). Mice were immunized via intramuscular (IM) injection on a prime/boost schedule with a 3-week interval and antibody responses were measured by enzyme-linked immunosorbent assay at Day 21 and Day 36, and by hemagglutination inhibition (HAI)

and neuraminidase inhibition (NAI) at Day 36. A prime/boost regimen was chosen since immunogenicity of seasonal influenza vaccines is often suboptimal in naïve hosts. However, mRNA-1020 and mRNA-1030 are envisioned as a single dose annual vaccine in human adults similar to currently licensed influenza vaccines. Serological analyses demonstrated potent immune responses against all antigens (HAs and NAs) after one dose of mRNA-1020 and mRNA-1030 vaccines, that was further boosted after the second dose. Robust HAI as well as NAI antibody titers were observed at Day 36 following mRNA-1020 and mRNA-1030 immunization. Finally, both the NH20/21 and SH21 mRNA vaccine compositions were immunogenic in mice as demonstrated in this study.

An additional mouse study (MOD-4485) was performed to evaluate the immunogenicity of mRNA-1020 and mRNA-1030 that code for the antigens of the NH21/22 vaccine composition (influenza strains used for the mRNA-1020-P101 study). Serological analyses demonstrated good immune responses against all antigens after 2 doses of mRNA-1020 or mRNA-1030 vaccines of the NH21/22, suggesting that the mRNA platform can readily support seasonal influenza virus vaccine strain changes.

In addition to the mouse immunogenicity studies, mouse and ferret influenza virus challenge studies were performed. Mice and ferrets were immunized with mRNA-1020 or mRNA-1030 of the SH21 composition (using non-Good Manufacturing Practice co-formulated material) followed by a viral challenge. Control animals were immunized with phosphate buffered saline or a commercially available MF59-adjuvanted influenza vaccine (FLUAD®, NH20/21 composition). Ferrets were vaccinated via IM injection on a prime/boost schedule with a 3-week interval; antibody responses were measured at Day 21 and Day 42, followed by H1N1 challenge on Day 42. Mice received a single dose via IM injection and antibody responses were measured at Day 21 and groups of animals were subsequently challenged with either an (H1N1)pdm09-like or H3N2 challenge virus. In both animal models, serological analyses demonstrated that HAI antibody titers were detectable against the 4 vaccine strains after the first immunization with either mRNA-1020 or mRNA-1030 and in the case of ferrets, titers were further boosted by the second immunization. In addition, the HAI antibody titers induced by mRNA-1020 or mRNA-1030 were as robust as the titers induced by the adjuvanted comparator influenza vaccine (FLUAD). Upon live virus challenge, ferrets that were vaccinated with mRNA-1020 or mRNA-1030 had lower detectable viral loads in the lungs compared to the placebo group. No virus was detected in the lungs 4 days after challenge. Compared to FLUAD and placebo, mRNA-1020 and mRNA-1030 induced greater reduction in viral shedding in the upper respiratory tract with lower levels of viral loads in the nasal cavity (including the nasal turbinates) and the throat.

Upon challenge with a vaccine-mismatched H3N2 live virus, mice that were vaccinated with mRNA-1020 or mRNA-1030 exhibited less weight loss and fewer clinical symptoms compared to mice that received FLUAD or mice in the control group. Upon (H1N1)pdm09-like virus challenge, mice that received mRNA-1020, mRNA-1030, or FLUAD were fully protected against morbidity and showed no scorable clinical symptoms.

In conclusion, mRNA-1020 and mRNA-1030 vaccines are immunogenic, induce strong antibody responses in mice and ferrets, and confer protective efficacy against live virus challenge.

A detailed review of the nonclinical experience with mRNA-1020 and mRNA-1030 vaccines is provided in the investigator's brochure (IB). Additionally, the nonclinical experience with mRNA-1010 is provided in the mRNA-1010 IB.

2.2.3. Clinical Studies

No clinical studies with mRNA-1020 or mRNA-1030 have been performed to date. The Sponsor's Phase 1/2 mRNA-1010-P101 study (NCT04956575) is currently ongoing to provide initial first-in-human safety and immunogenicity data on 3 dose levels of mRNA-1010 encoding strains (50, 100, or 200 µg total mRNA) recommended by the WHO for the 2021 SH. An amendment to mRNA-1010-P101 includes a Phase 2 NH portion to the study to increase the size of the safety database for the 50 and 100 µg dose levels and to test an additional 25 µg dose level of mRNA-1010 as well as a licensed seasonal influenza vaccine as an active comparator. Additionally, an amendment is planned to assess 6.25 µg and 12.5 µg dose levels of mRNA-1010 as well as 25 µg mRNA-1010 and an active comparator in the Phase 2 Extension portion of the study. The mRNA-1010 vaccine encoding strains recommended by the WHO for the 2021/2022 NH is used in the Phase 2 NH and Phase 2 Extension portions.

2.3. Benefit/Risk Assessment

2.3.1. Known Potential Benefits

The following benefits may accrue to participants:

- The mRNA-1020 and mRNA-1030 vaccines may be effective against seasonal influenza strains as recommended by the WHO for the 2021-2022 NH influenza season.
- Participants will have a baseline (Day 1) evaluation for respiratory pathogens, including influenza virus and SARS-CoV-2, and ongoing monitoring for influenza-like illness (ILI) and/or coronavirus disease 2019 (COVID-19) throughout the study.

- The study will contribute to the development of a potentially efficacious vaccine against seasonal influenza.

2.3.2. Risks from Study Participation and Their Mitigation

Adverse events (AEs) ranging from immediate mild allergic reactions (eg, urticaria) to systemic allergic reactions (eg, anaphylaxis) may occur following any vaccination. Systemic allergic reactions are very rare and are estimated to occur once per 450,000 vaccinations for vaccines that do not contain allergens such as gelatin or egg protein (Zent et al 2002). Since the authorization of the mRNA-1273 vaccine for COVID-19, the US CDC estimate of the rate of anaphylaxis based on reporting in the Vaccine Adverse Event Reporting System is approximately 2.5 cases/million doses administered (Shimabukuro et al 2021). As a precaution, all participants will remain under observation at the study site for at least 60 minutes after injection.

Vasovagal syncope (fainting) can occur before or after any vaccination, is usually triggered by pain or anxiety associated with the injection, and is not related to the substance injected. Therefore, it is important that standard precautions and procedures are followed to avoid injury from fainting.

Intramuscular injection with other mRNA vaccines manufactured by the Sponsor containing the proprietary SM-102 (heptadecan-9-yl 8-((2-hydroxyethyl)(6-oxo-6-(undecyloxy)hexyl)amino) octanoate) lipid formulation have commonly resulted in transient and self-limiting local inflammatory reactions. These typically included pain, erythema (redness), or swelling (hardness) at the injection site, which were mostly mild to moderate in severity and usually occurred within 24 hours of injection. Laboratory abnormalities (including increases in liver function tests) following injection have been observed in early phase clinical studies with similar mRNA-based vaccines. These abnormalities were without clinical symptoms or signs and returned toward baseline (Day 1) values over time.

In a completed Phase 3 study of mRNA-1273 vaccine for COVID-19 in 30,420 healthy adults, the most commonly reported local reactions included pain, swelling, and erythema at the injection site. Most of these reactions were Grade 1 or 2 in severity and resolved within 3 to 4 days of onset. The most commonly reported systemic reactions were headache, myalgia, arthralgia, fatigue, chills, and fever. In most cases, the reactions resolved spontaneously within several days (Baden et al 2021).

Similarly, safety results from Phase 1 studies conducted by the Sponsor on 2 mRNA vaccines containing the HA glycoproteins from the H10N8 and H7N9 avian influenza viruses were well tolerated, although the Sponsor's LNP/mRNA platform has since been updated (eg, SM-102 is used in mRNA-1010 and mRNA-1273; Feldman et al 2019). The Sponsor's Phase 1/2 study of mRNA-1010 is underway to evaluate safety and reactogenicity of mRNA-1010. No significant

safety concerns were identified during the interim analysis (IA) of available data through Day 29 of the study.

There have been very rare reports of myocarditis and pericarditis occurring after vaccination with COVID-19 mRNA vaccines. The majority of the cases have been reported in young males shortly after the second dose of the COVID-19 mRNA vaccine. These are typically mild cases and individuals tend to recover within a short time following standard treatment and rest. Investigators and study participants should be alert to the signs and symptoms of myocarditis and pericarditis (Gargano et al 2021).

2.3.3. Overall Benefit/Risk Conclusion

Participants will receive a single IM injection of a seasonal influenza vaccine: mRNA-1020, mRNA-1030, mRNA-1010, or Flublok. mRNA-1020, mRNA-1030, and mRNA-1010 are currently investigational vaccines and may or may not offer protection against seasonal influenza.

Serological data from all participants will be used to infer vaccine immunogenicity. Safety findings will be monitored and reviewed by the study team members to evaluate the safety status of all participants (Section 9.6). The internal safety team (IST) will also review and assess the safety data as described in Section 11.1.11.

Considering the nonclinical data for the mRNA-1010, mRNA-1020, and mRNA-1030 vaccines and the safety data for the other mRNA vaccines manufactured by the Sponsor to date that contain the proprietary SM-102 lipid formulation (eg, mRNA-1010), the Sponsor considers the potential benefits of participation to exceed the risks.

3. OBJECTIVES AND ENDPOINTS

The objectives which will be evaluated in this study and endpoints associated with each objective are provided in Table 2.

Table 2: Study Objectives and Endpoints

Objectives	Endpoints
**Primary**	
• To evaluate the safety and reactogenicity of mRNA-1020, mRNA-1030, and mRNA-1010	• Frequency and grade of each solicited local and systemic reactogenicity AR during a 7-day follow-up period post-vaccination<br>• Frequency and severity of any unsolicited AEs during the 28-day follow-up period post-vaccination<br>• Frequency of any SAEs, AESIs, MAAEs, and AEs leading to withdrawal from Day 1 through Day 181/EoS<br>• Safety laboratory abnormalities through 7 days post-vaccination
• To evaluate the humoral immunogenicity of mRNA-1020, mRNA-1030, and mRNA-1010 against vaccine-matched influenza A and B strains at Day 29	• GMT and GMFR, comparing Day 29 to Day 1 (baseline), and percentage of participants with seroconversion, defined as a Day 29 titer ≥ 1:40 if baseline is < 1:10 or a 4-fold or greater rise if baseline is ≥ 1:10 in anti-HA antibodies measured by HAI assay<br>• GMT and GMFR of anti-NA measured by NAI assay at Day 1 and Day 29 and percentage of participants with a change in the Day 29 titer of at least 2-/3-/4-fold rise, defined as ≥ 2-/3-/4-fold of the LLOQ if the Day 1 titer is < LLOQ; or ≥ 2-/3-/4-fold of the Day 1 titer if the Day 1 titer is ≥ LLOQ
**Secondary**	
• To evaluate the humoral immunogenicity of mRNA-1020, mRNA-1030, and mRNA-1010 against vaccine-matched influenza A and B strains at all evaluable humoral immunogenicity time points	• GMT and GMFR (compared to Day 1) of anti-HA or anti-NA antibodies as measured by HAI, NAI, and/or MN assays
**Exploratory (may be performed)**	
• To evaluate the humoral immunogenicity against vaccine-mismatched influenza A and B strains	• GMT and GMFR (compared to Day 1) of anti-HA or anti-NA antibodies as measured by HAI, NAI, or MN assays against vaccine-mismatched strains

Objectives	Endpoints
• To evaluate cellular immunogenicity in a subset of participants	• Frequency, magnitude, and phenotype of virus-specific T-cell and B-cell responses measured by flow cytometry or other methods, and to perform targeted repertoire analysis of T-cells and B-cells after vaccination
• To further characterize antibody responses, for example, Fc-mediated function, avidity, or epitope specificity	• Frequency, specificities, or other endpoints to be determined, for the further characterization of antibody responses
• To assess the occurrence of clinical influenza in study participants and characterize their immune response to infection and viral isolates	• Frequency of clinical influenza and immune responses
• Sample collection to perform passive transfer studies in preclinical animal models	• Transfer of human sera into mice with subsequent influenza virus challenge to observe protection from morbidity and mortality conferred by NA-specific antibodies

Abbreviations: AE = adverse event; AESI = adverse event of special interest; AR = adverse reaction; EoS = end of study; Fc = fragment crystallizable; GMFR = geometric mean fold rise; GMT = geometric mean titer; HA = hemagglutinin; HAI = hemagglutination inhibition; LLOQ = lower limit of quantification; MAAE = medically attended adverse event; MN = microneutralization; NA = neuraminidase; NAI = neuraminidase inhibition; SAE = serious adverse event.

4. STUDY DESIGN

4.1. General Design

The study will be a Phase 1/2, randomized, observer-blind, dose-ranging study to evaluate the safety, reactogenicity, and immunogenicity of mRNA-1020 (4 HA and 4 NA at 1:1 HA:NA mass ratio) and mRNA-1030 (4 HA and 4 NA at 3:1 HA:NA mass ratio) in healthy adults 18 to 75 years of age. Enrollment in study arms will proceed in parallel with randomization (Figure 1) of all dose levels of mRNA-1020 and mRNA-1030 (Study Arms 1 to 6), the mRNA-1010 Comparator Arm (Study Arm 7), and the Flublok Comparator Arm (Study Arm 8). The study will randomize approximately 70 healthy adult participants into each study arm with a balanced age group representation stratified approximately 1:1 into age groups of 18 to < 50 years of age and ≥ 50 to ≤ 75 years of age.

The vaccines to be tested contain mRNAs encoding for the surface glycoproteins of the strains recommended by the WHO for 2021/22 NH cell- or recombinant-based vaccines:

- A/Wisconsin/588/2019(H1N1)pdm09
- A/Cambodia/e0826360/2020(H3N2)
- B/Washington/02/2019 (B/Victoria lineage)
- B/Phuket/3073/2013 (B/Yamagata lineage)

Study visits will consist of a Screening Visit (up to 28 days before the Day 1 visit), Vaccination Visit at Day 1, and subsequent study visits on Day 4, Day 8, Day 29 (Month 1), and Day 181/end of study (EoS; Month 6), with up to 7 months of study participation (Table 1). Unscheduled visits for potential ILI symptoms will include testing with a multiplex respiratory infection panel (Biofire or similar).

Immunizations for the study are planned during the NH spring, when influenza infection rates are typically low or declining. Approximately 560 participants will be randomized to receive a single dose of either one of the 2 next-generation candidate vaccines (mRNA-1020 or mRNA-1030) at different dose levels, a single dose of the first-generation candidate vaccine (mRNA-1010), or Flublok, a licensed enhanced seasonal influenza vaccine comparator (8 study arms total). Three different dose levels (50, 100, and 150 µg total mRNA) of the 1:1 HA:NA mass ratio vaccine (mRNA-1020), 3 different dose levels (25, 50, and 100 µg total mRNA) of the 3:1 HA:NA mass ratio vaccine (mRNA-1030), and a single dose level of mRNA-1010 (50 µg total mRNA) will be assessed. A description of the mass ratios for the dose levels are provided in Table 3. The target number of participants in each arm and the randomization ratio can be found in Table 4.

Table 3: Mass Ratios for Dose Levels

Program	Valency	Description of Encoded Antigens	Mass Ratio[a]
mRNA-1010	4	Four HA antigens	25% each
mRNA-1020	8	Four HA antigens<br>Four NA antigens	12.5% each
mRNA-1030	8	Four HA antigens<br>Four NA antigens	18.9% each HA<br>6.3% each NA

[a] Individual mass ratios are rounded for reporting, and the total of reported values may not equal 100%.

Table 4: Study Arms and Dose Levels

Vaccination Group	Group Name	Antigen(s)	Total Dose	N (total)	Age 18-49	Age 50-75
1	**mRNA-1030**	HA+NA	**25 µg**	70	35	35
2	**mRNA-1020**	HA+NA	**50 µg**	70	35	35
3	**mRNA-1030**	HA+NA	**50 µg**	70	35	35
4	**mRNA-1020**	HA+NA	**100 µg**	70	35	35
5	**mRNA-1030**	HA+NA	**100 µg**	70	35	35
6	**mRNA-1020**	HA+NA	**150 µg**	70	35	35
7	**mRNA-1010** (HA only comparator)	HA only	**50 µg**	70	35	35
8	**Flublok** (active comparator)	HA only	**180 µg**	70	35	35
Total				560	280	280

Abbreviations: HA = hemagglutinin; mRNA = messenger RNA; NA = neuraminidase.

4.2. Scientific Rationale for Study Design

This study is designed as an observer-blind study. Participants in Vaccination Groups 1, 3, and 5 will receive mRNA-1030 vaccine and participants in Groups 2, 4, and 6 will receive mRNA-1020 vaccine. mRNA-1010 and Flublok will be used as comparators for descriptive comparisons of safety and immunogenicity.

All participants will provide nasopharyngeal (NP) swab samples before the injection on Day 1 for assessment of asymptomatic infection with respiratory pathogens, including influenza virus and SARS-CoV-2, as influenza or COVID-19 symptoms may confound reactogenicity assessments (CDC 2021a). Furthermore, if a participant experiences any signs or symptoms suggesting ILI and/or COVID-19, an additional NP swab sample will be collected to confirm the diagnosis via reverse transcription polymerase chain reaction (RT-PCR). Clinical information may be collected to evaluate the severity of the clinical case (CDC 2020b).

4.3. Choice of Vaccine Dose

No clinical studies of mRNA-1020 or mRNA-1030 have been conducted to date.

Previous studies of the Sponsor's mRNA/LNP SM-102 platform have assessed dose levels as high as 250 µg for mRNA-1273, a SARS-CoV-2 vaccine (Jackson et al 2020, NCT04283461), and mRNA-1893, a Zika vaccine (unpublished data, NCT04917861). Additionally, doses up to 300 µg of the multivalent mRNA-1647, a cytomegalovirus vaccine (unpublished data, NCT03382405), and the multivalent mRNA-1653, a combination vaccine against human metapneumovirus and parainfluenza virus type 3 (unpublished data, NCT03392389), have been tested in Phase 1 studies. The vaccines were generally well tolerated at these dose levels.

The Sponsor has previously evaluated the immunogenicity of 2 mRNA vaccines containing the HA glycoprotein from the H10N8 and H7N9 influenza viruses. Although differences in the mRNA/LNP platform and the administration schedule (2 doses were given 3 weeks apart) limit extrapolation of the safety data, these mRNA vaccines against H10N8 and H7N9 influenza viruses elicited robust humoral immune responses and were well tolerated up to dose levels of 100 µg (for each HA type; Feldman et al 2019).

The Sponsor's Phase 1/2 mRNA-1010 P101 study (NCT04956575) is currently ongoing. No significant safety concerns have been observed in the ongoing Phase 1/2 portion of mRNA-1010-P101 study upon review of safety data up to 29 days by the DSMB. In that study, 45 participants in each group received 50 µg, 100 µg, or 200 µg doses of mRNA-1010. The local and systemic reactogenicity were mostly mild to moderate in severity. There were no Grade 4 adverse reactions or SAEs assessed by the Investigator as related to the study vaccine. The 3 doses of mRNA-1010 elicited comparable humoral immunogenicity responses. In addition, the Phase 2 NH portion of mRNA-1010-P101 study has enrolled 498 adults in a Phase 2 NH cohort in 4 groups: 25 µg mRNA-1010 (n=151), 50 µg mRNA-1010 (n=147), 100 µg mRNA-1010 (n=147), and Afluria (n=53). The interim analysis of safety data through Day 29 demonstrated no significant safety concerns. The frequency and severity of the reports of solicited adverse reactions (ARs) in the mRNA-1010 groups were acceptable across all dose levels. Based on this data, the highest dose level for mRNA-1020 and mRNA-1030 (150 and 100 µg total dose, respectively) were selected.

This first-in-human clinical study of mRNA-1020 or mRNA-1030 vaccines will evaluate the immunogenicity of mRNA-1020 and mRNA-1030 at proposed total dose levels of 50, 100, 150 and 25, 50, 100 µg total mRNA, respectively, administered as a single IM injection in generally healthy adults ≥ 18 to ≤ 75 years of age.

4.4. End of Study Definition

A participant is considered to have completed the study if he or she has completed all phases of the study including last scheduled procedure(s) as shown in the schedule of events (SoE), Table 1.

The EoS is defined as completion of the last visit of the last participant in the study or last scheduled procedure(s) as shown in the SoE (Table 1) for the last participant in the study.

5. STUDY POPULATION

Prospective approval of protocol deviations to recruitment and enrollment criteria, also known as protocol waivers or exemptions, is not permitted.

5.1. Inclusion Criteria

Each participant must meet all of the following criteria to be enrolled in this study:

1. Adults 18 to 75 years of age at the time of consent (Screening Visit). Participants may be rescreened if they are not medically stable at the Screening Visit.
2. Investigator assessment that participant understands and is willing and physically able to comply with protocol-mandated follow-up, including all procedures.
3. Participant has provided written informed consent for participation in this study, including all evaluations and procedures as specified in this protocol.
4. Body mass index of 18 kg/m^2 to 35 kg/m^2 (inclusive) at the Screening Visit.
5. Female participants of nonchildbearing potential may be enrolled in the study. Nonchildbearing potential is defined as post-menopausal or permanently sterilized (Appendix 2 [Section 11.2]). A follicle-stimulating hormone (FSH) level should be measured at the discretion of the Investigator, if necessary, to confirm postmenopausal status.
6. Female participants of childbearing potential may be enrolled in the study if the participant fulfills all the following criteria:
    - Has a negative pregnancy test at the Screening Visit and on the day of vaccination (Day 1).
    - Has practiced adequate contraception or has abstained from all activities that could result in pregnancy for at least 28 days prior to Day 1 (Appendix 2 [Section 11.2]). Adequate female contraception is defined as consistent and correct use of an FDA approved contraceptive method in accordance with the product label.
    - Has agreed to continue adequate contraception through 90 days following vaccine administration.
    - Is not currently breastfeeding.

5.2. Exclusion Criteria

Participants meeting any of the following criteria will be excluded from the study:

1. Participant has had close contact to someone with SARS-CoV-2 infection or COVID-19 as defined by the CDC or has had a positive SARS-CoV-2 test in the past 10 days prior to the Screening Visit (CDC 2020c, CDC 2021b).
2. Clinical screening laboratory values (total white blood cell [WBC] count, hemoglobin, platelets, alanine aminotransferase, aspartate aminotransferase, creatinine, alkaline phosphatase, and total bilirubin) > Grade 1 in laboratory abnormality.

3. Participant is acutely ill or febrile (temperature ≥ 38.0°C/100.4°F) 72 hours prior to or at the Screening Visit or Day 1. Participants meeting this criterion may be rescheduled within the 28-day screening window and will retain their initially assigned participant number.
4. History of a diagnosis or condition that, in the judgment of the Investigator, is clinically unstable or may affect participant safety, assessment of safety endpoints, assessment of immune response, or adherence to study procedures. Clinically unstable is defined as a diagnosis or condition requiring significant changes in management or medication within the 60 days prior to screening and includes ongoing workup of an undiagnosed illness that could lead to a new diagnosis or condition.
5. History of myocarditis, pericarditis, or myopericarditis.
6. Reported history of congenital or acquired immunodeficiency, immunosuppressive condition, or immune-mediated disease.
7. Dermatologic conditions that could affect local solicited AR assessments (eg, tattoos, psoriasis patches affecting skin over the deltoid areas).
8. Reported history of anaphylaxis or severe hypersensitivity reaction after receipt of any components of mRNA- or cell-based influenza (eg, Flublok) vaccine.
9. Reported history of bleeding disorder that is considered a contraindication to IM injection or phlebotomy.
10. Any medical, psychiatric, or occupational condition, including reported history of substance abuse, that, in the opinion of the Investigator, might pose additional risk due to participation in the study or could interfere with the interpretation of study results. Asymptomatic conditions and conditions with no evidence of end organ involvement (eg, mild hypertension, dyslipidemia) are not exclusionary, provided that they are being appropriately managed and are clinically stable (ie, unlikely to result in symptomatic illness within the time course of this study). Illnesses or conditions may be exclusionary, even if otherwise stable, due to therapies used to treat them (eg, immune-modifying treatments), at the discretion of the Investigator.
11. Participant has received systemic immunosuppressants or immune-modifying drugs for > 14 days in total within 180 days prior to screening (for corticosteroids ≥ 10 mg/day of prednisone equivalent) or is anticipating the need for immunosuppressive treatment at any time during participation in the study.
12. Participant has received or plans to receive any licensed or authorized vaccine, including COVID-19 vaccines, ≤ 28 days prior to the study injection (Day 1) or plans to receive a licensed or authorized vaccine within 28 days after the study injection.
13. Participant has received a NH 2021-2022 seasonal influenza vaccine or any other influenza vaccine within 180 days prior to Day 1.
14. Participant tested positive for influenza by CDC-recommended testing methods within 180 days prior to Day 1 (CDC 2020d).

15. Participant has received systemic immunoglobulins or blood products within 90 days prior to the Screening Visit or plans to receive during the study.
16. Diagnosis of malignancy within previous 10 years (excluding nonmelanoma skin cancer).
17. Participant has donated ≥ 450 mL of blood products within 28 days prior to the Screening Visit or plans to donate blood products during the study.
18. Participated in an interventional clinical study within 28 days prior to the Screening Visit based on the medical history interview or plans to do so while participating in this study.
19. Participant is an immediate family member or household member of study personnel, study site staff, or Sponsor personnel.

5.3. Lifestyle Restrictions

Participants must not eat or drink anything hot or cold within 10 minutes before oral temperature is taken. Participants in the study should defer vaccination with licensed or authorized vaccines, including COVID-19 vaccines, until after completion of their Day 29 Visit, if a licensed or authorized vaccine is available and they choose to receive it.

5.4. Screen Failures

Screen failures are defined as participants who consent to participate in the clinical study but are not subsequently randomly assigned to treatment. A minimum set of screen failure information is required to ensure transparent reporting of screen failures to meet the Consolidated Standards of Reporting Trials publishing requirements and to respond to queries from regulatory authorities. Minimum information includes date of informed consent, demography, reason(s) for screen failure, and eligibility criteria.

6. STUDY TREATMENT

6.1. Investigational Products Administered

The term “investigational product” (IP) refers to mRNA-1020, mRNA-1030, mRNA-1010, and/or Flublok vaccines administered in this study.

The vaccines to be tested in the proposed Phase 1/2 study contain mRNAs encoding for the surface glycoproteins of the strains recommended by the WHO for 2021/22 NH cell- or recombinant-based vaccines:

- A/Wisconsin/588/2019(H1N1)pdm09
- A/Cambodia/e0826360/2020(H3N2)
- B/Washington/02/2019 (B/Victoria lineage)
- B/Phuket/3073/2013 (B/Yamagata lineage)

mRNA-1020, mRNA-1030, and mRNA-1010 IPs are LNP dispersions encoding the seasonal influenza vaccine antigens, HA and NA (mRNA-1020 and mRNA-1030), or HA only (mRNA-1010) from influenza strains A/Wisconsin/588/2019(H1N1)pdm09, A/Cambodia/e0826360/2020(H3N2), B/Washington/02/2019 (B/Victoria lineage), and B/Phuket/3073/2013 (B/Yamagata lineage). mRNA-1020 contains a 1:1 mass ratio of HA and NA; mRNA-1030 contains a 3:1 mass ratio of HA and NA, and mRNA-1010 contains HA only.

All mRNAs are formulated in LNPs composed of 4 lipids are provided as sterile liquids for injection with a white-to-off white dispersion in appearance. mRNA-1020 and mRNA-1030 IP are filled at a concentration of 0.4 mg/mL [redacted] mRNA-1010 IP is filled at a concentration of 0.4 mg/mL [redacted]

mRNA-1020, mRNA-1030, mRNA-1010, and Flublok will be administered as single IM injections (Table 4).

6.2. Randomization

The Sponsor’s Biostatistics Department or designee will generate the randomized allocation schedule(s) for vaccination group assignment. Overall, approximately 560 participants will be randomized in a 1:1:1:1:1:1:1:1 ratio to receive either mRNA-1020 50 µg, mRNA-1020 100 µg, mRNA-1020 150 µg, mRNA-1030 25 µg, mRNA-1030 50 µg, mRNA-1030 100 µg, mRNA-1010 50 µg, or Flublok 180 µg, with approximately 70 healthy adult participants randomly assigned to each vaccination group (Figure 1 and Table 4). Randomization will be

stratified by age (18 to < 50 years versus ≥ 50 to ≤ 75 years) and will be balanced across the 2 age groups within each vaccination group.

6.3. Preparation/Handling/Storage/Accountability

6.3.1. Preparation of Investigational Product

In the study, the IP will be prepared for each participant based on the vaccination group. All mRNA vaccine injections will have a volume of 0.5 mL. Flublok will be administered at a volume of 0.5 mL. The IP (mRNA-1020, mRNA-1030, mRNA-1010, and Flublok) preparation instructions are detailed in the Pharmacy Manual.

6.3.2. Investigational Product Administration

The IP (mRNA-1020, mRNA-1030, mRNA-1010, or Flublok) will be administered as a single IM injection into the deltoid muscle on Day 1. Preferably, the IP should be administered into the nondominant arm.

Participants will be monitored for a minimum of 60 minutes after administration of the IP. Assessments will include vital sign measurements and monitoring for local or systemic reactions as shown in the SoE (Table 1).

The study site will be appropriately staffed with individuals with basic cardiopulmonary resuscitation training/certification. Either onsite resuscitation equipment and personnel or appropriate protocols for the rapid transport of a participant to a resuscitation area or facility are required.

Further instructions for the preparation and administration of mRNA-1020, mRNA-1030, mRNA-1010, and Flublok are described in the Pharmacy Manual.

6.3.3. Investigational Product Delivery and Receipt

The Sponsor or designee is responsible for the following:

- Supplying the IP (mRNA-1020, mRNA-1030, mRNA-1010, and Flublok).
- Confirming the appropriate labeling of the IP for clinical study use, so that it complies with the US legal requirements.

The Investigator is responsible for acknowledging receipt of the IP by a designated staff member at the site, which includes the following:

- Confirming that the IP was received in good condition.
- Confirming that the temperature during shipment from the Sponsor to the Investigator's designated storage location was appropriate.

- Confirming that the Sponsor has authorized the IP for use.
- Ensuring the appropriate dose level of the IP is properly prepared using aseptic technique.

Further description of the IP and corresponding instructions for the receipt, storage, preparation, administration, accountability, and destruction are described in the Pharmacy Manual.

6.3.4. Investigational Product Packaging and Labeling

The Sponsor will provide the Investigator (via the study site pharmacy) with adequate quantities of the IP. Sterile mRNA-1020, mRNA-1030, and mRNA-1010 will be packaged in 2R glass vials with a 0.8-mL fill volume. The IP will have all required labeling per regulations and will be supplied to the pharmacy in an unblinded manner. The Sponsor or Sponsor's designee will supply the diluent for the mRNA vaccines (as applicable) as well as Flublok. The 0.9% Sodium Chloride Injection, United States Pharmacopeia (USP) bears a commercial label and does not contain study-specific identification.

All IPs will be packaged and labeled in accordance with the standard operating procedures of the Sponsor or of its designee, Code of Federal Regulations (CFR) Title 21 Good Manufacturing Practice guidelines, International Council for Harmonisation (ICH) Good Clinical Practice (GCP) guidelines, guidelines for Quality System Regulations, and applicable regulations.

6.3.5. Investigational Product Storage

mRNA-1020, mRNA-1030, and mRNA-1010 must be stored at -60°C to -90°C (-76°F to -130°F) in a secure area with limited access and must be protected from moisture and light until it is prepared for administration (Section 6.3.1). The freezer should have automated temperature recording and a 24-hour alert system in place that allows for rapid response in case of freezer malfunction. The freezer must be connected to a backup generator. In addition, for IP accountability, staff are required to keep a temperature log to establish a record of compliance with these storage conditions. The study site is responsible for reporting any IP that was not temperature controlled during shipment or storage. Such IP will be retained for inspection by the monitor and disposed of according to approved methods.

The active comparator Flublok should be stored in its original container and in accordance with the instructions in the Pharmacy Manual.

The 0.9% Sodium Chloride Injection USP should be stored at 20°C to 25°C (68°F to 77°F) in a restricted access area.

6.3.6. Investigational Product Accountability

The Investigator is responsible for ensuring that the IP accountability staff maintain accurate records in an IP accountability log of receipt of all IPs, site IP inventory, IP dispensing, IP injections, and return to the Sponsor or alternative disposition of used and unused IP vials.

A site monitor will review the inventory and accountability log during site visits and at completion of the study. Additional details are provided in the Pharmacy Manual.

6.3.7. Investigational Product Handling and Disposal

The designated unblinded clinical research associate will reconcile the clinical study material during the conduct and at the end of the study for compliance. Once fully reconciled at the site, the clinical study material can be destroyed at the investigational site or Sponsor-selected third party, as appropriate.

Clinical study material may be destroyed at the study site only if permitted by local regulations and authorized by the Sponsor. A document for destruction (ie, Certificate of Destruction) must be obtained and sent to the Sponsor or designee. For further direction refer to Pharmacy Manual.

6.4. Investigational Product Compliance

The IP will be administered at the study site under direct observation of medically qualified study staff, and IP administration will be appropriately recorded (date and time) in the electronic case report form (eCRF). Qualified staff will confirm that the participant has received the entire dose of the IP. If a participant does not receive the IP, the reason for the missed dose will be recorded. Data will be reconciled with site accountability records to assess compliance.

The study site staff are responsible for ensuring that participants comply with the allowed study visit windows. If a participant misses a visit, every effort should be made to contact the participant and complete a visit within the defined visit window specified in the SoE (Table 1). If a participant does not complete a visit within the time window, that visit will be classified as a missed visit and the participant will continue with subsequent scheduled study visits. All safety requirements of the missed visit will be captured and included in the subsequent visit.

6.5. Prior and Concomitant Medications

6.5.1. Prior Medications and Therapies

Information about prior medications (including any prescription or over-the-counter medications, vaccines, or blood products) taken by the participant within the 28 days before providing informed consent (or as designated in the inclusion/exclusion requirements) will be recorded in the participant's eCRF.

6.5.2. Concomitant Medications and Vaccinations

At each study visit, study site staff must question the participant regarding any medications taken and nonstudy vaccinations received by the participant and record the following information in the eCRF:

- All nonstudy vaccinations administered within the period starting 28 days before the IP injection and through Day 181/EoS.
- Any seasonal influenza vaccine administered from September 2021 through Day 181/EoS.
- Any authorized or investigational COVID-19 vaccine at any time before the IP injection and through Day 181/EoS.
- All concomitant medications taken through 28 days after the IP injection. Antipyretics and analgesics taken prophylactically (ie, taken in the absence of any symptoms in anticipation of an injection reaction) will be recorded as such.
- Systemic steroids (≥ 10 mg/day of prednisone or equivalent), immunosuppressants, immune-modifying drugs, immunoglobulins, and/or blood products administered at any time during the study period after the IP injection.
- Any concomitant medications relevant to or for the treatment of an SAE, adverse event of special interest (AESI; Section 8.4.6), a medically attended AE (MAAE; Section 8.4.4), or an AE leading to withdrawal from Day 1 through Day 181/EoS.
- The participant will be asked in the eDiary if they have taken any antipyretic or analgesic medication to treat or prevent fever or pain within 7 days after the study vaccination, including the day of injection. Reported antipyretic or analgesic medications should be recorded in the source document by the study site staff during the study visits after vaccination or via other participant interactions (eg, telephone calls).

If a participant takes a prohibited drug therapy, the Investigator and the contract research organization's (CRO's) medical monitor will make a joint decision about continuing or withholding the study vaccination of the participant based on the time the medication was administered, the drug's pharmacology and pharmacokinetics, and whether use of the medication will compromise the participant's safety or interpretation of the data. It is the Investigator's responsibility to ensure that details regarding the concomitant medications are adequately recorded in the eCRF.

All medication and interventions necessary for the appropriate care of the study participant should be administered and appropriately documented along with the AE for which the treatment was initiated.

6.5.3. Concomitant Medications and Vaccines that May Lead to the Elimination of a Participant from Per-Protocol Analyses

The use of the following concomitant medications and/or vaccines will not require withdrawal of the participant from the study but may determine a participant's evaluability in the Per-Protocol (PP) analysis (see Table 8 for definition of PP Set):

- Any investigational or nonregistered product (drug or vaccine) other than the study vaccine used during the study period.
- Immunosuppressants or other immune-modifying drugs administered chronically (ie, more than 14 days in total) during the study period. For corticosteroids, this will mean that ≥ 10 mg/day of prednisone or equivalent is not permitted. Inhaled, nasal, and topical steroids are allowed.
- Long-acting immune-modifying drugs administered at any time during the study period (eg, infliximab).
- An authorized or licensed vaccine administered within 28 days after the study vaccination (Section 5.2).
- Immunoglobulins and/or any blood products administered during the study period.

6.6. Continued Access to Study Intervention After the End of the Study

IP will not be available to participants after the EoS.

7. DELAY OR DISCONTINUATION OF STUDY INTERVENTION AND PARTICIPANT DISCONTINUATION/WITHDRAWAL

7.1. Pause Rules

Study pause rules will be continuously monitored during all periods of the study by the Investigators, IST, and DSMB; as warranted. If the Investigator, IST, or DSMB request that the study be paused due to a safety concern, further study vaccination in the affected vaccination group and at higher dose levels will be suspended, but all other planned procedures relating to safety, reactogenicity, and immunogenicity assessments will continue as described in the protocol. The Sponsor will notify the Center for Biologics Evaluation and Research within 48 hours in the event of a study pause.

7.1.1. Pause Rules Based on the Occurrence of a Single Event and Adjudicated by the Data Safety Monitoring Board

The occurrence of any of the events listed in Table 5, regardless of the vaccination group, will result in immediate suspension of dosing and enrollment in the vaccination group and at higher dose levels. An unscheduled DSMB will be convened to assess specific data concerns and to make recommendations.

Table 5: Pause Rule Criteria, Events, and Thresholds - Single Event

Pause Rule	Event	Number of Participants
1	Any SAE that cannot be reasonably attributed to a cause other than study vaccination	≥ 1
2	Any Grade 4 AE[a] that cannot be reasonably attributed to a cause other than study vaccination	≥ 1
3	Any systemic immediate hypersensitivity reaction within 60 minutes after the study vaccination	≥ 1
4	Suspected or confirmed myocarditis and/or pericarditis that cannot be reasonably attributed to a cause other than study vaccination	≥ 1

Abbreviations: AE = adverse event; AR = adverse reaction; FDA = Food and Drug Administration; SAE = serious adverse event; US = United States.

[a] Grade 4 AE includes any Grade 4 solicited local or systemic AR and any Grade 4 laboratory abnormality. Grading of laboratory parameters will be based on the US FDA Guidance for Industry "Toxicity grading scale for healthy adult and adolescent volunteers enrolled in preventive vaccine clinical trials" (DHHS 2007b).

7.1.1.1. Pause Rules Based on the Occurrence of Events in a Proportion of Participants

The occurrence of safety events that will pause study dosing based on defined threshold levels, which are aggregate incidences relative to the number of exposed participants within a vaccination group, are summarized in Table 6.

Table 6: Pause Rule Criteria, Events, and Thresholds – Proportion of Participants

Pause Rule	Event	Number or Percentage of Participants[a]
5	Any Grade 3 solicited local AR lasting more than 48 hours, within the 7-day postvaccination period	≥ 2[b] of the initial 12 participants or ≥ 20% of participants after the initial 12 participants have been enrolled
6	Any Grade 3 solicited systemic AR lasting more than 48 hours (24 hours for fever) that cannot be reasonably attributed to a cause other than vaccination, within the 7-day postvaccination period	≥ 2[b] of the initial 12 participants or ≥ 20% of participants after the initial 12 participants have been enrolled
7	Any severe unsolicited AE in a vaccination group that cannot be reasonably attributed to a cause other than vaccination<br>OR<br>Any Grade 3 laboratory abnormality[c] in a vaccination group that cannot be reasonably attributed to a cause other than vaccination	≥ 2[b] of the initial 12 participants or ≥ 20% of participants after the initial 12 participants have been enrolled

Abbreviations: AE = adverse event; AR = adverse reaction; FDA = Food and Drug Administration; MedDRA = Medical Dictionary for Regulatory Activities; US = United States.

[a] The rate of AEs and laboratory abnormalities will be computed based on the number of exposed participants who have provided safety data (ie, have completed a postdosing visit for assessment of safety).

[b] For the first 12 participants, the pause rule will be considered to be met if 2 of the first 12 participants experience the same solicited AR or the same MedDRA preferred term unsolicited AE or laboratory abnormality. The calculation for ≥ 20% of participants includes the total number of participants who have completed a postdosing visit in the denominator.

[c] Grading of laboratory parameters will be based on the US FDA Guidance for Industry "Toxicity grading scale for healthy adult and adolescent volunteers enrolled in preventive vaccine clinical trials" (DHHS 2007b).

If a pause is triggered in the study, each participant's study site visits will continue until EoS. If a pause affects a participant's vaccination visit, the window for that participant's vaccination visit will be suspended until the pause is lifted and vaccination can resume. Once the pause is lifted, vaccination should be reinstated as soon as possible.

If a participant is in the screening period for more than 28 days as the result of a pause, the participant may be rescreened for study eligibility (and will receive a new screening number) as long as the participant continues to provide consent to participate in the study.

7.2. Criteria for Delay or Withholding of Study Vaccination

Body temperature must be measured at the dosing visit before study vaccine administration. The following events constitute criteria for delay of injection, and if any of these events occur at the

time scheduled for dosing, the participant may be injected at a later date within the time window specified in the SoE (Table 1):

- Acute moderate or severe infection with or without fever at the time of dosing.
- Fever, defined as body temperature ≥ 38.0°C (100.4°F) at the time of dosing.
- Receipt of COVID-19 vaccination within 28 days of planned Day 1.

Participants with a minor illness without fever, as assessed by the Investigator, can be administered study vaccine. Participants with a fever ≥ 38.0°C (100.4°F) will be contacted within the time window acceptable for participation and reevaluated for eligibility. If the Investigator determines that the participant's health on the day of administration temporarily precludes injection, the visit should be rescheduled within the allowed interval for that visit.

The Investigator, in consultation with the Sponsor's medical monitor, should withhold the IP injection if the participant meets any of the following criteria:

- Becomes pregnant.
- Develops symptoms or conditions listed in the exclusion criteria.
- Experiences a clinically significant change in clinical laboratory test results, vital sign measurements, or general condition that, in the judgment of the Investigator, requires withholding of vaccine.

The reason(s) for withholding the injection will be recorded in the eCRF.

If a participant takes a prohibited drug therapy, the Investigator could delay the IP injection within the visit window or withhold the IP injection based on a joint decision of the Investigator and the CRO's medical monitor (Section 6.5.3).

7.3. Participant Discontinuation/Withdrawal from the Study

Participants who withdraw or are withdrawn from the study will not be replaced unless otherwise stated in the protocol.

A "withdrawal" from the study refers to a situation wherein a participant does not return for the final visit planned in the protocol.

Participants can withdraw consent and withdraw from the study at any time, for any reason, without prejudice to further treatment the participant may need to receive. The Investigator will request that the participant complete all study procedures pending at the time of withdrawal.

If a participant desires to withdraw from the study because of an AE, the Investigator will attempt to obtain agreement to follow-up with the participant until the event is considered resolved or stable and will then complete the EoS section of the eCRF.

Information related to the withdrawal will be documented in the eCRF. The Investigator will document whether the decision to withdraw a participant from the study was made by the participant or by the Investigator, as well as which of the following possible reasons was responsible for withdrawal:

- AE (specify)
- SAE (specify)
- Solicited AR or reactogenicity event (specify)
- Death
- Lost to follow-up (LTFU)
- Physician decision (specify)
- Pregnancy
- Protocol deviation
- Study terminated by Sponsor
- Withdrawal of consent by participant (specify)
- Other (specify)

Participants who are withdrawn from the study because of AEs (including SAEs, solicited ARs, or reactogenicity events) must be clearly distinguished from participants who are withdrawn for other reasons. Investigators will follow-up with participants who are withdrawn from the study as a result of an AE, SAE, solicited AR, or reactogenicity event until resolution of the event.

A participant withdrawing from the study may request destruction of any samples taken and not tested, and the Investigator must document this in the site study records.

If the participant withdraws consent for disclosure of future information, the Sponsor may retain and continue to use any data collected before such a withdrawal of consent (Section 11.1.6).

The Sponsor will continue to retain and use all research results that have already been collected for the study evaluation, unless the participant has requested destruction of these samples. All biological samples that have already been collected may be retained and analyzed at a later date (or as permitted by local regulations).

7.4. Lost to Follow-up

A participant will be considered LTFU if he or she repeatedly fails to return for scheduled visits without stating an intention to withdraw consent and is unable to be contacted by the study site. The following actions must be taken if a participant fails to return to the clinic for a required study visit:

- The site must attempt to contact the participant and reschedule the missed visit as soon as possible, counsel the participant on the importance of maintaining the assigned visit schedule, and ascertain whether the participant wishes to and/or should continue in the study.
- Before a participant is deemed LTFU, the Investigator or designee must make every effort to regain contact with the participant (where possible, 3 telephone calls and, if necessary, a certified letter to the participant's last known mailing address or local equivalent methods). These contact attempts (eg, dates of telephone calls and registered letters) should be documented in the participant's medical record.
- A participant who continues to be unreachable or continues to be noncompliant with study visits or procedures will be considered to have withdrawn from the study.

8. STUDY ASSESSMENTS AND PROCEDURES

Before performing any study procedures, all potential participants will sign an informed consent form (ICF; Section 11.1.6). Participants will undergo study procedures at the time points specified in the SoE (Table 1). A participant can also be seen for an unscheduled visit at any time during the study. Reasons for an unscheduled visit may include, but are not limited to, reactogenicity issues, symptoms of potential ILI and/or COVID-19, or new or ongoing AEs. The site also has the discretion to make reminder telephone calls or send text messages to inform the participant about visits, review eDiary requirements, or follow-up on ongoing or outstanding issues.

In accordance with "FDA Guidance on Conduct of Clinical Trials of Medical Products during COVID-19 Public Health Emergency" (DHHS 2020), Investigators may convert study site visits to home visits or telemedicine visits with the approval of the Sponsor. Such action should be taken to protect the safety and well-being of participants and study site staff or to comply with state or municipal mandates.

General considerations for study assessments and procedures include the following:

- Protocol waivers or exemptions are not allowed. The study procedures and their timing must be followed as presented in the SoE (Table 1). Adherence to the study design requirements is essential and required for study conduct.
- Immediate safety concerns should be discussed with the Sponsor immediately upon occurrence or awareness to determine if the participant should continue participation in the study.
- All screening evaluations must be completed and reviewed to confirm that potential participants meet all eligibility criteria. The Investigator will maintain a screening log to record details of all participants screened and to confirm eligibility or record reasons for screening failure, as applicable.
- The Screening Visit and Day 1 Visit cannot be completed on the same day. Additionally, the Screening Visit may be performed over multiple visits if within the 28-day screening window.

8.1. Safety Assessments and Procedures

Safety assessments will include monitoring and recording of the following for each participant, according to the SoE (Table 1):

- Solicited local and systemic ARs (Section 8.4.3) that occur during the 7 days following study vaccine administration (ie, the day of injection and 6 subsequent days). Solicited ARs will be recorded daily using eDiaries (Section 8.1.1).
- Unsolicited AEs observed or reported during the 28 days following study vaccine administration (ie, the day of injection and 27 subsequent days). Unsolicited AEs are defined in Section 8.4.1.
- AEs leading to discontinuation from study participation from Day 1 through Day 181/EoS or withdrawal from the study.
- SAEs, AESIs, and MAAEs from Day 1 through Day 181/EoS or withdrawal from the study.
- Results of safety laboratory tests (Section 8.1.3).
- Vital sign measurements (Section 8.1.4).
- Physical examination findings (Section 8.1.5).
- Baseline electrocardiograms (ECGs) results (Section 8.1.6).
- Assessments for causes of respiratory infections as needed from Day 1 through Day 181/EoS or through withdrawal from the study (Section 8.1.7).
- Pregnancy and accompanying outcomes in female participants reported after administration of study vaccination (Section 8.4.7).
- Concomitant medications and nonstudy vaccinations.

8.1.1. Use of Electronic Diaries

At the time of consent, the participants must confirm they are willing to complete an eDiary using either an application downloaded to their own device or using a device that is provided at the time of enrollment. Before enrollment on Day 1, the participants will be instructed to download the eDiary application or will be provided an eDiary device to record solicited ARs (Section 8.4.3).

Participants will be instructed on Day 1 on thermometer usage to measure body temperature, ruler usage to measure injection site erythema and swelling/induration (hardness), and

self-assessment for localized axillary swelling or tenderness on the same side as the injection arm.

Participants will record data into the eDiary starting approximately 1 hour after injection under supervision of the study site staff to ensure successful entry of assessments. The study site staff will perform any retraining as necessary. Participants will continue to record data in the eDiary after they leave the study site, preferably in the evening and at the same time each day, on the day of injection, and for 6 days following injection.

Participants will record the following data in the eDiary:

- Solicited local and systemic reactogenicity ARs, as defined in Section 8.4.3, that occur on the day of IP administration and for 6 days following IP administration.
- Daily oral body temperature measurement should be performed at approximately the same time each day using the thermometer provided by the study site. If body temperature is taken more than once in a given day, only the highest temperature reading should be recorded.
- Measurements, as applicable, for solicited local ARs (injection site erythema and swelling/induration) will be performed using the ruler provided by the study site.
- Any medications taken to treat or prevent pain or fever on the day of injection or for the next 6 days.

The eDiary will be the only source document allowed for solicited systemic or local ARs (including body temperature measurements). Participants will be instructed to complete eDiary entries daily. Quantitative temperature recordings and measurement of any injection site erythema or swelling/induration reported on the following day may be excluded from the analyses of solicited ARs.

Study site staff will review eDiary data with participants at a visit 7 days after the injection.

8.1.1.1. Ancillary Supplies for Participant Use

Study sites will distribute Sponsor-provided oral thermometers and rulers for use by participants in assessing body temperature and injection site reactions, for recording solicited ARs in eDiaries. Based on availability, smartphone devices may be provided to those participants who do not have their own device to use for eDiary activities.

8.1.2. Safety Phone Calls

A safety telephone call is a telephone call made to the participant by trained site personnel. This call will follow a script, which will facilitate the collection of relevant safety information. Safety

telephone calls follow a schedule for each participant, as shown in the SoE (Table 1). The participant will be interviewed according to the script about occurrence of MAAEs, SAEs, AESIs, or AEs leading to study withdrawal and concomitant medications associated with those events, receipt of any nonstudy vaccinations, and pregnancy (Section 8.4). All safety information collected from the telephone contact must be documented in source documents as described by the participant and not documented on the script used for the safety telephone contact. An unscheduled follow-up safety call may be triggered if an eDiary record results in identification of a relevant safety event.

8.1.3. Safety Laboratory Assessments

Planned blood sampling for safety laboratory assessments will occur as indicated in the SoE (Table 1). Tests will include WBC count, hemoglobin, platelets, alanine aminotransferase, aspartate aminotransferase, total bilirubin, alkaline phosphatase, and creatinine. Laboratory tests will be performed by the central laboratory, unless otherwise specified.

Pregnancy testing include the following:

- A point-of-care urine pregnancy test will be performed at the Screening Visit and before the study vaccination on Day 1. At any time, a pregnancy test either via blood or point-of-care urine can be performed, at the discretion of the Investigator.
- If not documented in a female participant’s medical records, an FSH test may be performed at the Screening Visit, as necessary and at the discretion of the Investigator, to confirm postmenopausal status.

8.1.4. Vital Sign Measurements

Vital sign measurements will include systolic and diastolic BP, heart rate, respiratory rate, and body temperature (preferred route is oral). The participant will be seated for at least 5 minutes before all measurements are taken. Vital signs will be measured at the time points indicated in the SoE (Table 1). On the day of IP administration, vital sign measurements will be collected once before injection and approximately 1 hour after injection (before participants are discharged from the study site). Vital signs may be collected at other study visits in conjunction with a symptom-directed physical examination.

If any of the vital sign measurements following administration of study vaccination meet the toxicity grading criteria for clinical abnormalities of Grade 3 or greater, the abnormal value and grade will be documented in the AE section of the eCRF (unless there is another known cause of the abnormality that would result in an AE classification). The Investigator will continue to monitor the participant with additional assessments until the vital sign value has reached the

reference range, returns to the vital sign value at baseline, is considered stable, or until the Investigator determines that follow-up is no longer medically necessary.

Febrile (≥ 38.0°C/100.4°F) participants on the day of IP administration may be rescheduled within the relevant window periods. Afebrile participants with minor illnesses may be injected at the discretion of the Investigator.

When procedures overlap and are scheduled to occur at the same time point, the order of procedures should be vital sign measurements and then blood collection.

8.1.5. Physical Examinations

A full physical examination, including height and weight, will be performed at scheduled time points as indicated in the SoE (Table 1). The full examination will include assessment of skin, head, ears, eyes, nose, throat, neck, thyroid, lungs, heart, cardiovascular system, abdomen, lymph nodes, and musculoskeletal system and extremities. Any clinically significant finding identified during a study visit before administration of study vaccine should be recorded in the participant's medical history eCRF. Any clinically significant finding identified during a study visit after administration of study vaccine should be reported as an MAAE (Section 8.4.4).

Symptom-directed physical examinations may be performed at other time points at the discretion of the Investigator. On the day of IP administration before injection, the arm receiving the injection and the axillary lymph nodes should be examined, and any abnormalities should be documented.

Body mass index will be calculated at the Screening Visit only.

8.1.6. Electrocardiogram

A 12-lead electrocardiogram (ECG) will be obtained after 10 minutes of supine rest at Visit 1/Day 1 prior to vaccination. Skin preparation should be thorough and electrode placement should be according to standard 12-lead ECG procedure. The purpose of the ECG is to have a baseline comparison for possible subsequent clinical evaluations of suspected myocarditis/pericarditis. Interpretation of the ECG is not required prior to study vaccine administration.

8.1.7. Assessments for Respiratory Viral Infections

Due to the ongoing COVID-19 pandemic, participants may get infected with SARS-CoV-2 or experience symptoms consistent with ILI during the study.

All participants will provide NP swab samples before the injection on Day 1 for assessment of asymptomatic infection with respiratory pathogens, including influenza virus and SARS-CoV-2, as influenza or COVID-19 symptoms may confound reactogenicity assessment (CDC 2021a).

If symptoms occur, participants will be directed to return to the study site as soon as possible, but no later than 72 hours after the onset of symptoms, for medical evaluation and an NP swab. The NP swab sample will be tested by multiplex RT-PCR for respiratory pathogens, including influenza viruses and SARS-CoV-2. Additionally, clinical information may be collected to evaluate the severity of the clinical case (CDC 2020b). All findings will be recorded in the eCRF.

If scheduled, a study site illness visit may include additional assessments such as medical history, physical examination, and blood sampling for clinical laboratory testing. Radiologic imaging studies may be conducted. Blood samples may be collected for potential future immunologic assessment of influenza and/or SARS-CoV-2 infection.

If participants are confirmed to have SARS-CoV-2 infection during the study and are asymptomatic, the Investigator will notify the participant's primary care physician and local health authority, per local regulations. If the participant had known exposure to COVID-19 (eg, exposure to someone with a confirmed case of COVID-19) prior to Day 1, it will be captured on the COVID-19 exposure form, and the IP administration for that participant will be delayed for at least 14 days.

All cases of RT-PCR-confirmed ILI and/or COVID-19 should be captured as MAAEs (Section 8.4.4; unless the definition for an SAE is met) along with relevant concomitant medications and details about severity, seriousness, and outcome.

8.2. Immunogenicity Assessments

Blood samples for immunogenicity assessments will be collected at the time points indicated in the SoE (Table 1). The following assessments are planned:

- Serum antibody level as measured by HAI assay (primary, secondary, and exploratory endpoints).
- NA-specific antibody levels as measured by NAI assay (primary, secondary, and exploratory endpoints).
- Serum neutralizing antibody level as measured by microneutralization (MN) assay or similar method (secondary or exploratory endpoint).
- Cellular immunogenicity (exploratory endpoint for subset only).

Sample aliquots will be designed to ensure that backup samples are available and that vial volumes are likely to be adequate for future testing needs. The actual time and date of each sample collected will be recorded in the eCRF, and unique sample identification will be utilized to maintain the blind at the laboratory at all times and to allow for automated sample tracking

and housing. Handling and preparation of the samples for analysis, as well as shipping and storage requirements, will be provided in a separate study manual.

Measurement of antibody levels will be performed in a laboratory designated by the Sponsor.

According to the ICF (Section 11.1.6), serum from immunogenicity testing may be used for future research, which may be performed at the discretion of the Sponsor to further characterize the immune response to influenza viruses, additional assay development, and the immune response across influenza viruses.

The maximum amount of blood collected from each participant over the duration of the study, including any extra assessments that may be required, will not exceed 450 mL for the study. Repeat or unscheduled samples may be taken for safety reasons or for technical issues with the samples. Further details are provided in both the ICF and Laboratory Reference Manual.

8.3. Efficacy Assessments

While the study will not be powered for efficacy assessments, ILI will be tracked as an exploratory objective (Section 8.1.7).

8.4. Safety Definitions and Related Procedures

8.4.1. Adverse Event

An AE is defined as any untoward medical occurrence associated with the use of a pharmaceutical product, whether or not considered related to the product.

Events Meeting the Adverse Event Definition

- Exacerbation of a chronic or intermittent pre-existing condition including either an increase in frequency and/or intensity of the condition, after the IP injection.
- New conditions detected or diagnosed after the IP injection even though they may have been present before the start of the study.

Events NOT Meeting the Adverse Event Definition

- Procedures planned before study entry (eg, hospitalization for preplanned surgical procedure).
- Medical or surgical procedure (eg, endoscopy, appendectomy): the condition that leads to the procedure should be the AE.
- Situations in which an untoward medical occurrence did not occur (social and/or convenience admission to a hospital).

An AR is any AE for which there is a reasonable possibility that the IP caused the AE (Section 8.4.3). For the purposes of investigational new drug safety reporting, "reasonable possibility" means that there is evidence to suggest a causal relationship between the IP and the AE.

An unsolicited AE is any AE reported by the participant that is not specified as a solicited AR in the protocol or is specified as a solicited AR but starts outside the protocol-defined period for reporting solicited ARs (ie, for the 7 days after the injection of IP).

8.4.2. Serious Adverse Events

An AE (including an AR) is considered an SAE if, in the view of either the Investigator or Sponsor, it results in any of the following outcomes:

- **Death**
  A death that occurs during the study or that comes to the attention of the Investigator during the protocol-defined follow-up period must be reported to the Sponsor, whether or not it is considered related to IP.
- **Is life-threatening**
  The participant was at immediate risk of death at the time of the event. It does not include an AE that, had it occurred in a more severe form, might have caused death.
- **Inpatient hospitalization or prolongation of existing hospitalization**
  In general, inpatient hospitalization indicates the participant was admitted to the hospital or emergency ward for at least 1 overnight stay for observation and/or treatment that would not have been appropriate in the physician's office or outpatient setting. The hospital or emergency ward admission should be considered an SAE regardless of whether opinions differ as to the necessity of the admission. Complications that occur during inpatient hospitalization will be recorded as AEs; however, if a complication/AE prolongs hospitalization or otherwise fulfills SAE criteria, the complication/AE will be recorded as a separate SAE.
- **Persistent or significant incapacity or substantial disruption of the ability to conduct normal life functions**
  This definition is not intended to include experiences of relatively minor medical significance such as uncomplicated headache, nausea/vomiting, diarrhea, influenza, and accidental trauma (eg, sprained ankle) which may interfere with or prevent everyday life functions but do not constitute a substantial disruption.
- **Congenital anomaly or birth defect**

- **Medically important event**
  Medical judgment should be exercised in deciding whether SAE reporting is appropriate in other situations, such as important medical events that may not be immediately life-threatening or result in death or hospitalization but may jeopardize the participant or require medical or surgical intervention to prevent one of the other outcomes listed in the above definition. These events should usually be considered serious. Examples of such medical events include allergic bronchospasm requiring intensive treatment in an emergency room or at home, blood dyscrasias, or convulsions that do not result in inpatient hospitalization, or the development of drug dependency or drug abuse.

8.4.3. Solicited Adverse Reactions

The term "reactogenicity" refers to the occurrence and intensity of selected signs and symptoms (ARs) occurring after IP injection. The eDiary will solicit daily participant reporting of ARs using a structured checklist (Section 8.1.1). Participants will record such occurrences in an eDiary on the day of IP injection and for the 6 days after the day of dosing.

Severity grading of reactogenicity will occur automatically based on participant entry into the eDiary according to the grading scales presented in Table 7 modified from the Toxicity Grading Scale for Healthy Adult and Adolescent Volunteers Enrolled in Preventive Vaccine Clinical Trials (DHHS 2007b).

If a participant reported a solicited AR during the solicited period and did not record the event in the eDiary, the event should be recorded on the Reactogenicity page of the eCRF. If the events starts during the solicited period, but continues beyond 7 days after dosing, the participant should notify the site to provide an end date to close out the event on the Reactogenicity page of the eCRF. If the participant reported an event after the solicited period (ie, after Day 7). it should be recorder as an AE on the AE page of the eCRF. All solicited ARs (local and systemic) will be considered causally related to dosing or study vaccination.

Adverse reactions beyond Day 7 should be reviewed by the study site staff either during the next scheduled phone call or at the following study visit.

Table 7: Solicited Adverse Reactions and Grades

Reaction	Grade 0 (None)	Grade 1 (Mild)	Grade 2 (Moderate)	Grade 3 (Severe)	Grade 4 (Life-Threatening)
**Local**					
**Injection site pain**	None	No interference with activity	Some interference with activity	Prevents daily activity	Requires emergency room visit or hospitalization
**Injection site erythema (redness)**	< 25 mm/ < 2.5 cm	25 - 50 mm/ 2.5 - 5 cm	51 - 100 mm/ 5.1 - 10 cm	> 100 mm/ > 10 cm	Necrosis or exfoliative dermatitis
**Injection site swelling/induration (hardness)**	< 25 mm/ < 2.5 cm	25 - 50 mm/ 2.5 - 5 cm	51 - 100 mm/ 5.1 - 10 cm	> 100 mm/ > 10 cm	Necrosis
**Axillary (underarm) swelling or tenderness ipsilateral to the side of injection**	None	No interference with activity	Some interference with activity	Prevents daily activity	Requires emergency room visit or hospitalization
**Systemic**					
**Headache**	None	No interference with activity	Some interference with activity	Prevents daily activity	Requires emergency room visit or hospitalization
**Fatigue**	None	No interference with activity	Some interference with activity	Significant; prevents daily activity	Requires emergency room visit or hospitalization
**Myalgia (muscle aches all over body)**	None	No interference with activity	Some interference with activity	Significant; prevents daily activity	Requires emergency room visit or hospitalization
**Arthralgia (joint aches in several joints)**	None	No interference with activity	Some interference with activity	Significant; prevents daily activity	Requires emergency room visit or hospitalization

Reaction	Grade 0 (None)	Grade 1 (Mild)	Grade 2 (Moderate)	Grade 3 (Severe)	Grade 4 (Life-Threatening)
**Nausea/vomiting**	None	No interference with activity or 1-2 episodes/ 24 hours	Some interference with activity or > 2 episodes/24 hours	Prevents daily activity, requires outpatient intravenous hydration	Requires emergency room visit or hospitalization for hypotensive shock
**Chills**	None	No interference with activity	Some interference with activity not requiring medical intervention	Prevents daily activity and requires medical intervention	Requires emergency room visit or hospitalization
**Fever (oral)**	< 38.0°C<br>< 100.4°F	38.0 – 38.4°C<br>100.4 – 101.1°F	38.5 – 38.9°C<br>101.2 – 102.0°F	39.0 – 40.0°C<br>102.1 – 104.0°F	> 40.0°C<br>> 104.0°F

Note: Events listed above but starting > 7 days post study injection will be recorded on the AE page of the eCRF. Causality for each event will be determined per assessment by the Investigator.
Source: DHHS 2007

Any solicited AR that meets any of the following criteria must be entered into the participant's source document and must also be recorded by the study site staff in the solicited AR section of the participant's eCRF:

- Solicited local or systemic AR that results in a visit to a healthcare practitioner (HCP), to be recorded as an MAAE (Section 8.4.4).
- Solicited local or systemic AR leading to the participant withdrawing from the study or the participant being withdrawn from the study by the Investigator (AE leading to withdrawal).
- Solicited local or systemic AR continuing beyond 7 days post injection.
- Solicited local or systemic AR that otherwise meets the definition of an SAE.

8.4.4. Medically Attended Adverse Events

An MAAE is an AE that leads to an unscheduled visit to an HCP. This would include visits to a study site for unscheduled assessments (eg, abnormal laboratory follow-up and/or COVID-19 [Section 8.1.7]) and visits to HCPs external to the study site (eg, urgent care, primary care physician). Investigators will review unsolicited AEs for the occurrence of any MAAEs. Unsolicited AEs will be captured on the AE page of the eCRF.

All cases of RT-PCR-confirmed ILI and/or confirmed COVID-19 will be recorded as MAAEs.

8.4.5. Influenza-Like Illness

A CDC-defined ILI is defined as body temperature ≥ 37.8°C (100°F) accompanied by cough and/or sore throat.

An RT-PCR confirmed ILI is defined as a positive influenza result by RT-PCR done at any setting during the study period.

8.4.6. Adverse Events of Special Interest

An AESI is an AE (serious or nonserious) of scientific and medical concern specific to the Sponsor's product or program, for which ongoing monitoring and immediate notification by the Investigator to the Sponsor are required. Such events may require further investigation to characterize and understand them.

The AESIs for this protocol are listed in Appendix 3 (Section 11.3).

Protocol-specific AESIs will be collected through the entire study period and must be reported to the Sponsor or designee immediately and in all circumstances within 24 hours of becoming aware of the event via the electronic data capture (EDC) system. If a site receives a report of a new AESI from a participant or receives updated information on a previously reported AESI at a time after the eCRF has been taken offline, then the site can report this information on a paper AESI form using the SAE Mailbox or the SAE Fax line (Section 8.4.12).

Myocarditis/Pericarditis

A case of suspected, probable, or confirmed myocarditis, pericarditis, or myopericarditis should be reported as an AESI, even if it does not meet criteria per the CDC case definition. The event should also be reported as an SAE if it meets seriousness criteria (Section 8.4.2). The CDC case definition is provided in Appendix 4 (Section 11.4) as guidance.

Anaphylaxis

All suspected cases of anaphylaxis should be recorded as MAAEs and reported as SAEs, based on the criteria for a medically important event, unless the event meets other serious criteria. As an SAE, the event should be reported to the Sponsor or designee immediately and in all circumstances within 24 hours, per Section 8.4.12. The Investigator will submit any updated anaphylaxis case data to the Sponsor within 24 hours of it being available. For reporting purposes, a participant who displays signs or symptoms consistent with anaphylaxis (as described below) should be reported as a potential case of anaphylaxis. This is provided as general guidance for Investigators and is based on the Brighton Collaboration case definition (Rüggeberg et al 2007).

Anaphylaxis is an acute hypersensitivity reaction with multi-organ system involvement that can present as, or rapidly progress to, a severe life-threatening reaction. It may occur following exposure to allergens from a variety of sources.

Anaphylaxis is a clinical syndrome characterized by the following:

- Sudden onset AND
- Rapid progression of signs and symptoms AND
- Involves 2 or more organ systems, as follows:
  - **Skin/mucosal:** urticaria (hives), generalized erythema, angioedema, generalized pruritus with skin rash, generalized prickle sensation, red and itchy eyes.
  - **Cardiovascular:** measured hypotension, clinical diagnosis of uncompensated shock, loss of consciousness or decreased level of consciousness, evidence of reduced peripheral circulation.
  - **Respiratory:** bilateral wheeze (bronchospasm), difficulty breathing, stridor, upper airway swelling (lip, tongue, throat, uvula, or larynx), respiratory distress, persistent dry cough, hoarse voice, sensation of throat closure, sneezing, rhinorrhea.
  - **Gastrointestinal:** diarrhea, abdominal pain, nausea, vomiting.

8.4.7. Recording and Follow-up of Pregnancy

Female participants who have a positive pregnancy test at screening should not be enrolled; participants who have a positive pregnancy test at Day 1 should not receive the IP.

Details of all pregnancies in female participants will be collected after the study vaccination on Day 1 and until the end of their participation in the study. If a pregnancy is reported, the Investigator should inform the Sponsor within 24 hours of learning of the pregnancy and should follow the procedures outlined in this section. Abnormal pregnancy outcomes (eg, spontaneous abortion, fetal death, stillbirth, congenital anomalies, ectopic pregnancy) are considered SAEs.

Pregnancies occurring in participants after enrollment must be reported to Sponsor or designee within 24 hours of the site learning of its occurrence using the SAE Mailbox, the SAE Hotline, or the SAE Fax line (Section 8.4.12). If the participant agrees to submit this information, the pregnancy must be followed to determine the outcome, including spontaneous or voluntary termination, details of the birth, and the presence or absence of any birth defects, congenital abnormalities, or maternal and/or newborn complications. This follow-up should occur even if intended duration of the safety follow-up for the study has ended. Pregnancy report forms will be distributed to the study site to be used for this purpose. The Investigator must immediately

(within 24 hours of awareness) report to the Sponsor any pregnancy resulting in an abnormal outcome according to the procedures described for SAEs.

8.4.8. Eliciting and Documenting Adverse Events

The Investigator is responsible for ensuring that all AEs and SAEs are recorded in the eCRF and reported to the Sponsor.

Solicited ARs will be collected from Day 1 through 7 days after the vaccine dose. Other (unsolicited) AEs will be collected from Day 1 through 28 days after the vaccine dose.

AESIs, MAAEs, SAEs, and AEs leading to withdrawal will be collected from Day 1 throughout entire study duration (Day 181/EoS for all participants) as specified in the SoE (Table 1). Any AEs occurring before receipt of IP will be analyzed separately from AEs.

At every study site visit or telephone contact, participants will be asked a standard question to elicit any medically related changes in their well-being (including respiratory viral infection symptoms) according to the scripts provided. Participants will also be asked if they have been hospitalized, had any accidents, used any new medications, changed concomitant medication regimens (both prescription and over-the-counter medications), or had any nonstudy vaccinations.

In addition to participant observations, physical examination findings, and other documents relevant to participant safety classified as an AE will be documented in the AE section of the eCRF.

After the initial AE/SAE report, the Investigator is required to proactively follow each participant at subsequent visits/contacts. All AEs and SAEs will be treated as medically appropriate and followed until resolution, stabilization, the event is otherwise explained, or the participant is LTFU (as defined in Section 7.4).

8.4.9. Assessment of Intensity

An event is defined as “serious” when it meets at least one of the predefined outcomes as described in the definition of an SAE (Section 8.4.2), NOT when it is rated as severe.

The severity (or intensity) of an AR or AE refers to the extent to which it affects the participant’s daily activities. The Toxicity Grading Scale for Healthy Adult and Adolescent Volunteers Enrolled in Preventive Vaccine Clinical Trials (DHHS 2007b) will be used to categorize local and systemic reactogenicity events (solicited ARs), clinical laboratory test results, and vital sign measurements observed during this study. Specific criteria for local and systemic reactogenicity events are presented in Section 8.4.3.

The determination of severity for all unsolicited AEs should be made by the Investigator based upon medical judgment and the definitions of severity as follows:

- Mild: These events do not interfere with the participant's daily activities.
- Moderate: These events cause some interference with the participant's daily activities and require limited or no medical intervention.
- Severe: These events prevent the participant's daily activity and require intensive therapeutic intervention.

Study staff should elicit from the participant the impact of AEs on the participant's activities of daily living to assess severity and document appropriately in the participant's source documentation. Changes in the severity of an AE should be documented in the participant's source documentation to allow an assessment of the duration of the event at each level of intensity to be performed. An AE characterized as intermittent requires documentation of onset and duration of each episode. An AE that fluctuates in severity during the course of the event is reported once in the eCRF at the highest severity observed.

8.4.10. Assessment of Causality

The Investigator's assessment of an AE's relationship to IP is part of the documentation process but is not a factor in determining what is or is not reported in the study.

The Investigator will assess causality (ie, whether there is a reasonable possibility that the IP caused the event) for all AEs and SAEs. The relationship will be characterized using the following classification:

Not related: There is not a reasonable possibility of a relationship to the IP. Participant did not receive the IP OR temporal sequence of the AE onset relative to administration of the IP is not reasonable OR the AE is more likely explained by another cause than the IP.

Related: There is a reasonable possibility of a relationship to the IP. There is evidence of exposure to the IP. The temporal sequence of the AE onset relative to the administration of the IP is reasonable. The AE is more likely explained by the IP than by another cause.

8.4.11. Reporting Adverse Events

The Investigator is responsible for reporting all AEs that are observed or reported during the study regardless of their relationship to the IP or their clinical significance. If there is any doubt as to whether a clinical observation is an AE, the event should be reported.

All unsolicited AEs reported or observed during the study will be recorded in the AE section of the eCRF. Information to be collected includes type of event, time of onset,

Investigator-specified assessment of severity (impact on activities of daily living) and relationship to the IP, time of resolution of the event, seriousness, any required treatment or evaluations, and outcome. The unsolicited AEs resulting from concurrent illnesses, reactions to concurrent illnesses, reactions to concurrent medications, or progression of disease states must also be reported. All AEs will be followed until they are resolved or stable or judged by the Investigator to be not clinically significant. The Medical Dictionary for Regulatory Activities (MedDRA) will be used to code all unsolicited AEs.

Any medical condition that is present at the time that the participant is screened but does not deteriorate should not be reported as an unsolicited AE. However, if it deteriorates at any time during the study, it should be recorded as an unsolicited AE.

8.4.12. Reporting Serious Adverse Events

Prompt notification by the Investigator to the Sponsor of an SAE is essential so that legal obligations and ethical responsibilities toward the safety of participants and the safety of a study intervention under clinical investigation are met.

Any AE considered serious by the Investigator or that meets SAE criteria (Section 8.4.2) must be reported to the Sponsor immediately (within 24 hours of becoming aware of the SAE) via the EDC system. The Investigator will assess whether there is a reasonable possibility that the IP caused the SAE. The Sponsor will be responsible for notifying the relevant regulatory authorities of any SAE as outlined in 21 US CFR Parts 312 and 320. The Investigator is responsible for notifying the institutional review board (IRB) directly.

If the eCRF is unavailable at the time of the SAE, the following contact information is to be used for SAE reporting:

- SAE Mailbox: Safety_Moderna@iqvia.com
- SAE Hotline (USA): +1-866-599-1341
- SAE Fax Line (USA): +1-866-599-1342

Regulatory reporting requirements for SAEs are described in Section 8.4.17.

The Investigator and any qualified designees are responsible for detecting, documenting, and recording events that meet the definition of an AE, including SAEs, and remain responsible for following up AEs that are serious, considered related to the IP or study procedures, or that caused the participant to discontinue the study.

8.4.13. Reporting of Adverse Events of Special Interest

The following process for reporting an AESI ensures compliance with 21 CFR 312 and ICH GCP guidelines. After learning that a participant has experienced an AESI, the Investigator or designee is responsible for reporting the AESI to the Sponsor, regardless of relationship or expectedness, within 24 hours of becoming aware of the event. If the AESI meets the criteria for an SAE, the SAE reporting procedure should be followed.

8.4.14. Time Period and Frequency for Collecting Adverse Event and Serious Adverse Event Information

Medical occurrences that begin before the start of IP administration but after obtaining informed consent will be recorded on the Medical History/Current Medical Conditions section of the eCRF and not in the AE section; however, if the condition worsens at any time after IP administration during the study, it will be recorded and reported as an AE.

Adverse events may be collected as follows:

- Observing the participant.
- Receiving an unsolicited complaint from the participant.
- Questioning the participant in an unbiased and nonleading manner.

Solicited ARs will be collected from the day of vaccination through 6 days after injection. Other (unsolicited) AEs will be collected from the day of vaccination through 28 days after the injection.

Serious AEs will be collected from the start of IP administration until the last day of study participation.

All SAEs will be recorded and reported to the Sponsor or designee immediately and under no circumstance should this exceed 24 hours of becoming aware of the event via the EDC system. If a site receives a report of a new SAE from a study participant or receives updated data on a previously reported SAE and the eCRF has been taken offline, then the site can report this information on a paper SAE form using the SAE Mailbox or the SAE Fax line (Section 8.4.12).

An abnormal value or result from a clinical or laboratory evaluation can also indicate an AE if it is determined by the Investigator to be clinically significant (eg, meets any serious criteria). If this is the case, it must be recorded in the source document and as an AE on the appropriate AE form(s). The evaluation that produced the value or result should be repeated until that value or result returns to normal or is stabilized and the participant’s safety is not at risk.

Investigators are not obligated to actively seek AEs or SAEs after EoS. However, if the Investigator learns of any SAE (including a death) at any time after a participant has withdrawn

from or completed the study and the Investigator considers the event to be reasonably related to the IP or study participation, the Investigator must promptly notify the Sponsor.

Participants who develop ILI will be followed through 30 days from the onset of ILI even if Day 30 is beyond Day 181/EoS.

8.4.15. Method of Detecting Adverse Events and Serious Adverse Events

The method of recording, evaluating, and assessing causality of AE and SAE and the procedures for completing and transmitting SAE reports are provided in Section 8.4.12.

Electronic diaries have specifically been designed for this study by the Sponsor. The diaries will include prelisted AEs (solicited ARs) and intensity scales; they will also include blank space for the recording of information on other AEs (unsolicited AEs) and concomitant medications/vaccinations.

The Investigator is responsible for the documentation of AEs regardless of the vaccination group or suspected causal relationship to the IP. For all AEs, the Investigator must pursue and obtain information adequate to determine the outcome of the AE and to assess whether the AE meets the criteria for classification as an SAE, which requires immediate notification to the Sponsor or its designated representative.

Care will be taken not to introduce bias when detecting AEs and/or SAEs. Open-ended and nonleading verbal questioning of the participant is the preferred method to inquire about AE occurrences.

8.4.16. Follow-up of Adverse Events, Serious Adverse Events, and Adverse Events of Special Interest

After the initial AE/SAE report, the Investigator is required to proactively follow each participant at subsequent visits and contacts.

All AEs, SAEs, and nonserious AESIs (as defined in Section 8.4.6), will be treated as medically appropriate and followed until resolution, stabilization, the event is otherwise explained, or the participant is LTFU, as defined in Section 7.4.

8.4.17. Regulatory Reporting Requirements for Serious Adverse Events

Prompt notification by the Investigator to the Sponsor of a SAE is essential so that legal obligations and ethical responsibilities towards the safety of participants and the safety of a study intervention under clinical investigation are met.

The Sponsor has a legal responsibility to notify both the local regulatory authority and other regulatory agencies about the safety of a study intervention under clinical investigation. The

Sponsor will comply with country-specific regulatory requirements relating to safety reporting to the regulatory authority, IRBs, and Investigators.

Investigator safety reports must be prepared for suspected unexpected serious ARs according to local regulatory requirements and Sponsor policy and forwarded to Investigators as necessary.

An Investigator who receives an Investigator safety report describing an SAE or other specific safety information (eg, summary or listing of SAEs) from the Sponsor will review and then file it along with the IB and will notify the IRB, if appropriate according to local requirements.

8.5. Safety Monitoring

The CRO's medical monitor, the Sponsor's medical monitor, and the individual Site Investigators will monitor safety throughout the study. The roles of the IST and DSMB are outlined in Section 11.1.11.

8.6. Treatment of Overdose

Because the IP is to be administered by an HCP, it is unlikely that an overdose will occur. Dose deviations will be tracked as protocol deviations (Section 11.1.8).

8.7. Pharmacokinetics

Pharmacokinetic parameters are not evaluated in this study.

8.8. Pharmacodynamics

Pharmacodynamic parameters are not evaluated in this study.

8.9. Biomarkers

Immunogenicity assessments are described in Section 8.2. Biomarker assessments (to be determined) will be evaluated in this study, which may include genomic and transcriptomic studies.

8.10. Health Economics

Health economics are not evaluated in this study.

9. STATISTICAL ANALYSIS PLAN

This section summarizes the planned statistical analysis strategy and procedures for the study. The details of statistical analysis will be provided in the statistical analysis plan (SAP), which will be finalized before the clinical database lock for the study. If changes are made to primary and/or key secondary objectives or the related statistical methods after the study has begun but prior to any data unblinding, then the protocol will be amended (consistent with ICH Guideline E9). Changes to other secondary or exploratory analyses made after the protocol has been finalized, along with an explanation as to when and why they occurred, will be listed in the SAP or clinical study report (CSR) for the study. Ad hoc exploratory analyses, if any, will be clearly identified in the CSR.

9.1. Blinding and Responsibility for Analyses

This is an observer-blind study. The Investigator, study staff, study participants, site monitors, and Sponsor personnel (or its designees) will be blinded to the IP administered until the study database is locked and unblinded, with the following exceptions:

- Unblinded personnel (of limited number) will be assigned to vaccine accountability procedures and will prepare the IP for all participants. These personnel will have no study functions other than IP management, documentation, accountability, preparation, and administration. They will not be involved in participant evaluations and will not reveal the identity of the IP to either the participant or the blinded study site personnel involved in the conduct of the study unless this information is necessary in the case of an emergency.
- Unblinded medically qualified study site personnel will administer the IP. They will not be involved in assessments of any study endpoints.
- Unblinded site monitors, not involved in other aspects of monitoring, will be assigned as the IP accountability monitors. They will have responsibilities to ensure that sites are following all proper IP accountability, preparation, and administration procedures.
- An independent unblinded statistical and programming team will perform the preplanned IA (Section 9.6). Sponsor team members will be prespecified to be unblinded to the IA results and will not communicate the results to the blinded Investigators, study site staff, clinical monitors, or participants.
- The DSMB will review the IA data to safeguard the interests of clinical study participants and to help ensure the integrity of the study. The DSMB will review unblinded IA results provided by the independent unblinded statistician. The DSMB will also review

unblinded statistical outputs for ad hoc safety reviews triggered by pause rules, should this occur. Section 11.1.11 provides additional information on DSMB and safety review.

The dosing assignment will be concealed by having the unblinded pharmacy personnel prepare the IP in a secure location that is not accessible or visible to other study staff. An opaque sleeve over the syringe used for injection will maintain the blind at the time of injection, as the doses containing mRNA IP will look different from that of the active comparator Flublok. Only delegated unblinded study site staff will conduct the injection procedure. Once the injection is completed, only the blinded study staff will perform further assessments and interact with the participants. Access to the randomization code will be strictly controlled at the pharmacy.

9.1.1. Breaking the Blind

Except in the case of medical necessity, a participant's vaccine assignment should not be unblinded without the approval of the Sponsor. If a participant becomes seriously ill or pregnant during the study, the blind will be broken only if knowledge of the vaccine assignment will affect that participant's clinical management. In the event of a medical emergency requiring identification of individual vaccine assignment, the Investigator will make every attempt to contact the CRO's medical monitor, preferably via electronic protocol inquiry platform, to explain the need for unblinding within 24 hours of opening the code. The Investigator will be responsible for documenting the time, date, reason for unblinding, and the names of the personnel involved. The Investigator (or designee) will have access to unblind participants within interactive response technology (IRT). All unblinding instances will be tracked via an audit trail in IRT and documented in the final study report.

In addition to the aforementioned situations where the blind may be broken, the data will also be unblinded to a statistical team at specified time point(s) for analysis as outlined in Section 9.6.

9.2. Statistical Hypotheses

No formal hypotheses will be tested.

9.3. Sample Size Determination

The sample size for this study is not driven by statistical assumptions for formal hypothesis testing. The number of proposed participants is considered sufficient to provide a descriptive summary of the safety and immunogenicity of different dose levels of mRNA-1020 or mRNA-1030.

A total of approximately 560 participants will be randomly assigned to receive mRNA-1020, mRNA-1030, mRNA-1010, or Flublok. Among those ~560 participants, 70 participants will be

randomized into each vaccination group. Details regarding the number of participants in each vaccination group and the randomization ratio are presented in Section 6.2.

With 70 participants in each group receiving the IP, there is an approximately 76% probability to observe at least 1 participant with an AE if the true incidence of the AE is 2%; if the true incidence rate is 4%, then the probability to observe an AE is approximately 94%.

9.4. Analysis Sets

Table 8 describes the analysis datasets.

Table 8: Analysis Sets

Set	Description
Randomization Set	The Randomization Set consists of all participants who are randomly assigned.
Full Analysis Set (FAS)[1]	The FAS consists of all randomly assigned participants who receive the IP.
Per-Protocol (PP) Set[2]	The PP Set consists of all participants in the FAS who comply with the injection schedule, comply with the timings of immunogenicity blood sampling to have a baseline and at least 1 postinjection assessment, do not have influenza infection at baseline through Day 29 (as documented by PCR testing), and have no major protocol deviations that impact the immune response.
Safety Set[3]	The Safety Set consists of all randomly assigned participants who receive the IP.
Solicited Safety Set[4]	The Solicited Safety Set consists of all participants in the Safety Set who contribute any solicited AR data.

Abbreviations: AR = adverse reaction; IP = investigational product; PCR = polymerase chain reaction.
1. For the FAS, participants will be analyzed according to the group to which they were randomized.
2. The PP Set will be used as the primary analysis set for analyses of immunogenicity unless otherwise specified. Participants will be analyzed according to the group to which they were randomized.
3. The Safety Set will be used for all analyses of safety, except for the solicited ARs. Participants will be included in the vaccination group corresponding to what they actually received.
4. The Solicited Safety Set will be used for the analyses of solicited ARs and participants will be included in the vaccination group corresponding to what they actually received.

9.5. Statistical Methods

General Considerations: All analyses will be performed by treatment arm, unless otherwise specified. Categorical variables will be presented by frequencies and percentages. Continuous variables will be summarized using descriptive statistics (number of participants, mean, median, standard deviation, minimum, and maximum).

9.5.1. Baseline Characteristics and Demographics

Demographic variables (eg, age, gender, race, ethnicity, height, weight, and body mass index) and baseline characteristics will be summarized by vaccination group and overall. Summary

statistics (mean and standard deviation for continuous variables, and number and percentage for categorical variables) will be provided.

9.5.2. Safety Analyses

All safety analyses will be based on the Safety Set, except summaries of solicited ARs, which will be based on the Solicited Safety Set. All safety analyses will be provided by vaccination group. Participants will be included in the vaccination group corresponding to what they actually received.

Safety and reactogenicity will be assessed by clinical review of all relevant parameters, including solicited ARs (local and systemic ARs), unsolicited AEs (including any clinical safety laboratory abnormalities), treatment-related AEs, severe AEs, SAEs, MAAEs, AEs leading to withdrawal from study participation, AESIs, vital sign measurements, and physical examination findings.

The number and percentage of participants with any solicited local AR or solicited systemic AR during the 7-day follow-up period after the study injection will be summarized. A 2-sided 95% confidence interval (CI) using the Clopper-Pearson method will also be provided for the percentage of participants with any solicited AR.

The number and percentage of participants with unsolicited AEs, treatment-related AEs, severe AEs, SAEs, AESIs, MAAEs, and AEs leading to withdrawal from study participation will be summarized. Unsolicited AEs will be coded according to the MedDRA for AR terminology and presented by MedDRA system organ class and preferred term.

Solicited ARs will be coded according to the MedDRA for AR terminology. The toxicity grading scale for healthy adult and adolescent volunteers enrolled in preventive vaccine clinical trials will be used in this study with modification for rash, solicited ARs, and vital signs (DHHS 2007b). Unsolicited AEs will be presented by MedDRA system organ class and preferred term.

The number of events of unsolicited AEs/SAEs, AESIs, MAAEs, and AEs leading to withdrawal will be reported in summary tables accordingly. For all other safety parameters, descriptive summary statistics will be provided.

Assessment of safety laboratory tests will be done for this study. The number and percentage of participants who have chemistry and hematology results below or above the normal laboratory ranges will be tabulated by time point. For treatment-emergent safety laboratory test results, the raw values and change from baseline values will be summarized by vaccination group and visit at each time point.

Vital sign results will be presented.

Table 9 summarizes the analysis strategy for safety parameters. For all other safety parameters, descriptive summary statistics will be provided. Further details will be described in the SAP.

Table 9: Analysis Strategy for Safety Parameters

Safety Endpoint	Number and Percentage of Participants, Number of Events	95% CI
Any solicited AR (overall and by local, systemic)	X	X
Any unsolicited AE	X	–
Any SAE	X	–
Any unsolicited MAAE	X	–
Any unsolicited AESI	X	–
Any unsolicited treatment-related AE	X	–
Any treatment-related SAE	X	–
Any unsolicited AE leading to withdrawal from study participation	X	–
Any severe unsolicited AE	X	–
Any treatment-related severe unsolicited AE	X	–

Abbreviations: AE = adverse event; AESI = adverse event of special interest; AR = adverse reaction; CI = confidence interval; MAAE = medically attended adverse event; SAE = serious adverse event.
Note: 95% CI using the Clopper-Pearson method. X = results will be provided.

9.5.3. Immunogenicity Analyses

Immunogenicity analyses will be reported based on the PP Set and provided by vaccination group, unless otherwise specified.

For the immunogenicity endpoints, geometric mean of specific antibody titers with corresponding 95% CI at each time point and geometric mean fold rise (GMFR) of specific antibody titers with corresponding 95% CI at each postbaseline time point over preinjection baseline at Day 1 will be provided by vaccination group. Descriptive summary statistics including median, minimum, and maximum will also be provided. Geometric mean titer (GMT) and GMFR might be adjusted for baseline titer and/or age group.

For summarizations of geometric mean titers, antibody titers reported as below lower limit of quantification (LLOQ) will be replaced by 0.5 × LLOQ. Values that are greater than the upper limit of quantification (ULOQ) will be converted to the ULOQ.

For HA, rate of seroconversion is defined as the proportion of participants with either a pre-vaccination HI titer < 1:10 and a postvaccination HI titer ≥ 1:40 or a prevaccination HI titer ≥ 1:10 and a minimum 4-fold rise in postvaccination HI antibody titer. For NA, an endpoint of interest is the percentage of participants with a change in the Day 29 titer of at least 2-/3-/4-

fold rise, defined as ≥ 2-/3-/4-fold of the LLOQ if the Day 1 titer is < LLOQ; or ≥ 2-/3-/4-fold of the Day 1 titer if the Day 1 titer is ≥ LLOQ.

Seroconversion rate from baseline will be provided with a 2-sided 95% CI using the Clopper-Pearson method at each postbaseline time point. For NA, the number and percentage of participants with a ≥ 2-, ≥ 3-, and ≥ 4-fold rise of serum titers from baseline will be provided with 2-sided 95% CI using the Clopper-Pearson method at each postbaseline time point.

Further details will be described in the SAP.

9.5.4. Exploratory Analyses

Exploratory analyses not addressed in Section 9.5.1 and Section 9.5.3 will be described in the SAP before database lock.

9.5.5. Subgroup Analyses

The protocol does not define any formal subgroup analyses. However, age-specific subgroup analyses may be performed to assess for potential differences in safety/reactogenicity or immune responses across age groups.

9.6. Study Analyses

One unblinded IA is planned in this study. It will be performed after participants have completed the Day 29 Visit and will include, at a minimum, cleaned data (ie, data that are as clean as possible) from available safety and immunogenicity datasets.

The IA will be performed by a separate team of unblinded programmers and statisticians. Except for a limited number of Sponsor and CRO personnel who will be unblinded to perform the IA, the study site staff, Investigators, study monitors, and participants will remain blinded until after the final database lock for final analysis. The final CSR will include full unblinded analyses of safety and immunogenicity data available through Day 181/EoS.

The final analysis of all endpoints will be performed after all participants have completed all planned study procedures. Results of this analysis will be presented in a final CSR, including individual listings. The final CSR will include full analyses of all safety and immunogenicity data through Day 181/EoS.

The SAP will describe the planned interim and final analyses in greater detail.

10. REFERENCES

Aretz HT, Billingham ME, Edwards WD, Factor SM, Fallon JT, Fenoglio JJ Jr, et al. Myocarditis. A histopathologic definition and classification. Am J Cardiovasc Pathol. 1987;1:3–14.

Baden LR, El Sahly HM, Essink B, Kotloff K, Frey S, Novak R, et al; COVE Study Group. Efficacy and safety of the mRNA-1273 SARS-CoV-2 vaccine. N Engl J Med. 2021;384(5):403-16.

Centers for Disease Control and Prevention (CDC). CDC seasonal flu vaccine effectiveness studies [Internet]. Atlanta (GA). 2020a Dec 11 (cited 2021 Mar 17). Available from: https://www.cdc.gov/flu/vaccines-work/effectiveness-studies.htm.

Centers for Disease Control and Prevention (CDC). U.S. influenza surveillance system: Purpose and methods. [Internet]. Atlanta (GA). 2020b (cited 2021 Sep 01). Available from: https://www.cdc.gov/flu/weekly/overview.htm.

Centers for Disease Control and Prevention (CDC). Outpatient Illness Surveillance. [Internet]. Atlanta (GA). 2020c (cited 2021 Sep 08). Available from: https://www.cdc.gov/flu/weekly/overview.htm#:~:text=Outpatient%20Illness%20Surveillance.

Centers for Disease Control and Prevention (CDC). Influenza virus testing methods [Internet]. 2020d Aug 10 (cited 2021 June 22). Available from: https://www.cdc.gov/flu/professionals/diagnosis/table-testing-methods.htm

Centers for Disease Control and Prevention (CDC). Coronavirus disease 2019 (COVID-19) 2021 case definition. [Internet]. Atlanta (GA). 2021a (cited 2021 Sep 01). Available from: https://ndc.services.cdc.gov/case-definitions/coronavirus-disease-2019-2021/.

Centers for Disease Control and Prevention (CDC). COVID-19: Appendix A – glossary of key terms: close contact [Internet]. Atlanta (GA). 2021b Aug 05 (cited 2021 Sep 01). Available from: https://www.cdc.gov/coronavirus/2019-ncov/php/contact-tracing/contact-tracing-plan/appendix.html#:~:text=who%20do%20not.-,Close%20Contact,-Close%20Contact%20through.

ClinicalTrials.gov [Internet]. Bethesda (MD): National Library of Medicine (US). Identifier NCT03382405: Safety, reactogenicity, and immunogenicity of cytomegalovirus vaccines mRNA-1647 and mRNA-1443 in healthy adults. 2017 Dec 22 (updated 2021 Jan 15; cited 2021 Mar 17). Retrieved from: https://clinicaltrials.gov/ct2/show/NCT03382405.

ClinicalTrials.gov [Internet]. Bethesda (MD): National Library of Medicine (US). Identifier NCT03392389: Safety, reactogenicity, and immunogenicity of mRNA-1653 in healthy adults.

2018 Jan 8 (updated 2020 Feb 6; cited 2021 Mar 26). Retrieved from: https://clinicaltrials.gov/ct2/show/study/NCT03392389.

ClinicalTrials.gov [Internet]. Bethesda (MD): National Library of Medicine (US). Identifier NCT04283461: Safety and immunogenicity study of 2019-nCov vaccine (mRNA-1273) for prophylaxis of SARS-CoV-2 infection (COVID-19). 2020 Feb 21 (updated 2021 Apr 9; cited 2021 Mar 17). Retrieved from: https://clinicaltrials.gov/ct2/keydates/NCT04283461.

ClinicalTrials.gov [Internet]. Bethesda (MD): National Library of Medicine (US). Identifier NCT04405076: Dose-confirmation study to evaluate the safety, reactogenicity, and immunogenicity of mRNA-1273 COVID-19 vaccine in adults aged 18 years and older. 2020 May 28 (updated 2021 Mar 21; cited 2021 Apr 10). Retrieved from: https://clinicaltrials.gov/ct2/show/NCT04405076.

ClinicalTrials.gov [Internet]. Bethesda (MD): National Library of Medicine (US). Identifier NCT04470427: A study to evaluate efficacy, safety, and immunogenicity of mRNA-1273 vaccine in adults aged 18 years and older to prevent COVID-19. 2020 Jul 14 (updated 2021 Feb 25; cited 2021 Apr 10). Retrieved from: https://clinicaltrials.gov/ct2/show/NCT04470427.

ClinicalTrials.gov [Internet]. Bethesda (MD): National Library of Medicine (US). Identifier NCT04917861: Safety, tolerability, and immunogenicity of Zika vaccine mRNA-1893 in healthy flavivirus seropositive and seronegative adults. 2019 Aug 22 (updated 2021 Mar 5; cited 2021 Mar 17). Retrieved from: https://clinicaltrials.gov/ct2/show/NCT04917861.

ClinicalTrials.gov [Internet]. Bethesda (MD): National Library of Medicine (US). Identifier NCT04956575: A Study of mRNA-1010 Seasonal Influenza Vaccine in Healthy Adults. 2021 Jul 09 (updated 2021 Jul 15; cited 2021 Oct 29). Retrieved from: https://clinicaltrials.gov/ct2/show/NCT04956575.

Department of Health and Human Services (DHHS), Food and Drug Administration, Center for Biologics Evaluation and Research (US). Guidance for industry: Clinical data needed to support the licensure of seasonal inactivated influenza vaccines. May 2007a [cited 2021 Mar 18] [17 screens]. Available from: https://www.fda.gov/files/vaccines%2C%20blood%20%26%20biologics/published/Guidance-for-Industry--Clinical-Data-Needed-to-Support-the-Licensure-of-Seasonal-Inactivated-Influenza-Vaccines.pdf.

Department of Health and Human Services (DHHS), Food and Drug Administration, Center for Biologics Evaluation and Research (US). Guidance for industry: Toxicity grading scale for healthy adult and adolescent volunteers enrolled in preventive vaccine clinical trials. September 2007b [cited 2021 Mar 18] [10 screens]. Available from:

https://www.fda.gov/downloads/BiologicsBloodVaccines/GuidanceComplianceRegulatoryInformation/Guidances/Vaccines/ucm091977.pdf.

Department of Health and Human Services (DHHS), Food and Drug Administration (US). Guidance for industry, investigators, and institutional review boards: Conduct of clinical trials of medical products during the COVID-19 public health emergency. March 2020 [updated 2021 Jan 27; cited 2021 Mar 18] [38 screens]. Available from: https://www.fda.gov/media/136238/download.

Dunning AJ, DiazGranados CA, Voloshen T, Hu B, Landolfi VA, Talbot HK. Correlates of protection against influenza in the elderly: results from an influenza vaccine efficacy trial. Clin Vaccine Immunol. 2016;23(3):228-35.

European Medicines Agency (EMA). Committee for Medicinal Products for Human Use. Guideline on influenza vaccines: non-clinical-clinical and clinical module. London, 21 July 2016. EMA/CHMP/VWP/457259/2014 [cited 2021 Mar 18] [31 screens]. Available from: https://www.ema.europa.eu/en/documents/scientific-guideline/influenza-vaccines-non-clinical-clinical-clinical-module_en.pdf.

Feldman RA, Fuhr R, Smolenov I, Ribeiro AM, Panther L, Watson M, et al. mRNA vaccines against H10N8 and H7N9 influenza viruses of pandemic potential are immunogenic and well tolerated in healthy adults in Phase 1 randomized clinical trials. Vaccine. 2019;37(25):3326-34.

Gargano JW, Wallace M, Hadler SC, Langley G, Su JR, Oster ME, et al. Use of mRNA COVID-19 vaccine after reports of myocarditis among vaccine recipients: update from the Advisory Committee on Immunization Practices – United States, June 2021. MMWR Morb Mortal Wkly Rep. 2021;70(27):977-82.

Jackson LA, Anderson EJ, Rouphael NG, Roberts PC, Makhene M, Coler RN, et al. mRNA-1273 Study Group. An mRNA vaccine against SARS-CoV-2 – preliminary report. N Engl J Med. 2020;383(20):1920-31.

Memoli MJ, Shaw PA, Han A, Czajkowski L, Reed S, Athota R, et al. Evaluation of antihemagglutinin and antineuraminidase antibodies as correlates of protection in an influenza A/H1N1 virus healthy human challenge model. mBio. 2016;7(2):e00417-16.

Monto AS. Reflections on the Global Influenza Surveillance and Response System (GISRS) at 65 years: an expanding framework for influenza detection, prevention, and control. Influenza Other Respir Viruses. 2018;12(1):10-2.

Murphy BR, Kasel JA, Chanock RM. Association of serum anti-neuraminidase antibody with resistance to influenza in man. N Engl J Med. 1972;286(25):1329-32.

Polack FP, Thomas SJ, Kitchin N, Absalon J, Gurtman A, Lockhart S, et al; C4591001 Clinical Trial Group. Safety and efficacy of the BNT162b2 mRNA COVID-19 vaccine. N Engl J Med. 2020;383(27):2603-15.

Riedel S, Hobden JA, Miller SA, Morse SA, Mietzner TA, Detrick B, et al. Orthomyxoviruses (Influenza Viruses). In: Riedel S, Morse SA, Mietzner TA, Miller SA, editors. Jawetz, Melnick & Adelberg's Medical Microbiology. Twenty-eighth ed. New York: McGraw-Hill Education; 2019. p. 565-78.

Rockman S, Laurie KL, Parkes S, Wheatley A, Barr IG. New technologies for influenza vaccines. Microorganisms. 2020;8(11):1745.

Rüggeberg JU, Gold MS, Bayas JM, Blum MD, Bonhoeffer J, Friedlander S, et al; Brighton Collaboration Anaphylaxis Working Group. Anaphylaxis: case definition and guidelines for data collection, analysis, and presentation of immunization safety data. Vaccine. 2007;25(31):5675-84.

Shimabukuro TT, Cole M, Su JR. Reports of anaphylaxis after receipt of mRNA COVID-19 vaccines in the US—December 14, 2020-January 18, 2021. JAMA. 2021;325(11):1101-2.

Weiss CD, Wang W, Lu Y, Billings M, Eick-Cost A, Couzens L, et al. Neutralizing and neuraminidase antibodies correlate with protection against influenza during a late season A/H3N2 outbreak among unvaccinated military recruits. Clin Infect Dis. 2020;71(12):3096-102.

World Health Organization (WHO). Influenza (seasonal): Ask the expert: influenza Q&A [Internet]. 2018 Nov 6 (cited 2021 Aug 31). Available from: https://www.who.int/news-room/fact-sheets/detail/influenza-(seasonal).

Zent O, Arras-Reiter C, Broeker M, Hennig R. Immediate allergic reactions after vaccinations – a post-marketing surveillance review. Eur J Pediatr. 2002;161(1):21-5.

11. SUPPORTING DOCUMENTATION AND OPERATIONAL CONSIDERATIONS

11.1. APPENDIX 1: Study Governance Considerations

11.1.1. Regulatory and Ethical Considerations

This study will be conducted in accordance with the protocol and with the following:

- Consensus ethical principles derived from international guidelines, including the Declaration of Helsinki and Council for International Organizations of Medical Sciences International Ethical Guidelines.
- Applicable ICH GCP guidelines.
- Applicable laws and regulatory requirements.
- The protocol, protocol amendments, ICF, IB, and other relevant documents (eg, advertisements) must be submitted to an IRB by the Investigator and reviewed and approved by the IRB before the study is initiated.
- Any amendments to the protocol will require IRB approval before implementation of changes made to the study design, except for changes necessary to eliminate an immediate hazard to study participants.
- The Investigator will be responsible for the following:
  - Providing written summaries of the status of the study to the IRB annually or more frequently in accordance with the requirements, policies, and procedures established by the IRB.
  - Notifying the IRB of SAEs or other significant safety findings as required by IRB procedures.
  - Providing oversight of the conduct of the study at the site and adherence to requirements of 21 CFR, ICH GCP guidelines, the IRB, and all other applicable local regulations.

11.1.2. Study Monitoring

Before an investigational study site can enter a participant into the study, the Sponsor or its representatives will visit the study site for the following:

- Determine the adequacy of the facilities.
- Discuss with the Investigator(s) and other personnel their responsibilities with regard to protocol adherence, and the responsibilities of the Sponsor or its representatives. This will be documented in a Clinical Study Agreement between the Sponsor, the designated CRO, and the Investigator.

According to ICH GCP guidelines, the Sponsor of the study is responsible for ensuring the proper conduct of the study with regard to protocol adherence and validity of data recorded on the eCRFs. The study monitor's duties are to aid the Investigator and the Sponsor in the maintenance of complete, accurate, legible, well-organized, and easily retrievable data. The study monitor will advise the Investigator of the regulatory necessity for study-related monitoring, audits, IRB review, and inspection by providing direct access to the source data and/or documents. In addition, the study monitor will explain to and interpret for the Investigator all regulations applicable to the clinical evaluation of IP as documented in ICH guidelines.

It is the study monitor's responsibility to inspect the eCRFs and source documentation throughout the study to protect the rights of the participants; to verify adherence to the protocol; to verify completeness, accuracy, and consistency of the data; and to confirm adherence of study conduct to any local regulations. Details will be outlined in the clinical monitoring plan. During the study, a monitor from the Sponsor or a representative will have regular contacts with the study site, for the following purposes:

- Provide information and support to the Investigator(s).
- Confirm that facilities remain acceptable.
- Confirm that the investigational team is adhering to the protocol, that the data are being accurately recorded in the eCRFs, and that IP accountability checks are being performed.
- Perform source data verification. This includes a comparison of the data in the eCRFs with the participant's medical records at the hospital or practice and other records relevant to the study. This will require direct access to all original records for each participant (eg, clinical charts or electronic medical record system).
- Record and report any protocol deviations not previously sent.
- Confirm that AEs and SAEs have been properly documented on eCRFs, that any SAEs have been forwarded to the SAE Hotline, and that those SAEs that meet criteria for reporting have been forwarded to the IRB.

The monitor will be available between visits if the Investigator(s) or other staff needs information or advice.

11.1.3. Audits and Inspections

The Sponsor, their designee(s), the IRB, or regulatory authorities will be allowed to conduct site visits to the investigational facilities for the purpose of monitoring or inspecting any aspect of the study. The Investigator agrees to allow the Sponsor, their designee(s), the IRB, or regulatory

authorities to inspect the IP storage area, IP stocks, IP records, participant charts, and study source documents, and other records relative to study conduct.

Authorized representatives of the Sponsor, a regulatory authority, and the IRB may visit the study site to perform audits or inspections, including source data verification. The purpose of a Sponsor audit or inspection is to systematically and independently examine all study-related activities and documents to determine whether these activities were conducted and whether data were recorded, analyzed, and accurately reported according to the protocol, ICH GCP (R2), and any applicable regulatory requirements. The Investigator should contact the Sponsor immediately if contacted by a regulatory agency about an inspection.

The Principal Investigator must obtain IRB approval for the investigation. Initial IRB approval and all materials approved by the IRB for this study, including the participant ICF and recruitment materials must be maintained by the Investigator and made available for inspection.

11.1.4. Financial Disclosure

The Investigator is required to provide financial disclosure information to allow the Sponsor to submit the complete and accurate certification or disclosure statements required under 21 CFR 54. In addition, the Investigator must provide the Sponsor with a commitment to promptly update this information if any relevant changes occur during the course of the investigation and for 1 year following the completion of the study.

The Sponsor, the CRO, and the study site are not financially responsible for further testing or treatment of any medical condition that may be detected during the screening process. In addition, in the absence of specific arrangements, the Sponsor, the CRO, and the study site are not financially responsible for further treatment of the disease under study.

11.1.5. Recruitment Procedures

Advertisements to be used for the recruitment of study participants and any other written information regarding this study to be provided to the participant should be submitted to the Sponsor for approval. All documents must be approved by the IRB.

11.1.6. Informed Consent Process

The informed consent document(s) must meet the requirements of 21 CFR 50, local regulations, ICH guidelines, Health Insurance Portability and Accountability Act requirements, where applicable, and the IRB or study site. All consent documents will be approved by the appropriate IRB. The actual ICF used at each study site may differ, depending on local regulations and IRB requirements. However, all versions must contain the standard information found in the sample

ICF provided by the Sponsor. Any change to the content of the ICF must be approved by the Sponsor and the IRB prior to the form being used.

If new information becomes available that may be relevant to the participant's willingness to continue participation in the study, this will be communicated to him/her in a timely manner. Such information will be provided via a revised ICF or an addendum to the original ICF.

The Investigator or his/her representative will explain the nature of the study to the participant and answer all questions regarding the study.

The Investigator is responsible for ensuring that the participant fully understands the nature and purpose of the study. Information should be given in both oral and written form whenever possible.

No participant should be obliged to participate in the study. The participant must be informed that participation is voluntary. Participants, their relatives, guardians, or (if applicable) legal representatives must be given ample opportunity to inquire about details of the study. The information must make clear that refusal to participate in the study or withdrawal from the study at any stage is without any prejudice to the participant's subsequent care.

The participant must be allowed sufficient time to decide whether they wish to participate.

The participant must be made aware of and give consent to direct access to his/her source medical records by study monitors, auditors, the IRB, and regulatory authorities. The participant should be informed that such access will not violate participant confidentiality or any applicable regulations. The participant should also be informed that he/she is authorizing such access by signing the ICF.

A copy of the ICF(s) must be provided to the participant.

A participant who is rescreened is not required to sign another ICF if the rescreening occurs within 28 days from the previous ICF signature date (within the initial screening period).

The ICF will also explain that excess serum from immunogenicity testing may be used for future research, which may be performed at the discretion of the Sponsor to further characterize the immune response to influenza viruses, for additional assay development, and to examine the immune response across influenza viruses.

11.1.7. Protocol Amendments

No change or amendment to this protocol may be made by the Investigator or the Sponsor after the protocol has been agreed to and signed by all parties unless such change(s) or amendment(s) has (have) been agreed upon by the Investigator or the Sponsor. Any change agreed upon will be recorded in writing, and the written amendment will be signed by the Investigator and the

Sponsor. The IRB approval is required prior to the implementation of an amendment, unless overriding safety reasons warrant immediate action, in which case the IRB(s) will be promptly notified.

Any modifications to the protocol or the ICF that may impact the conduct of the study or the potential benefit of the study or may affect participant safety, including changes of study objectives, study design, participant population, sample sizes, study procedures, or significant administrative aspects will require a formal amendment to the protocol. Such amendment will be released by the Sponsor, agreed to by the Investigator(s), and approved by the relevant IRB(s) prior to implementation. A signed and dated statement that the protocol, any subsequent relevant amended documents, and the ICF have been approved by relevant IRB(s) must be provided to the Sponsor before the study is initiated.

Administrative changes to the protocol are minor corrections and/or clarifications that have no effect on the way the study is to be conducted. These administrative changes will be released by the Sponsor and agreed to by the Investigators, and the IRB(s) will be notified.

11.1.8. Protocol Deviations

Noncompliance may be on the part of the participant, the Investigator, or the study site staff. As a result of deviations, corrective actions are to be developed by the site and implemented promptly.

It is the responsibility of the Site Investigator to use continuous vigilance to identify and report deviations to the Sponsor or its designee in a timely manner. All deviations must be addressed in study source documents and reported to the study monitor. Protocol deviations must be sent to the reviewing IRB per their policies. The Site Investigator is responsible for knowing and adhering to the reviewing IRB requirements.

11.1.9. Data Protection

Participants will be assigned a unique identifier by the Sponsor. Any participant records or datasets that are transferred to the Sponsor will contain the identifier only; participant names or any information that would make the participant identifiable will not be transferred.

The participant must be informed that his/her personal study-related data will be used by the Sponsor in accordance with local data protection law. The level of disclosure must also be explained to the participant.

The participant must be informed that his/her medical records may be examined by Clinical Quality Assurance (QA) auditors or other authorized personnel appointed by the Sponsor, by appropriate IRB members, and by inspectors from regulatory authorities.

Individual participant medical information obtained as a result of this study is considered confidential, and disclosure to third parties is prohibited. Information will be accessible to authorized parties or personnel only. Medical information may be given to the participant's physician or to other appropriate medical personnel responsible for the participant's well-being. Each participant will be asked to complete a form allowing the Investigator to notify the participant's primary HCP of his/her participation in this study.

All laboratory specimens, evaluation forms, reports, and other records will be identified in a manner designed to maintain participant confidentiality. All records will be kept in a secure storage area with limited access. Clinical information will not be released without the written permission of the participant, except as necessary for monitoring and auditing by the Sponsor, its designee, the relevant regulatory authority, or the IRB.

The Investigator and all employees and coworkers involved with this study may not disclose or use for any purpose other than performance of the study, any data, record, or other unpublished, confidential information disclosed to those individuals for the purpose of the study. Prior written agreement from the Sponsor or its designee must be obtained for the disclosure of any confidential information to other parties.

11.1.10. Sample Retention and Future Biomedical Research

The retention period of laboratory samples will be 20 years, or as permitted by local regulations, to address further scientific questions related to mRNA-1020, mRNA-1030, mRNA-1010, or anti-respiratory virus immune response. In addition, identifiable samples can be destroyed at any time at the request of the participant. During the study or during the retention period, in addition to the analysis outlined in the study endpoints, exploratory analysis may be conducted using other measures of adaptive immunity to seasonal influenza to include humoral and cellular immune assay methodologies on any remaining blood or serum samples, including samples from participants who are screened but are not subsequently enrolled. These analyses may extend the search for other potentially relevant biomarkers to investigate the effect of mRNA-1020, mRNA-1030, and mRNA-1010 as well as to determine how changes in biomarkers may relate to exposure and clinical outcomes. A decision to perform such exploratory research may arise from new scientific findings related to the drug/vaccine class or disease, as well as reagent and assay availability.

11.1.11. Safety Oversight

Safety monitoring for this study will include the blinded study team members, inclusive of, at a minimum, the Sponsor's medical monitor, CRO's medical monitor, a blinded IST, and an unblinded DSMB. The study team will conduct ongoing blinded safety reviews during the study

and will be responsible for notifying the IST and DSMB of potential safety signal events or the triggering of pause rules.

The IST will consist of at least 2 Sponsor physicians who will be voting members not directly involved in the clinical development of the study. The IST will conduct a blinded review of all available safety data after approximately 96 participants (approximately 12 participants per study arm) have completed their Day 8 Visit. Enrollment will continue while this review is conducted if no pause rules have been met and the study team has not identified any safety concerns. Finally, the IST will also conduct ad hoc reviews as requested by the study medical monitor and the study team.

An independent unblinded DSMB will be used throughout the conduct of this study. This committee will be composed of independent members with relevant therapeutic and/or biostatistical expertise to allow for the ongoing unblinded review of safety data from this study population. Safety data will be reviewed according to intervals defined in the DSMB charter and will also occur as needed when study stopping or pausing criteria are met, or as otherwise requested by the study team and/or IST.

Details regarding the IST and DSMB composition, responsibilities, procedures, and frequency of data review will be defined in their respective charters.

11.1.12. Dissemination of Clinical Study Data

The Sponsor shares information about clinical studies and results on publicly accessible websites, based on international and local legal and regulatory requirements, and other clinical study disclosure commitments established by pharmaceutical industry associations. These websites include clinicaltrials.gov, European Union clinical trial register (eu.ctr), and some national registries.

11.1.13. Data Quality Assurance and Quality Control

Data collection is the responsibility of the clinical study staff at the site under the supervision of the Site Investigator. The Investigator is responsible for ensuring the accuracy, completeness, legibility, and timeliness of the data reported.

- All participant data relating to the study will be recorded in the eCRF unless transmitted to the Sponsor or designee electronically (eg, laboratory data). The Investigator is responsible for verifying that data entries are accurate and correct by physically or electronically signing the eCRF.
- The Investigator must maintain accurate documentation (source data) that supports the information entered in the eCRF.

- The Investigator must permit study-related monitoring, audits, IRB review, and regulatory agency inspections and provide direct access to source data documents.
- Monitoring details describing strategy (eg, risk-based initiatives in operations and quality such as risk management and mitigation strategies and analytical risk-based monitoring), methods, responsibilities and requirements, including handling of noncompliance issues and monitoring techniques (central, remote, or onsite monitoring) are provided in the clinical monitoring plan.
- The Sponsor or designee is responsible for the data management of this study, including quality checking of the data.
- The Sponsor assumes accountability for actions delegated to other individuals (eg, CROs).
- Study monitors will perform ongoing source data verification to confirm that data entered into the eCRF by authorized site personnel are accurate, complete, and verifiable from source documents; that the safety and rights of participants are being protected; and that the study is being conducted in accordance with the currently approved protocol and any other study agreements, ICH GCP, and all applicable regulatory requirements.
- Records and documents, including signed ICFs, pertaining to the conduct of this study must be retained by the Investigator for a period of at least 2 years after the last marketing application approval or, if not approved, 2 years following the discontinuance of the test article for investigation. If this requirement differs from any local regulations, the local regulations will take precedence unless the local retention policy is less than 2 years. No records may be destroyed during the retention period without the written approval of the Sponsor. No records may be transferred to another location or party without written notification to the Sponsor.

Quality assurance includes all the planned and systematic actions that are established to ensure that the clinical study is performed, and the data are generated, documented (recorded), and reported according to ICH GCP and local/regional regulatory standards.

A QA representative from the Sponsor or qualified designee, who is independent of and separated from routine monitoring, may periodically arrange inspections/audits of the clinical study by reviewing the data obtained and procedural aspects. These inspections may include onsite inspections/audits and source data checks. Direct access to source documents is required for the purpose of these periodic inspections/audits.

11.1.14. Data Collection and Management

This study will be conducted in compliance with ICH GCP guidelines. This study will also be conducted in accordance with the most recent version of the Declaration of Helsinki.

This study will use electronic data collection to collect data directly from the study site using eCRFs. The Investigator is responsible for ensuring that all sections of each eCRF are completed promptly and correctly and that entries can be verified against any source data.

Study monitors will perform source document verification to identify inconsistencies between the eCRFs and source documents. Discrepancies will be resolved in accordance with the principles of GCP. Detailed study monitoring procedures are provided in the clinical monitoring plan.

All AEs will be coded with MedDRA. Concomitant medications will be coded using WHODrug Global.

11.1.15. Source Documents

Source documents are original documents or certified copies and include, but are not limited to, eDiaries, medical and hospital records, screening logs, ICFs, telephone contact logs, and worksheets. Source documents provide evidence for the existence of the participant and substantiate the integrity of the data collected. Source documents are filed at the Investigator's site.

Data reported on the case report form or entered in the eCRF that are transcribed from source documents must be consistent with the source documents or the discrepancies must be explained. The Investigator may need to request previous medical records or transfer records, depending on the study. Also, current medical records must be available.

The Sponsor or its designee requires that the Investigator prepare and maintain adequate and accurate records for each participant treated with the IP. Source documents such as any hospital, study site, or office charts and the signed ICFs are to be included in the Investigator's files with the participant's study records.

11.1.16. Retention of Records

The Principal Investigator must maintain all documentation relating to the study for a period of at least 2 years after the last marketing application approval or, if not approved, 2 years following the discontinuance of the test article for investigation. If this requirement differs from any local regulations, the local regulations will take precedence unless the local retention policy is less than 2 years.

If it becomes necessary for the Sponsor or the regulatory authority to review any documentation relating to the study, the Investigator must permit access to such records. No records will be destroyed without the written consent of the Sponsor, if applicable. It is the responsibility of the Sponsor to inform the Investigator when these documents no longer need to be retained.

11.1.17. Study and Site Closure

If the study is prematurely terminated or suspended, the Sponsor shall promptly inform the Investigators, the IRBs, the regulatory authorities, and any CRO(s) used in the study of the reason for termination or suspension, as specified by the applicable regulatory requirements. The Investigator shall promptly inform the participant and should ensure appropriate participant therapy and/or follow-up.

The Sponsor or designee reserves the right to close the study site or terminate the study at any time for any reason at the sole discretion of the Sponsor.

The Investigator may initiate study site closure at any time, provided there is reasonable cause and sufficient notice is given in advance of the intended termination.

Reasons for the early closure of a study site by the Sponsor or Investigator may include but are not limited to the following:

- Continuation of the study represents a significant medical risk to participants.
- Failure of the Investigator to comply with the protocol, the requirements of the IRB or local health authorities, the Sponsor's procedures, or ICH GCP guidelines.
- Inadequate recruitment of participants by the Investigator.
- Discontinuation of further mRNA-1020 or mRNA-1030 development.

Study sites will be closed upon study completion. A study site is considered closed when all required documents and study supplies have been collected and a study site closure visit has been performed.

11.1.18. Publication Policy

The results of this study may be published or presented at scientific meetings. If this is foreseen, the Investigator agrees to submit all manuscripts or abstracts to the Sponsor before submission. This allows the Sponsor to protect proprietary information and to provide comments.

The Sponsor will comply with the requirements for publication of study results. In accordance with standard editorial and ethical practice, the Sponsor will generally support publication of multicenter studies only in their entirety and not as individual site data. In this case, a Coordinating Investigator will be designated by mutual agreement.

Authorship will be determined by mutual agreement and in line with International Committee of Medical Journal Editors authorship requirements.

The clinical study plan and the results of the study will be published on www.ClinicalTrials.gov in accordance with 21 CFR 50.25(c). The results of and data from this study belong to the Sponsor.

11.2. APPENDIX 2: Contraceptive Guidance

Definitions:

Woman of Childbearing Potential (WOCBP):

Women in the following categories are considered WOCBP (fertile):

1. Following menarche
2. From the time of menarche until becoming postmenopausal unless permanently sterile (see below)
    - A postmenopausal state is defined as no menses for 12 months without an alternative medical cause.
        - A high follicle-stimulating hormone (FSH) level in the postmenopausal range may be used to confirm a postmenopausal state in women not using hormonal contraception or hormonal replacement therapy (HRT). However, in the absence of 12 months of amenorrhea, confirmation with more than one FSH measurement is required.
        - Females on HRT and whose menopausal status is in doubt will be required to use one of the nonestrogen hormonal highly effective contraception methods if they wish to continue their HRT during the study. Otherwise, they must discontinue HRT to allow confirmation of postmenopausal status before study enrollment.
    - Permanent sterilization methods (for the purpose of this study) include:
        - Documented hysterectomy
        - Documented bilateral salpingectomy
        - Documented bilateral oophorectomy
        - Documented tubal ligation
        - For individuals with permanent infertility due to an alternate medical cause other than the above, (eg, Müllerian agenesis, androgen insensitivity, gonadal dysgenesis), investigator discretion should be applied to determining study entry.
    - If fertility is unclear (eg, amenorrhea in adolescents or athletes) and a menstrual cycle cannot be confirmed before first dose of study intervention, additional evaluation should be considered.

Note: Documentation can come from the site personnel's review of the participant's medical records, medical examination, or medical history interview.

Contraception Guidance:

Adequate female contraception is defined as consistent and correct use of an FDA-approved contraceptive method in accordance with the product label. The following are examples:

- Barrier method (such as condoms, diaphragm, or cervical cap) used in conjunction with spermicide.
- Intrauterine device.
- Prescription hormonal contraceptive taken or administered via oral (pill), transdermal (patch), subdermal, or IM route.
- Sterilization of a female participant's monogamous male partner prior to entry into the study.

Note that periodic abstinence (eg, calendar, ovulation, symptothermal, postovulation methods) and withdrawal are not acceptable methods of contraception.

11.3. APPENDIX 3: Adverse Events of Special Interest Terms

Investigators should report all AEs, which fall into the following categories presented in Table 10 as an AESI per the reporting processes specified in Section 8.4.13. The following AESIs are medical concepts that are generally of interest in vaccine safety surveillance per the Brighton Collaboration and Safety Platform for Emergency Vaccines.

Table 10: Adverse Events of Special Interest

Adverse Event of Special Interest	Notes
Thrombocytopenia	• Platelet counts < 150 x 10^9<br>• Including but not limited to immune thrombocytopenia, platelet production decreased, thrombocytopenia, thrombocytopenic purpura, thrombotic thrombocytopenic purpura, or HELLP syndrome
New onset of or worsening of the following neurologic diseases	• Guillain-Barre Syndrome<br>• Acute disseminated encephalomyelitis<br>• Idiopathic peripheral facial nerve palsy (Bell's palsy)<br>• Seizures including, but not limited to, febrile seizures and/or generalized seizures/convulsions
Anaphylaxis	• Anaphylaxis as defined in Section 8.4.6<br>• Following reporting procedures as specified in Section 8.4.13
Myocarditis/Pericarditis	• Myocarditis<br>• Pericarditis<br>• Myopericarditis

Abbreviation: HELLP = hemolysis, elevated liver enzymes, low platelet count.

11.4. APPENDIX 4: CDC Working Case Definition of Pericarditis, Myocarditis, and Myopericarditis Occurring After Receipt of COVID-19 mRNA Vaccines

Table 11: Case Definitions of Probable and Confirmed Myocarditis, Pericarditis, and Myopericarditis

Condition	Definition	
**Acute Myocarditis**	**Probable Case**	**Confirmed Case**
	Presence of ≥1 new or worsening of the following clinical symptoms:*	Presence of ≥1 new or worsening of the following clinical symptoms:*
	• chest pain, pressure, or discomfort	• chest pain, pressure, or discomfort
	• dyspnea, shortness of breath, or pain with breathing	• dyspnea, shortness of breath, or pain with breathing
	• palpitations	• palpitations
	• syncope	• syncope
	**OR,** infants and children aged <12 years might instead have ≥2 of the following symptoms:	**OR,** infants and children aged <12 years might instead have ≥2 of the following symptoms:
	• irritability	• irritability
	• vomiting	• vomiting
	• poor feeding	• poor feeding
	• tachypnea	• tachypnea
	• lethargy	• lethargy
	**AND**	**AND**
	≥1 new finding of	≥1 new finding of
	• troponin level above upper limit of normal (any type of troponin)	• Histopathologic confirmation of myocarditis†
	• abnormal electrocardiogram (ECG or EKG) or rhythm monitoring findings consistent with myocarditis§	
	• abnormal cardiac function or wall motion abnormalities on echocardiogram	• cMRI findings consistent with myocarditis in the presence of troponin level above upper limit of normal (any type of troponin)
	• cMRI findings consistent with myocarditis	
	**AND**	**AND**
	• No other identifiable cause of the symptoms and findings	• No other identifiable cause of the symptoms and findings

Condition	Definition
**Acute pericarditis****	Presence of ≥2 new or worsening of the following clinical features:
	• acute chest pain††
	• pericardial rub on exam
	• new ST-elevation or PR-depression on EKG
	• new or worsening pericardial effusion on echocardiogram or MRI
**Myopericarditis**	This term may be used for patients who meet criteria for both myocarditis and pericarditis.

Abbreviations: AV = atrioventricular; cMRI = cardiac magnetic resonance imaging; ECG or EKG = electrocardiogram.

Note: An independent Cardiac Event Adjudication Committee (CEAC) comprised of medically qualified personnel, including cardiologists, will review suspected cases of myocarditis, pericarditis, and myopericarditis to determine if they meet Center for Disease Control and Prevention criteria for "probable" or "confirmed" events, (Gargano et al 2021), and provide the assessment to the Sponsor. The CEAC members will be blinded to study treatment. Details regarding the CEAC composition, responsibilities, procedures, and frequency of data review will be defined in the CEAC charter.

* Persons who lack the listed symptoms but who meet other criteria may be classified as subclinical myocarditis (probable or confirmed).

† Using the Dallas criteria (Aretz et al 1987). Autopsy cases may be classified as confirmed clinical myocarditis on the basis of meeting histopathologic criteria if no other identifiable cause.

§ To meet the ECG or rhythm monitoring criterion, a probable case must include at least one of 1) ST-segment or T-wave abnormalities; 2) Paroxysmal or sustained atrial, supraventricular, or ventricular arrhythmias; or 3) AV nodal conduction delays or intraventricular conduction defects.
Using either the original or the revised Lake Louise criteria.
https://www.sciencedirect.com/science/article/pii/S0735109718388430?via%3Dihubexternal icon

** https://academic.oup.com/eurheartj/article/36/42/2921/2293375external icon

†† Typically described as pain made worse by lying down, deep inspiration, or cough, and relieved by sitting up or leaning forward, although other types of chest pain might occur.

Reference: (Gargano et al 2021).

Signature Page for VV-CLIN-004501 v2.0

Approval	PPD<br>16-Feb-2022 15:21:24 GMT+0000

Signature Page for VV-CLIN-004501 v2.0

ModernaTX, Inc.

Protocol mRNA-1020-P101

A Phase 1/2, Randomized, Observer-Blind, Dose Ranging Study to Evaluate the Safety, Reactogenicity, and Immunogenicity of mRNA-1020 and mRNA-1030 Candidate Seasonal Influenza Vaccines in Healthy Adults

STATISTICAL ANALYSIS PLAN

SAP Version: 2.0
Version Date of SAP: 12-Jul-2022

Prepared by:

Parexel International
275 Grove Street
Suite 101C,
Newton, MA 02466

SIGNATURE PAGE

Prepared by Parexel:

PPD

Parexel International

Approved by Moderna:

PPD

ModernaTX, Inc

PPD

ModernaTX, Inc

PPD

ModernaTX, Inc

PPD

DOCUMENT HISTORY

Version	Date	Description of main modifications
1.0	29-March-2022	Initial Version
2.0	12-July-2022	• Added between-group comparison for GMTR using ANCOVA.<br>• Added between-group SCR difference using Miettinen-Nurminen method.<br>• Changed D29 visit window for PP Set to -7/+14 days<br>• Changed the ‘Analysis Visit Window’ table.<br>• Updated Medical Lead team member

DocuSign Envelope ID: 264FC66D-43E3-471B-863F-16B5EDBC3D09

TABLE OF CONTENTS

LIST OF ABBREVIATIONS 6
1 INTRODUCTION 8
2 STUDY OBJECTIVES........ 8
2.1 PRIMARY OBJECTIVES 8
2.2 SECONDARY OBJECTIVES 8
2.3 EXPLORATORY OBJECTIVES 9
3 STUDY ENDPOINTS........ 9
3.1 PRIMARY ENDPOINTS 9
3.2 SECONDARY ENDPOINTS 10
3.3 EXPLORATORY ENDPOINTS........ 10
4 STUDY DESIGN........ 10
4.1 OVERALL STUDY DESIGN 10
4.2 STATISTICAL HYPOTHESES 13
4.3 SAMPLE SIZE AND POWER 14
4.4 RANDOMIZATION........ 14
4.5 BLINDING AND UNBLINDING 14
5 ANALYSIS POPULATIONS 14
5.1 RANDOMIZATION SET........ 14
5.2 FULL ANALYSIS SET (FAS) 15
5.3 PER-PROTOCOL (PP) SET........ 15
5.4 SAFETY SET........ 17
5.5 SOLICITED SAFETY SET 17
6 STATISTICAL ANALYSIS 18
6.1 PLANNED ANALYSES 18
6.1.1 Interim Analysis (IA)........ 18
6.1.2 Internal Safety Team (IST) Review 18
6.1.3 Data Safety Monitoring Board (DSMB) Review........ 18
6.1.4 Final Analysis 18
6.2 GENERAL CONSIDERATIONS 18
6.2.1 Data Precision 19
6.2.2 Statistics for Continuous and Categorical Variables 19
6.2.3 Baseline........ 19
6.2.4 Reference Start Date and Study Day 19
6.2.5 Handling Non-Quantifiable Antibody Values........ 20
6.2.6 Analysis Periods for Safety Analyses........ 20
6.2.7 Unscheduled Visits and Visit Windowing Rules 20
6.2.8 Handling Missing and Incomplete Data 21

DocuSign Envelope ID: 264FC66D-43E3-471B-863F-16B5EDBC3D09

6.2.9 Age and Vaccination Groups *21*
6.2.10 Adjustment for Covariates *22*
6.3 BACKGROUND CHARACTERISTICS 22
6.3.1 Participant Disposition *22*
6.3.2 Demographics *23*
6.3.3 Medical History *24*
6.3.4 Prior and Concomitant Medications *24*
6.3.5 Study Exposure *25*
6.3.6 Major Protocol Deviations *25*
6.3.7 COVID-19 Impact *25*
6.4 SAFETY ANALYSIS 26
6.4.1 Unsolicited Adverse Events *26*
6.4.1.1 Incidence of Unsolicited Adverse Events 27
6.4.1.2 TEAEs by System Organ Class and Preferred Term 28
6.4.1.3 TEAEs by Preferred Term 28
6.4.1.4 TEAEs by System Organ Class, Preferred Term, and Severity 29
6.4.2 Serious Adverse Events *29*
6.4.3 Solicited Adverse Reactions *29*
6.4.4 Clinical Laboratory Evaluations *31*
6.4.5 Vital Sign Measurements *31*
6.4.6 Physical Examinations *32*
6.4.7 Electrocardiogram (ECG) *32*
6.5 IMMUNOGENICITY ANALYSIS 32
6.5.1 Immunogenicity Assessments *34*
6.5.2 Primary Analysis of Immunogenicity *35*
6.5.3 Secondary Analysis of Immunogenicity *36*
6.5.4 Exploratory Analysis of Immunogenicity *38*
6.6 ANALYSIS OF INFLUENZA INFECTION 39
6.7 INTERIM ANALYSIS 39
6.8 INTERNAL SAFETY TEAM REVIEW 40
6.9 DATA SAFETY MONITORING BOARD REVIEW 40
6.10 SUBGROUP ANALYSIS 41
7 CHANGES FROM PLANNED ANALYSES IN PROTOCOL 41
8 REFERENCES 41
9 LIST OF APPENDICES 42
9.1 *APPENDIX A STANDARDS FOR SAFETY AND IMMUNOGENICITY VARIABLE DISPLAY IN TLFS* 42
9.2 *APPENDIX B ANALYSIS VISIT WINDOWS FOR SAFETY AND IMMUNOGENICITY ANALYSIS* 42
9.3 *APPENDIX C IMPUTATION RULES FOR MISSING PRIOR/CONCOMITANT MEDICATIONS AND NON-STUDY VACCINATIONS DATES* 43
9.4 *APPENDIX D IMPUTATION RULES FOR MISSING AE DATES* 44
9.5 *APPENDIX E SEVERITY GRADING OF LABORATORY ABNORMALITIES* 45
9.6 *APPENDIX F SEVERITY GRADING OF VITAL SIGN ABNORMALITIES* 47

LIST OF ABBREVIATIONS

Abbreviation	Definition
AE	adverse event
AESI	adverse event of special interest
ANCOVA	analysis of covariance
AR	adverse reaction
BMI	body mass index
BUN	blood urea nitrogen
BP	blood pressure
CDC	United States Centers for Disease Control and Prevention
CI	confidence interval
COVID-19	coronavirus disease 2019
CRO	contract research organization
CSP	clinical study protocol
CSR	clinical study report
DBP	data blinding plan
DHHS	Department of Health and Human Services
DSMB	Data Safety Monitoring Board
ECG	Electrocardiogram
eCRF	electronic case report form
eDiary	electronic diary
EoS	end of study
FAS	Full Analysis Set
Fc	fragment crystallizable
FDA	Food and Drug Administration
FSH	follicle stimulating hormone
GM	geometric mean
GMFR	geometric mean fold rise
GMT	geometric mean titer
HA	hemagglutinin
HAI	hemagglutination inhibition
IA	interim analysis
ILI	influenza-like illness
IP	investigational product
IST	internal safety team
LLOQ	lower limit of quantification
MAAE	medically attended adverse event
max	maximum
MedDRA	Medical Dictionary for Regulatory Activities
min	minimum
MN	microneutralization
mRNA	messenger ribonucleic acid
NA	neuraminidase
NAI	neuraminidase inhibition

Abbreviation	Definition
NH	Northern Hemisphere
NP	nasopharyngeal
PP	per-protocol
PT	preferred term
RT-PCR	reverse transcriptase polymerase chain reaction
SAE	serious adverse event
SAP	statistical analysis plan
SARS-CoV-2	Severe acute respiratory syndrome coronavirus 2
SAS	Statistical Analysis System
SD	standard deviation
SoE	schedule of events
SOC	system organ class
TEAE	treatment-emergent adverse event
ULOQ	upper limit of quantification
WHO	World Health Organization
WHO Drug Global	World Health Organization drug dictionary

DocuSign Envelope ID: 264FC66D-43E3-471B-863F-16B5EDBC3D09

1 INTRODUCTION

This statistical analysis plan (SAP), which describes the planned analyses for Study mRNA-1020-P101, is based on the protocol dated 16-Feb-2022, and the electronic case report form (eCRF), dated 21-Mar-2022. SAP will be based on the latest protocol and eCRF and will be updated as necessary.

In addition to the information presented in the SAP section of the protocol (Section 9), which provides the principal features of analyses for this study, this SAP provides statistical analysis details/data derivations.

This is a Phase 1/2, randomized, observer-blind, dose-ranging study to evaluate the safety, reactogenicity, and immunogenicity of mRNA-1020 and mRNA-1030 candidate seasonal influenza vaccines in healthy adults 18 to 75 years of age.

Parexel Biostatistics and Statistical Programming team, designee of Moderna Biostatistics and Statistical Programming department, will perform the statistical analysis of the safety, reactogenicity, and immunogenicity data; Statistical Analysis System (SAS) Version 9.4 or higher will be used to generate all statistical outputs (tables, figures, listings, and datasets). The SAP will be finalized and approved prior to the clinical database lock and treatment unblinding for the study. If the methods in this SAP differ from the methods described in the protocol, the SAP will prevail.

In this document, study vaccination, injection of investigational product (IP) / investigational vaccine, and injection are used interchangeably.

2 STUDY OBJECTIVES

2.1 Primary Objectives

- To evaluate the safety and reactogenicity of mRNA-1020, mRNA-1030, and mRNA-1010
- To evaluate the humoral immunogenicity of mRNA-1020, mRNA-1030, and mRNA-1010 against vaccine-matched influenza A and B strains at Day 29

2.2 Secondary Objectives

- To evaluate the humoral immunogenicity of mRNA-1020, mRNA-1030, and mRNA-1010 against vaccine-matched influenza A and B strains at all evaluable humoral immunogenicity time points

2.3 Exploratory Objectives

The exploratory objectives (may be performed) are the following:

- To evaluate the humoral immunogenicity against vaccine-mismatched influenza A and B strains
- To evaluate cellular immunogenicity in a subset of participants
- To further characterize antibody responses, for example, Fc-mediated function, avidity, or epitope specificity
- To assess the occurrence of clinical influenza in study participants and characterize their immune response to infection and viral isolates
- Sample collection to perform passive transfer studies in preclinical animal models

3 STUDY ENDPOINTS

3.1 Primary Endpoints

Safety

- Frequency and grade of each solicited local and systemic reactogenicity adverse reaction (AR) during a 7-day follow-up period post-vaccination
- Frequency and severity of any unsolicited adverse events (AEs) during the 28-day follow-up period post-vaccination
- Frequency of any serious adverse events (SAEs), adverse events of special interest (AESIs), medically attended adverse events (MAAEs), and AEs leading to withdrawal from Day 1 through Day 181/end of study (EoS)
- Safety laboratory abnormalities through 7 days post-vaccination

Immunogenicity

- Geometric mean titer (GMT) and geometric mean fold rise (GMFR) at Day 29 compared with Day 1 (baseline) and percentage of participants with seroconversion, defined as a Day 29 titer ≥ 1:40 if baseline is < 1:10 or a 4-fold or greater rise if baseline is ≥ 1:10 in anti-hemagglutinin (HA) antibodies measured by hemagglutination inhibition (HAI) assay
- GMT and GMFR of anti-neuraminidase (NA) measured by neuraminidase inhibition (NAI) assay at Day 1 and Day 29 and percentage of participants with a change in the Day 29 titer of at least 2-/3-/4-fold rise, defined as ≥ 2-/3-/4-fold of

the lower limit of quantification (LLOQ) if the Day 1 titer is < LLOQ; or ≥ 2-/3-/4-fold of the Day 1 titer if the Day 1 titer is ≥ LLOQ

3.2 Secondary Endpoints

The secondary objective will be evaluated by the following endpoints:

- GMT and GMFR of anti-HA or anti-NA antibodies as measured by HAI, NAI, and/or MN assays at all evaluable humoral immunogenicity time points compared with Day 1 (baseline)

3.3 Exploratory Endpoints

The exploratory endpoints are as follows:

- GMT and GMFR of anti-HA or anti-NA antibodies as measured by HAI, NAI, and/or MN assays against vaccine-mismatched strains compared with Day 1 (baseline)
- Frequency, magnitude, and phenotype of virus-specific T-cell and B-cell responses measured by flow cytometry or other methods, and to perform targeted repertoire analysis of T-cells and B-cells after vaccination
- Frequency, specificities, or other endpoints to be determined, for the further characterization of antibody responses
- Frequency of clinical influenza and assessment of immune responses in participants with clinical influenza
- Transfer of human sera into mice with subsequent influenza virus challenge to observe protection from morbidity and mortality conferred by NA-specific antibodies

4 STUDY DESIGN

4.1 Overall Study Design

This is a Phase 1/2, randomized, observer-blind, dose-ranging study to evaluate the safety, reactogenicity, and immunogenicity of mRNA-1020 (4 HA and 4 NA at 1:1 HA:NA mass ratio) and mRNA-1030 (4 HA and 4 NA at 3:1 HA:NA mass ratio) in healthy adults 18 to 75 years of age. Enrollment in study arms will proceed in parallel with randomization of all dose levels of mRNA-1020 and mRNA-1030 (Study Arms 1 to 6), the mRNA-1010

Comparator Arm (Study Arm 7), and the Flublok Comparator Arm (Study Arm 8). The study will randomize approximately 70 healthy adult participants into each study arm with a balanced age group representation stratified approximately 1:1 into age groups of 18 to < 50 years of age and ≥ 50 to ≤ 75 years of age.

The vaccines to be tested contain mRNAs encoding for the surface glycoproteins of the strains recommended by the WHO for 2021/22 NH cell- or recombinant-based vaccines:

- A/Wisconsin/588/2019(H1N1)pdm09
- A/Cambodia/e0826360/2020(H3N2)
- B/Washington/02/2019 (B/Victoria lineage)
- B/Phuket/3073/2013 (B/Yamagata lineage)

All participants will participate in a screening period (up to 28 days before Day 1), treatment period (single dose of vaccine on Day 1), and follow-up period (up to 6 months after vaccination). Approximately 560 participants will be randomized to receive a single dose of either one of the 2 next-generation candidate vaccines (mRNA-1020 or mRNA-1030) at different dose levels, a single dose of the first-generation candidate vaccine (mRNA-1010), or Flublok, a licensed enhanced seasonal influenza vaccine comparator (8 study arms total). Three different dose levels (50, 100, and 150 µg total mRNA) of the 1:1 HA:NA mass ratio vaccine (mRNA-1020), 3 different dose levels (25, 50, and 100 µg total mRNA) of the 3:1 HA:NA mass ratio vaccine (mRNA-1030), and a single dose level of mRNA-1010 (50 µg total mRNA) will be assessed. The target number of participants in each arm and the randomization ratio can be found in Table 1. The study schema is illustrated in Figure 1.

This study is designed as an observer-blind study. Participants in Vaccination Groups 1, 3, and 5 will receive mRNA-1030 vaccine and participants in Groups 2, 4, and 6 will receive mRNA-1020 vaccine. mRNA-1010 and Flublok will be used as comparators for descriptive comparisons of safety and immunogenicity.

Table 1 Study Arms and Dose Levels

Vaccination Group	Group Name	Antigen(s)	Total Dose	N (total)	Age 18-49	Age 50-75
1	**mRNA-1030**	HA+NA	**25 µg**	70	35	35
2	**mRNA-1020**	HA+NA	**50 µg**	70	35	35
3	**mRNA-1030**	HA+NA	**50 µg**	70	35	35
4	**mRNA-1020**	HA+NA	**100 µg**	70	35	35
5	**mRNA-1030**	HA+NA	**100 µg**	70	35	35
6	**mRNA-1020**	HA+NA	**150 µg**	70	35	35
7	**mRNA-1010** (HA only comparator)	HA only	**50 µg**	70	35	35
8	**Flublok** (active comparator)	HA only	**180 µg**	70	35	35
Total				560	280	280

Abbreviations: HA = hemagglutinin; mRNA = messenger RNA; NA = neuraminidase.

DocuSign Envelope ID: 264FC66D-43E3-471B-863F-16B5EDBC3D09

Figure 1 Study Schema

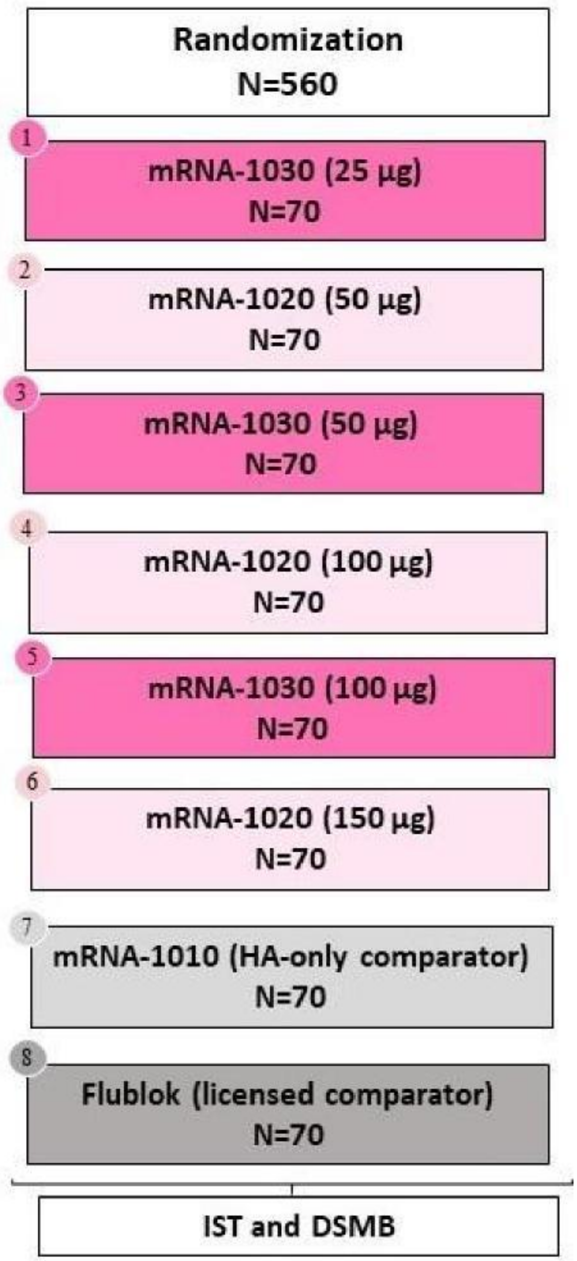

Abbreviations: DSMB = data safety monitoring board; HA = hemagglutinin; IST = internal safety team; V = visit.

4.2 Statistical Hypotheses

No formal hypotheses will be tested for this study.

DocuSign Envelope ID: 264FC66D-43E3-471B-863F-16B5EDBC3D09

4.3 Sample Size and Power

The sample size for this trial is not driven by statistical assumptions for formal hypothesis testing. The number of proposed participants is considered sufficient to provide a descriptive summary of the safety and immunogenicity of different dose levels of mRNA-1020 or mRNA-1030.

A total of approximately 560 participants will be randomly assigned to receive mRNA-1020, mRNA-1030, mRNA-1010, or Flublok. Among those ~560 participants, 70 participants will be randomized into each vaccination group. Details regarding the number of participants in each vaccination group and the randomization ratio are presented in Table 1. With 70 participants in each group receiving the IP, there is an approximately 76% probability to observe at least 1 participant with an AE if the true incidence of the AE is 2%; if the true incidence rate is 4%, then the probability to observe an AE is approximately 94%.

4.4 Randomization

Approximately 560 participants will be randomized in a 1:1:1:1:1:1:1:1 ratio to receive either mRNA-1020 50 µg, mRNA-1020 100 µg, mRNA-1020 150 µg, mRNA-1030 25 µg, mRNA-1030 50 µg, mRNA-1030 100 µg, mRNA-1010 50 µg, or Flublok 180 µg, with approximately 70 healthy adult participants randomly assigned to each vaccination group. Randomization will be stratified by age (18 to < 50 years versus ≥ 50 to ≤ 75 years) and will be balanced across the 2 age groups within each vaccination group.

4.5 Blinding and Unblinding

This study is an observer-blind study. The investigator, study staff, study participants, site monitors, and Sponsor personnel (or its designees) will be blinded to the IP administered until the study database is locked and unblinded, with certain exceptions; please refer to Section 9.1 of the protocol and Data Blinding Plan (DBP) for details.

5 ANALYSIS POPULATIONS

5.1 Randomization Set

The Randomization Set consists of all participants who are randomly assigned.

DocuSign Envelope ID: 264FC66D-43E3-471B-863F-16B5EDBC3D09

5.2 Full Analysis Set (FAS)

The FAS consists of all randomly assigned participants who receive the IP. Participants will be analyzed according to the group to which they were randomized.

5.3 Per-Protocol (PP) Set

The PP Set consists of all participants in the FAS who comply with the injection schedule, comply with the timings of immunogenicity blood sampling to have a baseline and at least 1 post-injection assessment, do not have influenza infection at baseline through Day 29 (as documented by PCR testing), and have no major protocol deviations that impact the immune response. The PP Set will be used as the primary analysis set for analyses of immunogenicity unless otherwise specified. Participants will be analyzed according to the vaccination group to which they were randomized.

Participants compliant with the timing of immunogenicity blood sampling are required to have a baseline assessment, at least one post-injection assessment, and a Day 29 assessment that is within 21 days to 42 days after injection (-7 / +14 days of Day 29).

Participants with a dosing error will be considered as having a protocol deviation. However, the determination of whether to include/exclude participants from the PP Set due to a dosing error will be based on the dosage difference (in µg) between the actual dose received and the randomized dose. The PP Set exclusion criteria for participants with dosing error are described in Table 2. For the mRNA-1020 and the mRNA-1030 arms, acceptable dosing deviation from the randomized dose is up to +/- 25 µg. For the mRNA-1010 active comparator, participants who are randomized to the active comparator arm but received less than 30 µg (< 30 µg) of the active comparator or any amount of mRNA-1020, mRNA-1030, or Flublok active comparator will be excluded from the PP Set. For the Flublok active comparator, participants who are randomized to the active comparator arm but received less than 120 µg (< 120 µg) of the active comparator or any amount of mRNA-1020, mRNA-1030, or mRNA-1010 active comparator will be excluded from the PP Set.

Table 2 PP Set Exclusion Criteria for Dosing Errors

	Randomized Dose							
	mRNA-1020			mRNA-1030			mRNA-1010 (active comparator) [1]	Flublok (active comparator) [2]
**Actual Dose Received**	50 µg	100 µg	150 µg	25 µg	50 µg	100 µg		
mRNA-1020								
50 µg		Y	Y	Y	Y	Y	Y	Y
100 µg	Y		Y	Y	Y	Y	Y	Y
150 µg	Y	Y		Y	Y	Y	Y	Y
> 0 - 25 µg	Y	Y	Y	Y	Y	Y	Y	Y
> 25 - 50 µg		Y	Y	Y	Y	Y	Y	Y
> 50- 75 µg		Y	Y	Y	Y	Y	Y	Y
> 75 - 100 µg	Y		Y	Y	Y	Y	Y	Y
> 100 - 125 µg	Y		Y	Y	Y	Y	Y	Y
> 125 -150 µg	Y	Y		Y	Y	Y	Y	Y
> 150 – 175 µg	Y	Y		Y	Y	Y	Y	Y
> 175 µg	Y	Y	Y	Y	Y	Y	Y	Y
mRNA-1030								
25 µg	Y	Y	Y		Y	Y	Y	Y
50 µg	Y	Y	Y	Y		Y	Y	Y
100 µg	Y	Y	Y	Y	Y		Y	Y
> 0 - 25 µg	Y	Y	Y		Y	Y	Y	Y
> 25 - 37.5 µg	Y	Y	Y		Y	Y	Y	Y
> 37.5 - 50 µg	Y	Y	Y	Y		Y	Y	Y
> 50- 75 µg	Y	Y	Y	Y		Y	Y	Y
> 75 - 100 µg	Y	Y	Y	Y	Y		Y	Y
> 100 - 125 µg	Y	Y	Y	Y	Y		Y	Y
> 125 µg	Y	Y	Y	Y	Y	Y	Y	Y
mRNA-1010 (active comparator) [1]	Y	Y	Y	Y	Y	Y		Y
Flublok (active comparator) [2]	Y	Y	Y	Y	Y	Y	Y	

Note: Y = Excluded from PP Set.

DocuSign Envelope ID: 264FC66D-43E3-471B-863F-16B5EDBC3D09

5.4 Safety Set

The Safety Set consists of all randomly assigned participants who receive the IP. The Safety Set will be used for all analyses of safety, except for the solicited ARs. Participants will be included in the vaccination group corresponding to what they actually received according to the following rules:

- mRNA-1030 25 µg group: If the received dose of mRNA-1030 is >0 µg and ≤ 37.5 µg
- mRNA-1030 50 µg group: If the received dose of mRNA-1030 is >37.5 µg and ≤ 75 µg
- mRNA-1030 100 µg group: If the received dose of mRNA-1030 is >75 µg
- mRNA-1020 50 µg group: If the received dose of mRNA-1020 is >0 µg and ≤ 75 µg
- mRNA-1020 100 µg group: If the received dose of mRNA-1020 is >75 µg and ≤ 125 µg
- mRNA-1020 150 µg group: If the received dose of mRNA-1020 is > 125 µg
- mRNA-1010 (HA only comparator) group: If the received dose of mRNA-1020/ mRNA-1030/Flublok is 0 µg and mRNA-1010 >0 µg
- Flublok (active comparator) group: If the received dose of mRNA-1020/mRNA-1030/mRNA-1010 is 0 µg and Flublok >0 µg

5.5 Solicited Safety Set

The Solicited Safety Set consists of all participants in the Safety Set who contribute any solicited AR data. The Solicited Safety Set will be used for the analyses of solicited ARs and participants will be included in the vaccination group corresponding to what they actually received as shown in Section 5.4.

DocuSign Envelope ID: 264FC66D-43E3-471B-863F-16B5EDBC3D09

6 STATISTICAL ANALYSIS

6.1 Planned Analyses

6.1.1 Interim Analysis (IA)

An interim analysis will be performed after participants have completed the Day 29 Visit. All data relevant to the IA through the Day 29 Visit will be cleaned (i.e., data that are as clean as possible).

The IA will be performed by a separate team of unblinded statisticians and programmers. Except for a limited number of Sponsor and CRO personnel who will be unblinded to perform the IA, the study site staffs, Investigators, study monitors, and participants will remain blinded until after the final database lock for final analysis.

6.1.2 Internal Safety Team (IST) Review

A blinded IST will comprise Sponsor physicians. The IST will conduct a scheduled review of safety data after approximately 96 participants (approximately 12 per study arm) have completed the Day 8 visit as well as ad hoc safety data review if requested by the study medical monitor and the study team.

6.1.3 Data Safety Monitoring Board (DSMB) Review

The DSMB, composed of external/independent participant matter experts, will conduct unblinded reviews of safety data on an ad hoc basis if any pause rule is met or as otherwise requested by the study team and /or IST. The DSMB will review unblinded IA results and make recommendations to sponsor.

6.1.4 Final Analysis

The final analysis of safety, reactogenicity, and immunogenicity will be performed after all participants have completed all planned study procedures. The results of this analysis will be presented in a final clinical study report, including individual listings.

6.2 General Considerations

The Schedules of Events (SoE) are provided in Table 1 of the protocol Section 1.3.

General considerations for analyses will be applied to this study, unless otherwise specified. All analyses will be performed by treatment arm, unless otherwise specified. Statistical outputs (tables, figures, listings, and datasets) will refer to study participants as participants and will use vaccination, injection of IP and injection interchangeably. All

analyses will be conducted using SAS Version 9.4. All table data will have a corresponding listing.

6.2.1 Data Precision

For the summary statistics of all numerical variables unless otherwise specified, the display precision will follow programming standards. Please see Appendix A for variable display standards.

When count data are presented, the percentage will be suppressed when the count is zero in order to draw attention to the non-zero counts. A row denoted "Missing" will be included in count tabulations where specified on the shells to account for dropouts and missing values. The denominator for all percentages will be the number of participants in that vaccination group within the analysis set of interest, unless otherwise specified.

6.2.2 Statistics for Continuous and Categorical Variables

Continuous variables will be summarized using the following descriptive summary statistics: the number of participants (n), mean, standard deviation (SD), median, minimum (min), and maximum (max).

Categorical variables will be summarized using frequencies and percentages.

6.2.3 Baseline

Baseline results for laboratory measurements, vital signs, immunogenicity, and nasopharyngeal (NP) swab tests will be defined as the last recorded non-missing value prior to study vaccine administration.

6.2.4 Reference Start Date and Study Day

The reference start date is used as the time point from which the study day is calculated. Study days prior to the reference start date will be negative, while study days after the reference start date are positive.

The reference start date is the date of study vaccine administration.

Study day will be calculated as follows:

- Study day prior to vaccination will be calculated as:

  date of assessment/event – the reference start date

DocuSign Envelope ID: 264FC66D-43E3-471B-863F-16B5EDBC3D09

- Study day on or after the date of vaccination will be calculated as:

  date of assessment/event – the reference start date + 1.

An assessment/event date on the reference start date will be study day 1.

6.2.5 Handling Non-Quantifiable Antibody Values

For calculation regarding antibody levels/titers, antibody values reported as below lower limit of quantification (LLOQ) will be replaced by 0.5 × LLOQ. Values that are greater than the upper limit of quantification (ULOQ) will be converted to the ULOQ. Missing results will not be imputed.

6.2.6 Analysis Periods for Safety Analyses

The following analysis periods for safety analyses will be used in this study:

- 7 days following vaccination: this period includes the day of vaccination and 6 subsequent days. This analysis period will be used for solicited local and systemic ARs that occur during this time.
- 28 days following vaccination: this period includes the day of vaccination and 27 subsequent days. This analysis period will be used for unsolicited AEs.
- Overall period: this analysis period starts on Day 1 and continues through the earliest of the following: Day 181/study completion, discontinuation from the study, or death.

6.2.7 Unscheduled Visits and Visit Windowing Rules

Unscheduled visit measurements will be included in analysis as follows:

- In scheduled visit windows per specified visit windowing rules.
- In the derivation of baseline/last on-treatment values.
- In the derivation of max/min on-treatment values and max/min change from baseline values for safety analyses.
- In individual participant data listings as appropriate.

The analysis visit windows for protocol-defined visits are provided in Appendix B.

6.2.8 Handling Missing and Incomplete Data

- Imputation rules for missing dates of prior/concomitant medications, non-study vaccinations and procedures are provided in Appendix C.
- Imputation rules for missing AE dates are provided in Appendix D.
- For laboratory assessments, if majority of results are indefinite, imputation of these values will be considered. If the laboratory results are reported as below the LLOQ (e.g., < 0.1), the numeric values will be replaced by 0.5 × LLOQ in the summary. If the laboratory results are reported as greater than the ULOQ (e.g., > 3000), the numeric values will be replaced by ULOQ in the summary.
- Other incomplete/missing data will not be imputed, unless otherwise specified.

6.2.9 Age and Vaccination Groups

Age groups:

The following age groups will be used for summary purposes:

- Overall
- Age: ≥ 18 to < 50 years
- Age: ≥ 50 to ≤ 75 years

Vaccination groups:

The following vaccination groups will be used for summary purposes:

- mRNA-1030 25 µg
- mRNA-1020 50 µg
- mRNA-1030 50 µg
- mRNA-1020 100 µg
- mRNA-1030 100 µg
- mRNA-1020 150 µg
- mRNA-1010 50 µg
- Flublok 180 µg

All analyses and data summaries/displays will be provided by vaccination group (and then by age group) using appropriate analysis population, unless otherwise specified.

6.2.10 Adjustment for Covariates

The estimates of GMFR with 95% CIs will be calculated based on analysis of covariance (ANCOVA) model adjusted for baseline titer, age group and /or other covariates. Antibody titers are analyzed using $\log_2$ titers or $\log_2$ fold rise and back transformed to estimate GMFRs with 95% CIs. After transformation, the data tend to be normally distributed.

6.3 Background Characteristics

The following description of background characteristics analyses will be applied to this study, unless otherwise specified.

6.3.1 Participant Disposition

The number and percentage of participants in the following categories will be summarized by randomized vaccination group and then further by age group as defined in Section 6.2.9 based on Randomization Set:

- Randomization Set
- Full Analysis Set (FAS)
- Per-protocol (PP) Set
- Safety Set
- Solicited Safety Set

The percentage will be based on participants in that vaccination group within the Randomization Set. A summary of reasons for participants who are in the Randomization Set but excluded from PP Set will also be provided. Listing of analysis sets will be provided based on the Randomization Set.

The number of participants in the following categories will be summarized based on participants screened:

- Number of participants screened
- Number and percentage of screen failure participants and the reason for screen failure

The percentage of participants who screen failed will be based on the number of participants screened. The percentage of participants for each reason for screen failure will be based on the number of participants who screen failed. For screened failure participants, age (years), as well as sex, race, ethnicity, and reasons for screen failure will be presented in a listing.

The number and percentage of participants in each of the following disposition categories will be summarized by vaccination group (and then by age group) based on the Randomization Set:

- Received injection
- Completed study
- Discontinued from the study and the reason for discontinuation

A participant disposition listing will be provided, including informed consent date, injection date, date of study completion, date of study discontinuation, with reasons for discontinuation.

A participant who completed Day 181/EoS (Month 6 visit), defined as 6 months after the study vaccination on Day 1, is considered to have completed the study.

6.3.2 Demographics

Descriptive statistics will be calculated for the following continuous demographic and baseline characteristics: age (years), weight (kg), height (cm), and body mass index (BMI) (kg/m^2). Number and percentage of participants will be provided for categorical variables such as gender, race, and ethnicity. The summaries will be presented by vaccination group and overall based on the Randomization Set, FAS, PP Set, and Safety Set. If the Safety Set differs from the Randomization Set (e.g., participants randomized but not received any study vaccination or participants received study vaccination other than the vaccination group they were randomized to), the analysis will also be conducted using the Randomization Set. Summary of RT-PCR SARS-CoV-2 results at baseline, baseline RT-PCR Influenza Results and Seasonal Influenza Vaccine since Sep 2021.

Listing of demographics and baseline characteristics will be provided based on the Randomization Set.

6.3.3 Medical History

Medical history data will be coded by system organ class (SOC) and preferred term (PT) using the Medical Dictionary for Regulatory Activities (MedDRA).

The number and percentage of participants with any medical history will be summarized by SOC and PT based on the Safety Set. A participant will be counted only once for multiple events within each SOC and PT. SOCs will be displayed in internationally agreed order. PTs will be displayed in descending order of frequency in total of each type of vaccination group/overall group and then alphabetically within SOC.

Medical history data will be presented in a listing.

6.3.4 Prior and Concomitant Medications

Prior and concomitant medications and non-study vaccination will be coded using the World Health Organization drug dictionary (WHODrug Global). The summary of concomitant medications will be based on the Safety Set. Categorization of prior, concomitant, and post medications is summarized in Appendix C.

The number and percentage of participants using concomitant medications and non-study vaccination during the 7-day follow-up period (i.e., on the day of injection and the 6 subsequent days) and during the 28-day follow-up period after the injection (i.e., on the day of injection and the 27 subsequent days) will be summarized by vaccination groups as follows:

- Any concomitant medications and non-study vaccination within 7 days post-injection
- Any concomitant medications within 28 days post-injection
- Any non-study vaccination within the period starting 28 Days before IP Injection and through Day 181/EoS
- Any Seasonal influenza vaccine from September 2021 through Day 181/EoS
- Any Covid-19 Vaccine before the IP Injection and through Day 181/EoS
- Antipyretic or analgesic medication within 28 days post-injection

A summary table of concomitant medications and non-study vaccination that continued or was newly received on the date of injection through 28 days post-injection will be provided by PT in descending frequency in the total group.

Medications taken to prevent or treat pain or fever within 7 days after injection will be collected in the electronic diary (eDiary), and summaries will be provided based on the Solicited Safety Set by vaccination group (and then by age group) as defined in Section 6.2.9.

Prior, concomitant, post medications, and non-study vaccination will be presented in a listing. Medications taken to prevent or treat pain or fever will also be presented in a listing.

Concomitant procedures will be presented in a listing.

6.3.5 Study Exposure

Study IP administration data will be presented in a listing. Participants with dosing error will be presented in a listing.

Study duration will be summarized from randomization and from the injection based on the Safety Set.

6.3.6 Major Protocol Deviations

Major protocol deviations are a subset of protocol deviations that may significantly impact the completeness, accuracy, or reliability of the study data or that may significantly affect a participant’s rights, safety, or well-being. Major protocol deviations rules will be developed and finalized before database lock.

The number and percentage of the participants with each major protocol deviation type will be provided by vaccination group (and then by age group) based on the Randomization Set.

Major protocol deviations will be presented in a listing.

If the protocol deviations (PDs) are classified as Important/Non-Important’, then Important means Major and Non-Important means Minor.

6.3.7 COVID-19 Impact

A listing will be provided for the impact of coronavirus disease 2019 (COVID-19) on the execution of the study.

DocuSign Envelope ID: 264FC66D-43E3-471B-863F-16B5EDBC3D09

6.4 Safety Analysis

All safety analyses will be based on the Safety Set, except summaries of solicited ARs, which will be based on the Solicited Safety Set. All safety analyses will be provided by vaccination group and by age group. Participants will be included in the vaccination group corresponding to what they actually received.

Safety and reactogenicity will be assessed by clinical review of all relevant parameters including the following:

- solicited local and systemic ARs
- unsolicited AEs (including any clinical safety laboratory abnormalities)
- treatment-related AEs
- severe AEs
- SAEs
- AESIs
- MAAEs
- AEs leading to withdrawal from study participation
- vital sign measurements
- physical examination findings

Unsolicited AEs will be coded by system organ class (SOC) and preferred term (PT) according to MedDRA. Solicited ARs will be coded according to the MedDRA for AR terminology.

All table data will have a corresponding listing. Safety phone calls data will be provided in a listing. Deaths will be provided in a listing.

6.4.1 Unsolicited Adverse Events

An unsolicited AE is any AE reported by the participant that is not specified as a solicited AR in the protocol or is specified as a solicited AR but starts outside the protocol-defined period for reporting solicited ARs (i.e., for the 7 days after the injection of IP).

Only treatment-emergent AE (TEAE) will be included in the analysis. A TEAE is defined as any event occurring during the study not before exposure to study vaccine or any event already present that worsens in intensity or frequency after exposure to study vaccine.

DocuSign Envelope ID: 264FC66D-43E3-471B-863F-16B5EDBC3D09

AEs will also be evaluated by the investigator for MAAE and AESI. An MAAE is defined as an AE that leads to an unscheduled visit to a healthcare practitioner (HCP). An AESI is an AE (serious or nonserious) of scientific and medical concern specific to the Sponsor's product or program, for which ongoing monitoring and immediate notification by the Investigator to the Sponsor are required. The AESIs for this study are listed in APPENDIX 3 of the protocol Section 11.3.

Unsolicited AEs within 28 days after vaccination will be summarized by vaccination group. Unsolicited SAE, unsolicited AESI, unsolicited MAAE, and unsolicited AE leading to withdrawal from the study up to 28 days after vaccination and overall period will be summarized by vaccination group. See Section 6.2.9 and Section 6.2.6 for definitions of analysis groups and analysis period respectively.

All summary tables (except for the overall summary of AEs) for unsolicited TEAEs will be presented by SOC and/or PT, and by SOC and PT and severity, with counts of participants included. SOCs will be displayed in internationally agreed order. PTs will be displayed in descending order of frequency of total in each vaccine type group and then alphabetically within SOC. When summarizing the number and percentage of participants with an event, participants with multiple occurrences of the same AE or a continuing AE will be counted once. Participants will be presented according to the highest severity in the summaries by severity, if participants reported multiple events under the same SOC and/or PT.

Percentages will be based upon the number of participants in the Safety Set within each vaccination group.

6.4.1.1 Incidence of Unsolicited Adverse Events

An overall summary of unsolicited TEAEs including the number and percentage of participants who experience the following within 28 days after vaccination (See Section 6.2.6 for safety analysis periods) will be presented:

- Any unsolicited TEAEs (See Section 6.2.6 for study periods)
- Any unsolicited serious TEAEs
- Any unsolicited fatal TEAEs
- Any unsolicited medically attended TEAEs
- Any unsolicited treatment-emergent AESI

DocuSign Envelope ID: 264FC66D-43E3-471B-863F-16B5EDBC3D09

- Any unsolicited TEAEs leading to discontinuation from participation in the study
- Any unsolicited severe TEAEs

The table will also include number and percentage of participants with unsolicited TEAEs within 28 days after vaccination that are treatment-related in each of the above categories.

In addition, separate listings containing individual participant AE data for unsolicited TEAEs, unsolicited TEAEs leading to withdrawal from study participation, unsolicited AESI, and unsolicited medically attended TEAEs will be provided. Unsolicited treatment-related TEAEs, unsolicited severe TEAEs, unsolicited treatment-related severe TEAEs, unsolicited serious treatment-related AEs, unsolicited treatment-related medically attended TEAEs, unsolicited adverse events leading to death, and unsolicited TEAEs for participants with influenza or SARS-CoV-2 infection will also be provided in corresponding listings.

6.4.1.2 TEAEs by System Organ Class and Preferred Term

The following summary tables of TEAEs will be provided by SOC and PT using frequency counts and percentages (i.e., number and percentage of participants with an event):

- All unsolicited TEAEs
- All unsolicited TEAEs that are treatment-related
- All unsolicited TEAEs leading to withdrawal from study participation
- All unsolicited severe TEAEs
- All unsolicited severe TEAEs that are treatment-related
- All unsolicited treatment-emergent AESIs
- All unsolicited medically attended TEAEs
- All unsolicited medically attended TEAEs that are treatment-related

6.4.1.3 TEAEs by Preferred Term

Summary tables of all unsolicited TEAEs within 28 days after vaccination will be provided by preferred term. PTs will be sorted in a descending order according to the frequency in total in each type of vaccination group.

DocuSign Envelope ID: 264FC66D-43E3-471B-863F-16B5EDBC3D09

6.4.1.4 TEAEs by System Organ Class, Preferred Term, and Severity

The following summary tables of TEAEs within 28 days after vaccination will be provided by SOC, PT, and maximum severity (mild, moderate, severe) using frequency counts and percentages:

- All unsolicited TEAEs
- All unsolicited TEAEs that are treatment-related

6.4.2 Serious Adverse Events

An AE (including an AR) is considered an SAE if, in the view of either the Investigator or Sponsor, it results in any of the following outcomes:

- Death
- Life-threatening
- Inpatient hospitalization or prolongation of existing hospitalization
- Persistent or significant incapacity or substantial disruption of the ability to conduct normal life functions
- Congenital anomaly or birth defect
- Medically important event

The overall summary of unsolicited TEAEs in Section 6.4.1.1 will include the following:

- Any unsolicited serious TEAEs
- Any unsolicited serious treatment-related TEAEs

The following summary tables of unsolicited serious TEAEs within 28 days and overall period will be provided by SOC and PT using frequency counts and percentages (i.e., number and percentage of participants with an event):

- All unsolicited serious TEAEs
- All unsolicited serious TEAEs that are treatment-related

6.4.3 Solicited Adverse Reactions

Solicited ARs refers to selected signs and symptoms occurring after vaccination during a specified post-vaccination follow-up period (day of vaccination and 6 subsequent days). The solicited ARs are recorded daily by the participant in the eDiary.

DocuSign Envelope ID: 264FC66D-43E3-471B-863F-16B5EDBC3D09

If a participant reported a solicited AR during the solicited period and did not record the event in the eDiary, the event should be recorded on the Reactogenicity page of the eCRF. If the event starts during the solicited period, but continues beyond 7 days after dosing, the participant should notify the site to provide an end date to close out the event on the Reactogenicity page of the eCRF. If the participant reported an event after the solicited period (i.e., after Day 7). it should be recorder as an AE on the AE page of the eCRF.

All solicited ARs (local and systemic) will be considered causally related to dosing or study vaccination.

Analyses of solicited ARs will be provided by vaccination group based on the Solicited Safety Set. All solicited ARs (overall, local, and systemic) reported during the 7-day follow-up period after vaccination will be summarized. A 2-sided 95% CI using the Clopper-Pearson method will also be provided for the percentage of participants with any solicited AR.

- The number and percentage of participants who reported solicited local ARs and solicited systemic ARs during the 7-day follow-up period after injection or persisting beyond 7 days after injection will be tabulated by vaccination group and toxicity grade.
- The number and percentage of participants who reported each individual solicited AR will also be summarized by vaccination group, toxicity grade, and day of reporting based on the eDiary. Analyses will be displayed for each age group.

A two-sided 95% CI using the Clopper-Pearson method will be provided for the percentage of participants who reported any solicited AR, any solicited local AR, or any solicited systemic AR.

The onset of individual solicited ARs is defined as the time point after injection at which the respective solicited AR first occurred. The number and percentage of participants with onset of individual solicited AR within 7 days will be summarized by vaccination group and onset day relative to injection day (Day 1). Analyses will be displayed for each age group.

The number of days reporting each solicited AR will be summarized descriptively and categorically for the following time windows (1-2 days, 3-4 days, 5-6 days, and 7 days) by vaccination group (and then by age group).

The number of days will be calculated as the day(s) the solicited AR is reported within 7 days of injection including the day of injection, no matter if it is intermittent or continued.

Solicited local, solicited systemic ARs and solicited ARs persisting beyond 7 days collected in the eDiary will be provided in corresponding listings, and the maximum toxicity grade from eDiary will be presented. Severity grading of reactogenicity will occur automatically based on participant entry into the eDiary according to the grading scales presented in Table 7 of the protocol Section 8.4.3. modified from the Toxicity Grading Scale for Healthy Adult and Adolescent Volunteers Enrolled in Preventive Vaccine Clinical Trials (DHHS 2007b).

6.4.4 Clinical Laboratory Evaluations

Safety laboratory testing will include hematology and serum chemistry.

For continuous hematology and serum chemistry parameters, the observed values and changes from baseline will be summarized at each time point by vaccination group and then by age group as defined in Section 6.2.9.

Summary of Grade 3+ laboratory abnormalities by visit and toxicity grades will be provided by vaccination group and then by age group as defined in Section 6.2.9.

Hematology and chemistry toxicity grades will be summarized using a shift table from baseline to Day 8 by vaccination group.

All laboratory test results will be presented in the data listings. The results that are outside the reference ranges will be flagged in the data listings. The abnormalities meeting the toxicity grading criteria (Grade 3 or higher) in Appendix E in any safety laboratory (hematology and serum chemistry) will be listed separately.

A pregnancy test will be performed on all female participants of childbearing potential at the Screening Visit and before vaccine administration on Day 1 via point-of-care urine, and as needed at unscheduled visits (urine or blood pregnancy test based on the investigator’s discretion).

The pregnancy test results will be listed.

6.4.5 Vital Sign Measurements

Vital sign measurements, including systolic and diastolic blood pressures (BP), heart rate, respiratory rate, and body temperature, will be presented in a data listing. The values that are outside the reference ranges will be flagged in the data listing. The abnormalities

meeting the toxicity grading criteria (Grade 3 or higher) in Appendix F in any vital sign measurement will be listed separately.

Observed values and changes from pre-vaccination (baseline) to post-vaccination at Day 1 for all vital sign measurements will be summarized by vaccination group and then by age group as defined in Section 6.2.9.

Shift from baseline in the toxicity grades to post-vaccination result at Day 1 will also be summarized by vaccination group and then by age group.

6.4.6 Physical Examinations

A full physical examination, including height and weight, will be performed at scheduled time points as indicated in the Table 1 of the protocol Section 1.3.

The full examination will include assessment of skin, head, ears, eyes, nose, throat, neck, thyroid, lungs, heart, cardiovascular system, abdomen, lymph nodes, and musculoskeletal system and extremities.

Any clinically significant finding identified during a study visit before administration of study vaccine will be recorded in the participant's medical history eCRF.

Any clinically significant finding identified during a study visit after administration of study vaccine will be reported as an MAAE.

Symptom-directed physical examinations may be performed at other time points at the discretion of the Investigator.

Summary tables or listing will not be provided for physical examinations.

6.4.7 Electrocardiogram (ECG)

12-lead ECG measurements will be recorded after 10 minutes of supine rest at Visit 1/Day 1 prior to vaccination. Summary table or listing will not be provided for ECG.

6.5 Immunogenicity Analysis

The analyses of immunogenicity will be based on the PP Set and will be performed by vaccination group and by age group.

If the number of participants in the FAS and PP Set differs (defined as the difference divided by the total number of participants in the PP Set) by more than 10% for each age

group, supportive analyses of immunogenicity may be conducted using the FAS. The supportive analysis is required if the condition is met.

The GMT and geometric mean (GM) level will be calculated using the following formula:

$$2^{\left\{\frac{\sum_{i=1}^{n} log_2(t_i)}{n}\right\}}$$

where $t_1, t_2, \ldots, t_n$ are n observed immunogenicity titers or levels.

The GMFR measures the changes in immunogenicity titers or levels within participants. The GMFR will be calculated using the following formula:

$$2^{\left\{\frac{\sum_{i=1}^{n} log_2\left(^{v_{ij}}/_{v_{ik}}\right)}{n}\right\}} = 2^{\left\{\frac{\sum_{i=1}^{n} log_2(v_{ij})-log_2(v_{ik})}{n}\right\}}$$

where, for *n* participants, v_{ij} and v_{ik} are observed immunogenicity titers or levels for participant *i* at time points *j* and *k*, $j \neq k$.

The 95% CIs for GMT and GMFR will be calculated based on the t distribution of the log-transformed values then back transformed to the original scale for presentation, unless otherwise specified. Reverse cumulative distribution plot will be generated.

The model-based GM titer will be estimated based on an analysis of covariance (ANCOVA) model. In the ANCOVA model, the log-transformed antibody titer at a post baseline timepoint (Day 29) are treated as a dependent variable, with the treatment group as an explanatory variable and the log-transformed baseline antibody titer as a covariate, adjusting for the stratification factor(s) as appropriate, that is, age groups (18 to 49 years old, and 50 to 75 years old of age).

The GMT will be estimated by the geometric least square mean (GLSM) from the ANCOVA model for each treatment group and corresponding 95% CI will be provided.

For each pair of between-group comparison specified in Table 3, the GMR (ratio of GMTs) between the two treatment groups in each pair will be estimated from the ANCOVA model, with 95% CI provided accordingly.

For each pair of between-group comparison specified in Table 3, the difference of seroconversion rate (SCR) between the two treatment groups in each pair at Day 29 will be provided, with 95% CI estimated using Miettinen-Nurminen method.

DocuSign Envelope ID: 264FC66D-43E3-471B-863F-16B5EDBC3D09

Table 3 Exploratory Between-Group Immunogenicity Comparisons

Treatment Group	Group #1	Group #2	Group #3	Group #4	Group #5	Group #6	Group #7	Group #8
**Group #1**	NA	NA	NA	NA	NA	NA	NA	NA
**Group #2**	NA	NA	NA	NA	NA	NA	NA	NA
**Group #3**	#3 vs. #1	#3 vs. #2	NA	NA	NA	NA	NA	NA
**Group #4**	#4 vs. #1	#4 vs. #2	#4 vs. #3	NA	NA	NA	NA	NA
**Group #5**	#5 vs. #1	#5 vs. #2	#5 vs. #3	#5 vs. #4	NA	NA	NA	NA
**Group #6**	#6 vs. #1	#6 vs. #2	#6 vs. #3	#6 vs. #4	#6 vs. #5	NA	NA	NA
**Group #7**	#7 vs. #1	#7 vs. #2	#7 vs. #3	#7 vs. #4	#7 vs. #5	#7 vs. #6	NA	NA
**Group #8**	#8 vs. #1	#8 vs. #2	#8 vs. #3	#8 vs. #4	#8 vs. #5	#8 vs. #6	#8 vs. #7	NA

Note: The 8 treatment groups can be found in Table 1 and the order might be re-arranged for easier reference.

6.5.1 Immunogenicity Assessments

The following immunogenicity assessments are planned:

- Serum antibody level as measured by HAI assay for primary, secondary, and exploratory analyses

- NA-specific antibody levels as measured by NAI assay for primary, secondary, and exploratory analyses

- Serum neutralizing antibody level as measured by microneutralization (MN) assay or similar method for secondary or exploratory analysis

DocuSign Envelope ID: 264FC66D-43E3-471B-863F-16B5EDBC3D09

- Cellular immunogenicity for exploratory subset analysis. No summary table or listing will be provided.

6.5.2 Primary Analysis of Immunogenicity

GMTs and GMFRs:

For each vaccination group, the following evaluations will be performed at Day 1 (if applicable) and Day 29.

- GMT of vaccine-matched strain-specific anti-HA antibodies with corresponding 95% CI will be provided at Day 1 and Day 29, GMT of vaccine-matched strain-specific anti-NA antibodies with corresponding 95% CI will be provided at Day 1 and Day 29, GMFR of vaccine-matched strain-specific anti-HA antibodies with corresponding 95% CI will be provided at Day 29 over pre-injection baseline at Day 1.
- GMFR of vaccine-matched strain-specific anti-NA antibodies with corresponding 95% CI will be provided at Day 29 over pre-injection baseline at Day 1.
- In addition, the following descriptive statistics will also be provided at Day 29: the number of participants (n), median, minimum, and maximum.

Seroconversion:

For each vaccination group, the following evaluations will be performed at Day 29.

- For HA, the number and percentage of participants with seroconversion at Day 29 will be provided with 2-sided 95% CI using the Clopper-Pearson method.

For HA, seroconversion is defined as:

1. If LLOQ is 1:10, a Day 29 titer ≥ 1:40 if baseline is < 1:10 or a 4-fold or greater rise if baseline is ≥ 1:10 in anti- HA antibodies measured by HAI assay.
2. If LLOQ is greater than 1:10 (e.g. 1:14), a Day 29 titer ≥ 4 times of LLOQ if baseline is < LLOQ or a 4-fold or greater rise if baseline is ≥ LLOQ in anti- HA antibodies measured by HAI assay.

2-/3-/4-fold Rise:

For NA, an endpoint of interest is the percentage of participants with a change in the

Day 29 titer of at least 2-/3-/4-fold rise from baseline.

Definition: 2-/3-/4-fold rise is defined as ≥ 2-/3-/4-fold of the LLOQ if the Day 1 titer is < LLOQ; or ≥ 2-/3-/4-fold of the Day 1 titer if the Day 1 titer ≥ LLOQ.

For each vaccination group, the following evaluations will be performed at Day 29.

- The number and percentage of participants with a ≥ 2-fold rise at Day 29 in anti-NA antibody titers from baseline will be provided with 2-sided 95% CI using the Clopper-Pearson method.
- The number and percentage of participants with a ≥ 3-fold rise at Day 29 in anti-NA antibody titers from baseline will be provided with 2-sided 95% CI using the Clopper-Pearson method.
- The number and percentage of participants with a ≥ 4-fold rise at Day 29 in anti-NA antibody titers from baseline will be provided with 2-sided 95% CI using the Clopper-Pearson method.

6.5.3 Secondary Analysis of Immunogenicity

For each vaccination group, the following evaluations will be performed at all evaluable humoral immunogenicity time points.

GMTs and GMFRs:

- GMT of vaccine-matched strain-specific anti-HA antibodies with corresponding 95% CI will be provided at each time point,. Geometric mean (GM) level and corresponding 95% CI will be plotted at each time point.
- GMT of vaccine-matched strain-specific anti-NA antibodies with corresponding 95% CI will be provided at each time point.GM level and corresponding 95% CI will be plotted at each time point.
- GMFR of vaccine-matched strain-specific anti-HA antibodies with corresponding 95% CI will be provided at each post-baseline time point over pre-injection baseline at Day 1. GMFR and corresponding 95% CI will be plotted at each time point.
- GMFR of vaccine-matched strain-specific anti-NA antibodies with corresponding 95% CI will be provided at each post-baseline time point over pre-injection

DocuSign Envelope ID: 264FC66D-43E3-471B-863F-16B5EDBC3D09

baseline at Day 1. GMFR and corresponding 95% CI will be plotted at each time point.

- In addition, the following descriptive statistics will also be provided at each time point: the number of participants (n), median, minimum, and maximum.
- Reverse cumulative distribution function curve at each time point will be provided.
- Listing of anti-HA antibodies for vaccine-matched seasonal influenza A and B strains will be provided based on the PP Set.
- Listing of anti-HA antibodies for vaccine-matched seasonal influenza A and B strains for participants with influenza infection will be provided based on the PP Set.
- Listing of anti-NA antibodies for vaccine-matched seasonal influenza A and B strains will be provided based on the PP Set.
- Listing of anti-NA antibodies for vaccine-matched seasonal influenza A and B strains for participants with influenza infection will be provided based on the PP Set.

Seroconversion:

For each vaccination group, the following evaluations will be performed at each post-baseline time point.

- For HA, the number and percentage of participants with seroconversion at each post-baseline time point will be provided with 2-sided 95% CI using the Clopper-Pearson method.

2-/3-/4-fold Rise:

For NA: For each vaccination group, the following evaluations will be performed at each post-baseline time point.

- The number and percentage of participants with a ≥ 2-fold rise at each post-baseline time point in anti-NA antibody titers from baseline will be provided with 2-sided 95% CI using the Clopper-Pearson method.

DocuSign Envelope ID: 264FC66D-43E3-471B-863F-16B5EDBC3D09

- The number and percentage of participants with a ≥ 3-fold rise at each post-baseline time point in anti-NA antibody titers from baseline will be provided with 2-sided 95% CI using the Clopper-Pearson method.
- The number and percentage of participants with a ≥ 4-fold rise at each post-baseline time point in anti-NA antibody titers from baseline will be provided with 2-sided 95% CI using the Clopper-Pearson method.

6.5.4 Exploratory Analysis of Immunogenicity

The below exploratory analyses of immunogenicity may be performed:

- GMT of vaccine-mismatched strain-specific anti-HA antibodies with corresponding 95% CI will be provided at each time point using methods described in Section 6.5.
- GMT of vaccine-mismatched strain-specific anti-NA antibodies with corresponding 95% CI will be provided at each time point using methods described in Section 6.5.
- GMFR of vaccine-mismatched strain-specific anti-HA antibodies with corresponding 95% CI will be provided at each post-baseline time point over pre-injection baseline at Day 1 using methods described in Section 6.5.
- GMFR of vaccine-mismatched strain-specific anti-NA antibodies with corresponding 95% CI will be provided at each post-baseline time point over pre-injection baseline at Day 1 using methods described in Section 6.5.
- Summary of RT-PCR results at Baseline.
- Summary of detected influenza results by RT-PCR at post-baseline by time period.
- Listing of anti-HA antibodies for vaccine-mismatched influenza A and B strains will be provided based on the PP Set.
- Listing of anti-HA antibodies for vaccine-mismatched influenza A and B strains for participants with influenza infection will be provided based on the PP Set. Listing of anti-NA antibodies for vaccine-mismatched influenza A and B strains will be provided based on the PP Set.
- Listing of anti-NA antibodies for vaccine-mismatched influenza A and B strains for participants with influenza infection will be provided based on the PP Set.

DocuSign Envelope ID: 264FC66D-43E3-471B-863F-16B5EDBC3D09

6.6 Analysis of Influenza Infection

Influenza infection during the study is an exploratory endpoint, and the analyses of influenza infection confirmed by RT-PCR test may be performed using the FAS.

Due to the ongoing COVID-19 pandemic, participants may get infected with SARS-CoV-2 or experience symptoms consistent with influenza-like illness (ILI) during the study.

NP swab samples will be collected from all participants before the injection on Day 1 for assessment of asymptomatic infection with respiratory pathogens, including influenza virus and SARS-CoV-2, as influenza or COVID-19 symptoms may confound reactogenicity assessment. Furthermore, if a participant experiences any signs or symptoms suggesting ILI and/or COVID-19, an additional NP swab sample will be collected to confirm the diagnosis via RT-PCR.

A CDC-defined ILI is defined as body temperature ≥ 37.8°C (100°F) accompanied by cough and/or sore throat. An RT-PCR confirmed ILI is defined as a positive influenza result by RT-PCR done at any setting during the study period.

Summaries below will be provided, unless otherwise specified.

- RT-PCR test results at baseline will be summarized by test and vaccination group. Participants with positive influenza results at baseline will be presented in a listing.
- The number and percentage of participants with at least one positive influenza result at post-baseline will be provided by vaccination group. Participants with at least one positive influenza result at post-baseline will be presented in a listing.

6.7 Interim Analysis

One interim analysis (IA) is planned in this study. It will be performed after participants have completed the Day 29 visit.

This interim analysis will be focused on providing insight on the primary study objectives using the available data.

The IA will include safety and immunogenicity and will be performed after participants have completed the Day 29 visit. All data relevant to the IA through the Day 29 visit will be cleaned (i.e., data that are as clean as possible).

A limited number of Sponsor and CRO personnel will be unblinded to perform the IAs. Please refer to the DBP for more details on the blinding of IA.

An independent, unblinded statistics team will carry out the IA. The unblinded statistics team will not be involved in either study design or the regular study conduct. The study site staffs, investigators, study monitors, and participants will remain blinded until after the final database lock for final analysis.

6.8 Internal Safety Team Review

The safety monitoring for this study will include a group of blinded study team members, inclusive of, at a minimum, a Sponsor medical monitor, a CRO medical monitor, a blinded IST, and an unblinded DSMB.

The IST will comprise Sponsor physicians. The IST will conduct a scheduled review of safety data after approximately 96 participants (12 per study arm) have completed the Day 8 visit as well as ad hoc safety data reviews if requested by the study team. Enrollment will continue while these reviews are conducted if no pause rules have been met and the study team has not identified any safety concerns.

The IST will also conduct ad hoc reviews as requested by the study medical monitor and the study team. Details regarding the IST composition, responsibilities, procedures, and frequency of data review will be defined in the mRNA-1020-P101 Internal Safety Team (IST) Charter.

6.9 Data Safety Monitoring Board Review

An independent unblinded DSMB will be used throughout the conduct of this study. This committee will be composed of independent members with relevant therapeutic and/or biostatistical expertise to allow for the ongoing unblinded review of safety data from this study population.

The DSMB will conduct unblinded reviews of safety data on an ad hoc basis if any pause rule is met or as requested by the study team and /or IST.

The DSMB will also review unblinded IA results provided by the independent unblinded statistician.

A secure file transfer path will be decided between the CRO and Sponsor to use for the transfer of unblinded study data for DSMB review. Only the CRO independent unblinded

DocuSign Envelope ID: 264FC66D-43E3-471B-863F-16B5EDBC3D09

biostatistician, unblinded programmer(s), unblinded medical monitor (if assigned for unblinded DSMB review), and unblinded Sponsor clinical representative (if assigned for unblinded DSMB review) will have access to upload the unblinded data package to the secured file transfer location to be delivered to the DSMB. Refer to the mRNA-1020-P101 Data Safety Monitoring Board (DSMB) Charter for more details.

6.10 Subgroup Analysis

The protocol does not define any formal subgroup analyses, and the study is not adequately powered to perform formal subgroup analyses. However, age-specific subgroup analyses may be performed to explore for potential differences in safety/reactogenicity or immune responses across age groups.

7 CHANGES FROM PLANNED ANALYSES IN PROTOCOL

Not applicable.

8 REFERENCES

Department of Health and Human Services (DHHS), Food and Drug Administration (FDA), Center for Biologics Evaluation and Research (US). Guidance for industry: Toxicity grading scale for healthy adult and adolescent volunteers enrolled in preventative vaccine clinical trials. September 2007.

Available from:
https://www.fda.gov/downloads/BiologicsBloodVaccines/GuidanceComplianceRegulatoryInformation/Guidances/Vaccines/ucm091977.pdf.

9 LIST OF APPENDICES

9.1 *Appendix A Standards for Safety and Immunogenicity Variable Display in TLFs*

Continuous Variables: The precision for continuous variables will be based on the precision of the data itself. The mean and median will be presented to one decimal place more than the original results; the SD will be presented to two decimal places more than the original results; the minimum and maximum will be presented to the same precision as the original results.

Categorical Variables: Percentages will be presented to one decimal place.

9.2 *Appendix B Analysis Visit Windows for Safety and Immunogenicity Analysis*

Safety and Immunogenicity Analysis will be summarized using the following analysis visit window for post injection assessments:

Step 1: If the safety and immunogenicity assessments are collected at scheduled visit, i.e., nominal scheduled visit, the data collected at scheduled visit will be used.

Step 2: If the safety and immunogenicity assessments are not collected at the scheduled visit, assessments collected at unscheduled visit will be used using the analysis visit windows described in Table 4 below.

If a participant has multiple assessments within the same analysis visit, the following rule will be used:

- If multiple assessments occur within a given analysis visit, the assessment closest to the target study day will be used.
- If there are 2 or more assessments equal distance to the target study day, the last assessment will be used.

Table 4 Analysis Visit Window

Visit	Target Study Day	Visit Window in Study Day
**Labs**		
Day 8	8	[2, 18]
**Immunogenicity**		
Day 1	1 (Date of Vaccination)	1, Pre-vaccination
Day 8	8	[2, 18]
Day 29	29	[19, 105]
Day 181	181	≥106

9.3 *Appendix C Imputation Rules for Missing Prior/Concomitant Medications and Non-Study Vaccinations Dates*

Imputation rules for missing or partial medication start/end dates are defined below:

1. Missing or partial medication start date:
   - If only Day is missing, use the first day of the month, unless:
     - The medication end date is after the date of injection or is missing AND the start month and year of the medication coincide with the start month and year of the injection. In this case, use the date of injection.
   - If Day and Month are both missing, use the first day of the year, unless:
     - The medication end date is after the date of injection or is missing AND the start year of the medication coincide with the start year of the injection. In this case, use the date of injection
   - If Day, Month, and Year are all missing, the date will not be imputed, but the medication will be treated as though it began prior to the injection for purposes of determining if status as prior or concomitant.
2. Missing or partial medication end date:
   - If only Day is missing, use the earliest date of (last day of the month, study completion, discontinuation from the study, or death).
   - If Day and Month are both missing, use the earliest date of (last day of the year, study completion, discontinuation from the study, or death).
   - If Day, Month, and Year are all missing, the date will not be imputed, but the medication will be flagged as a continuing medication.

In summary, the prior, concomitant, or post categorization of a medication is described in Table 5 below.

Table 5 Prior, Concomitant, and Post Categorization of Medications and Non-Study Vaccinations

	Medication End Date		
**Medication Start Date**	**< Injection Date of IP**	**≥ Injection Date and ≤ Injection Date + 27 Days**	**> Injection Date + 27 days [1]**
< Injection date of IP [2]	P	P, C	P, C, A
≥ Injection date and ≤ Injection date + 27 days	-	C	C, A
> Injection date + 27 days	-	-	A

A = Post; C = Concomitant; P = Prior
[1] includes medications with completely missing end date
[2] includes medications with completely missing start date

9.4 *Appendix A Imputation Rules for Missing AE Dates*

Imputation rules for missing or partial AE start dates and end dates are defined below:

1. Missing or partial AE start date:
   - If only Day is missing, use the first day of the month, unless:
     - The AE end date is after the date of the injection or is missing AND the start month and year of the AE coincide with the start month and year of the injection. In this case, use the date and time of the injection, even if time is collected.
   - If Day and Month are both missing, use the first day of the year, unless:
     - The AE end date is after the date of the injection or is missing AND the start year of the AE coincides with the start year of the injection. In this case, use the date of the injection
   - If Day, Month and Year are all missing, the date will not be imputed. However, if the AE end date is prior to the date of the injection, then the AE will be considered a pre-treatment AE. Otherwise, the AE will be considered treatment-emergent.
2. Missing or partial AE end dates will not be imputed.

DocuSign Envelope ID: 264FC66D-43E3-471B-863F-16B5EDBC3D09

9.5 *Appendix E Severity Grading of Laboratory Abnormalities*

**Serum***	**Mild (Grade 1)**	**Moderate (Grade 2)**	**Severe (Grade 3)**	**Potentially Life Threatening (Grade 4)****
Sodium – Hyponatremia mEq/L	132 – 134	130 – 131	125 – 129	< 125
Sodium – Hypernatremia mEq/L	144 – 145	146 – 147	148 – 150	> 150
Potassium – Hyperkalemia mEq/L	5.1 – 5.2	5.3 – 5.4	5.5 – 5.6	> 5.6
Potassium – Hypokalemia mEq/L	3.5 – 3.6	3.3 – 3.4	3.1 – 3.2	< 3.1
Glucose – Hypoglycemia mg/dL	65 – 69	55 – 64	45 – 54	< 45
Glucose – Hyperglycemia<br>Fasting – mg/dL<br>Random – mg/dL	100 – 110<br>110 – 125	111 – 125<br>126 – 200	>125<br>>200	Insulin requirements or hyperosmolar coma
Blood Urea Nitrogen (BUN) mg/dL	23 – 26	27 – 31	> 31	Requires dialysis
Creatinine – mg/dL	1.5 – 1.7	1.8 – 2.0	2.1 – 2.5	> 2.5 or requires dialysis
Calcium – hypocalcemia mg/dL	8.0 – 8.4	7.5 – 7.9	7.0 – 7.4	< 7.0
Calcium – hypercalcemia mg/dL	10.5 – 11.0	11.1 – 11.5	11.6 – 12.0	> 12.0
Magnesium – hypomagnesemia mg/dL	1.3 – 1.5	1.1 – 1.2	0.9 – 1.0	< 0.9
Phosphorous – hypophosphatemia mg/dL	2.3 – 2.5	2.0 – 2.2	1.6 – 1.9	< 1.6
CPK – mg/dL	1.25 – 1.5 x ULN***	1.6 – 3.0 x ULN	3.1 – 10 x ULN	> 10 x ULN
Albumin – Hypoalbuminemia g/dL	2.8 – 3.1	2.5 – 2.7	< 2.5	--
Total Protein – Hypoproteinemia g/dL	5.5 – 6.0	5.0 – 5.4	< 5.0	--
Alkaline phosphate – increase by factor	1.1 – 2.0 x ULN	2.1 – 3.0 x ULN	3.1 – 10 x ULN	> 10 x ULN
Liver Function Tests –ALT, AST increase by factor	1.1 – 2.5 x ULN	2.6 – 5.0 x ULN	5.1 – 10 x ULN	> 10 x ULN
Bilirubin – when accompanied by any increase in Liver Function Test increase by factor	1.1 – 1.25 x ULN	1.26 – 1.5 x ULN	1.51 – 1.75 x ULN	> 1.75 x ULN
Bilirubin – when Liver Function Test is normal; increase by factor	1.1 – 1.5 x ULN	1.6 – 2.0 x ULN	2.0 – 3.0 x ULN	> 3.0 x ULN
Cholesterol	201 – 210	211 – 225	> 226	---
Pancreatic enzymes – amylase, lipase	1.1 – 1.5 x ULN	1.6 – 2.0 x ULN	2.1 – 5.0 x ULN	> 5.0 x ULN

DocuSign Envelope ID: 264FC66D-43E3-471B-863F-16B5EDBC3D09

* The laboratory values provided in the tables serve as guidelines and are dependent upon institutional normal parameters. Institutional normal reference ranges should be provided to demonstrate that they are appropriate.
** The clinical signs or symptoms associated with laboratory abnormalities might result in characterization of the laboratory abnormalities as Potentially Life Threatening (Grade 4). For example. a low sodium value that falls within a Grade 3 parameter (125-129 mE/L) should be recorded as a Grade 4 hyponatremia event if the participant had a new seizure associated with the low sodium value.
***ULN" is the upper limit of the normal range.

**Hematology ***	**Mild (Grade 1)**	**Moderate (Grade 2)**	**Severe (Grade 3)**	**Potentially Life Threatening (Grade 4)**
Hemoglobin (Female) - mg/dL	11.0 – 12.0	9.5 – 10.9	8.0 – 9.4	< 8.0
Hemoglobin (Female) change from baseline value - mg/dL	Any decrease – 1.5	1.6 – 2.0	2.1 – 5.0	> 5.0
Hemoglobin (Male) - mg/dL	12.5 – 13.5	10.5 – 12.4	8.5 – 10.4	< 8.5
Hemoglobin (Male) change from baseline value – mg/dL	Any decrease – 1.5	1.6 – 2.0	2.1 – 5.0	> 5.0
WBC Increase - cell/mm^3	10,800 – 15,000	15,001 – 20,000	20,001 – 25, 000	> 25,000
WBC Decrease - cell/mm^3	2,500 – 3,500	1,500 – 2,499	1,000 – 1,499	< 1,000
Lymphocytes Decrease - cell/mm^3	750 – 1,000	500 – 749	250 – 499	< 250
Neutrophils Decrease - cell/mm^3	1,500 – 2,000	1,000 – 1,499	500 – 999	< 500
Eosinophils - cell/mm^3	650 – 1500	1501 - 5000	> 5000	Hypereosinophilic
Platelets Decreased - cell/mm^3	125,000 – 140,000	100,000 – 124,000	25,000 – 99,000	< 25,000
PT – increase by factor (prothrombin time)	1.0 – 1.10 x ULN**	1.11 – 1.20 x ULN	1.21 – 1.25 x ULN	> 1.25 ULN
PTT – increase by factor (partial thromboplastin time)	1.0 – 1.2 x ULN	1.21 – 1.4 x ULN	1.41 – 1.5 x ULN	> 1.5 x ULN
Fibrinogen increase - mg/dL	400 – 500	501 – 600	> 600	--
Fibrinogen decrease - mg/dL	150 – 200	125 – 149	100 – 124	< 100 or associated with gross bleeding or disseminated intravascular coagulation (DIC)

* The laboratory values provided in the tables serve as guidelines and are dependent upon institutional normal parameters. Institutional normal reference ranges should be provided to demonstrate that they are appropriate.
** "ULN" is the upper limit of the normal range.

9.6 *Appendix F Severity Grading of Vital Sign Abnormalities*

**Vital Signs***	**Mild (Grade 1)**	**Moderate (Grade 2)**	**Severe (Grade 3)**	**Potentially Life Threatening (Grade 4)**
Fever (°C)**<br>(°F)**	38.0 – 38.4<br>100.4 – 101.1	38.5 – 38.9<br>101.2 – 102.0	39.0 – 40<br>102.1 – 104	> 40<br>> 104
Tachycardia - beats per minute	101 – 115	116 – 130	> 130	ER visit or hospitalization for arrhythmia
Bradycardia - beats per minute***	50 – 54	45 – 49	< 45	ER visit or hospitalization for arrhythmia
Hypertension (systolic) - mm Hg	141 – 150	151 – 155	> 155	ER visit or hospitalization for malignant hypertension
Hypertension (diastolic) - mm Hg	91 – 95	96 – 100	> 100	ER visit or hospitalization for malignant hypertension
Hypotension (systolic) – mm Hg	85 – 89	80 – 84	< 80	ER visit or hospitalization for hypotensive shock
Respiratory Rate – breaths per minute	17 – 20	21 – 25	> 25	Intubation

* Participant should be at rest for all vital sign measurements.
** Oral temperature; no recent hot or cold beverages or smoking.
*** When resting heart rate is between 60 – 100 beats per minute. Use clinical judgement when characterizing bradycardia among some healthy participant populations, for example, conditioned athletes.
