# Supplementary material for: Immunogenicity and safety of mRNA-based seasonal influenza vaccines encoding hemagglutinin and neuraminidase
Source: Nat Commun. 2025 Jul 1;16:5933. doi: 10.1038/s41467-025-60938-4 (PMC12216828; doi:10.1038/s41467-025-60938-4)
Supplement: Supplementary file 3 — Reporting Summary [file 41467_2025_60938_MOESM3_ESM.pdf]

nature portfolio

Corresponding author(s): Raffael Nachbagauer

Last updated by author(s): May 15, 2025

Reporting Summary

Nature Portfolio wishes to improve the reproducibility of the work that we publish. This form provides structure for consistency and transparency in reporting. For further information on Nature Portfolio policies, see our Editorial Policies and the Editorial Policy Checklist.

Statistics

For all statistical analyses, confirm that the following items are present in the figure legend, table legend, main text, or Methods section.

n/a	Confirmed	
☐	☒	The exact sample size (*n*) for each experimental group/condition, given as a discrete number and unit of measurement
☐	☒	A statement on whether measurements were taken from distinct samples or whether the same sample was measured repeatedly
☐	☒	The statistical test(s) used AND whether they are one- or two-sided<br>*Only common tests should be described solely by name; describe more complex techniques in the Methods section.*
☒	☐	A description of all covariates tested
☒	☐	A description of any assumptions or corrections, such as tests of normality and adjustment for multiple comparisons
☐	☒	A full description of the statistical parameters including central tendency (e.g. means) or other basic estimates (e.g. regression coefficient) AND variation (e.g. standard deviation) or associated estimates of uncertainty (e.g. confidence intervals)
☒	☐	For null hypothesis testing, the test statistic (e.g. *F*, *t*, *r*) with confidence intervals, effect sizes, degrees of freedom and *P* value noted<br>*Give P values as exact values whenever suitable.*
☒	☐	For Bayesian analysis, information on the choice of priors and Markov chain Monte Carlo settings
☒	☐	For hierarchical and complex designs, identification of the appropriate level for tests and full reporting of outcomes
☒	☐	Estimates of effect sizes (e.g. Cohen's *d*, Pearson's *r*), indicating how they were calculated

Our web collection on statistics for biologists contains articles on many of the points above.

Software and code

Policy information about availability of computer code

Data collection	Statistical analyses were performed using SAS version 9.4.
Data analysis	Statistical analyses were performed using SAS version 9.4.

For manuscripts utilizing custom algorithms or software that are central to the research but not yet described in published literature, software must be made available to editors and reviewers. We strongly encourage code deposition in a community repository (e.g. GitHub). See the Nature Portfolio guidelines for submitting code & software for further information.

Data

Policy information about availability of data

All manuscripts must include a data availability statement. This statement should provide the following information, where applicable:

- Accession codes, unique identifiers, or web links for publicly available datasets
- A description of any restrictions on data availability
- For clinical datasets or third party data, please ensure that the statement adheres to our policy

Access to participant-level data presented in this article and supporting clinical documents with external researchers who provide methodologically sound scientific proposals will be available upon reasonable request for products or indications that have been approved by regulators in the relevant markets and subject to review from 24 months after study completion. Such requests can be made to Moderna Inc., 325 Binney St, Cambridge, MA 02142 <<data_sharing@modernatx.com>>. A materials transfer and/or data access agreement with the sponsor will be required for accessing shared data. All other relevant data are presented in the paper. The protocol is available online at ClinicalTrials.gov: NCT05333289.

Research involving human participants, their data, or biological material

Policy information about studies with human participants or human data. See also policy information about sex, gender (identity/presentation), and sexual orientation and race, ethnicity and racism.

Reporting on sex and gender

The research findings presented within this manuscript do not apply to only one sex or gender. As the study design of this phase 1/2 trial did not allow for sex and gender-based analysis and this analysis was not prespecified, disaggregated findings for sex and gender were not included.

Reporting on race, ethnicity, or other socially relevant groupings

Medically stable adults aged 18-75 years were enrolled. As the study design of this phase 1/2 trial did not allow for sex and gender-based analysis and this analysis was not prespecified, disaggregated findings by race, ethnicity, or other socially relevant groupings are not included.

Population characteristics

A total of 572 participants were randomly assigned (1:1:1:1:1:1:1:1) to eight vaccine groups: mRNA-1020 at three dose levels (50, 100, or 150 µg), mRNA-1030 at three dose levels (25, 50, or 100 µg), an mRNA-based HA comparator (mRNA-1010 50 µg), or a licensed quadrivalent recombinant vaccine. Random allocation was stratified by age (18-49 vs. 50-75 years) to ensure balance of the two age groups within each vaccine group. Across all vaccine groups, most participants were female (55.0%) , White (80.5%) and non-Hispanic/Latino (91.0%). In the full analysis set, 13% of participants (73/565) were Black, which is reflective of the US population (12% Black). Median age of participants across all vaccine groups was 49.0 years. The majority of participants received the previous season's influenza vaccine.

Recruitment

Eligible participants were medically stable adults aged 18-75 years with a body mass index of 18-35 kg/m2. The trial was conducted at 15 sites in the United States and only included participants from the United States.

Ethics oversight

The study was conducted in accordance with the protocol, applicable laws, and regulatory requirements, as well as International Council for Harmonisation Good Clinical Practice guidelines, and the consensus ethical principles derived from international guidelines, including the Declaration of Helsinki and Council for International Organizations of Medical Sciences International Ethical Guidelines. The protocol was approved by the central institutional review board (Advarra, Inc. Columbia, MD) prior to study initiation, and written informed consent was obtained from all participants before enrollment. Participants were compensated for the study. Participants that completed all required study activities received a minimum of $720 plus travel reimbursement to maximum $1304. Compensation of minimum $65 plus travel reimbursement to maximum $160 was provided for any unscheduled safety visit.

Note that full information on the approval of the study protocol must also be provided in the manuscript.

Field-specific reporting

Please select the one below that is the best fit for your research. If you are not sure, read the appropriate sections before making your selection.

- [x] Life sciences
- [ ] Behavioural & social sciences
- [ ] Ecological, evolutionary & environmental sciences

For a reference copy of the document with all sections, see nature.com/documents/nr-reporting-summary-flat.pdf

Life sciences study design

All studies must disclose on these points even when the disclosure is negative.

Sample size

There is no hypothesis testing in this phase 1/2 study. As such, the sample size for this study was not driven by statistical assumptions for formal hypothesis testing. A total of approximately 560 participants, with 70 participants randomly assigned into each vaccine group, was planned and considered to be sufficient to provide descriptive safety and immunogenicity of different dose levels of mRNA-1020 or mRNA-1030.

In this phase 1/2 trial, 572 healthy adults were randomly assigned to receive different doses of mRNA-1020 (50, 100, or 150 µg), mRNA-1030 (25, 50, or 100 µg), mRNA-1010 (50 µg), or licensed (recombinant) active comparator (Flublok, Sanofi Pasteur Inc., Bridgewater, NJ, USA).

In all, 565 participants received a vaccination and were included in the full analysis set (mRNA-1020 [n = 208]; mRNA-1030 [n = 215],

	mRNA-1010 [n = 71], and the recombinant vaccine [n = 71]).
Data exclusions	No data were excluded from this analysis.
Replication	As this was a clinical trial, no replication was performed. However, we have included sufficient details in the Methods section of the manuscript, as well as full details in the Study Protocol, to allow for replication of this study.
Randomization	The sponsor's biostatistics department or designee generated the randomized allocation schedule for vaccine group assignment using interactive response technology. Vaccine dose preparation and administration were performed by unblinded personnel who had no other role in the conduct of the trial. Random allocation was stratified by age (18-49 vs. 50-75 years) to ensure balance of the two age groups within each vaccine group. Participants were randomized in a 1:1:1:1:1:1:1:1 ratio to receive either mRNA-1020 50 µg, mRNA-1020 100 µg, mRNA-1020 150 µg, mRNA-1030 25 µg, mRNA-1030 50 µg, mRNA-1030 100 µg, mRNA-1010 50 µg, or recombinant active comparator (Flublok, Sanofi Pasteur Inc., Bridgewater, NJ, USA) with approximately 70 medically-stable adult participants randomly assigned to each vaccination group.
Blinding	Vaccine assignment was observer blinded due to differing appearance of the study vaccines. Dose preparation, administration, and accountability of study vaccination were performed by designated unblinded clinic staff who who had no other role in the conduct of the trial. The unblinded clinic staff prepared the study vaccination out of view of the participant and the blinded clinic staff. All laboratory personnel in charge of immunogenicity testing were blinded to the vaccine assignment of samples tested throughout the course of the study. Neither the participant, nor the investigator, nor clinic staff responsible for study assessments/safety had access to the vaccine assignment during the conduct of the study. Except in the case of medical necessity, a participant's vaccine was not to be unblinded without the approval of the sponsor. The investigator, clinic staff, study participants, site monitors, and sponsor personnel (or its designees) were blinded to the study vaccine administered until the database was locked and unblinded for the final analysis. At the interim analysis, pre-identified sponsor team members and selected contract research organization team members were unblinded to conduct the analyses.

Reporting for specific materials, systems and methods

We require information from authors about some types of materials, experimental systems and methods used in many studies. Here, indicate whether each material, system or method listed is relevant to your study. If you are not sure if a list item applies to your research, read the appropriate section before selecting a response.

Materials & experimental systems

n/a	Involved in the study	
☒	☐	Antibodies
☒	☐	Eukaryotic cell lines
☒	☐	Palaeontology and archaeology
☒	☐	Animals and other organisms
☐	☒	Clinical data
☒	☐	Dual use research of concern
☒	☐	Plants

Methods

n/a	Involved in the study	
☒	☐	ChIP-seq
☒	☐	Flow cytometry
☒	☐	MRI-based neuroimaging

Clinical data

Policy information about clinical studies
All manuscripts should comply with the ICMJEguidelines for publication of clinical research and a completedCONSORT checklist must be included with all submissions.

Clinical trial registration	NCT05333289
Study protocol	The full protocol is provided as a supplementary file.
Data collection	This phase 1/2, randomized, observer-blind trial conducted at 15 US sites during the Northern Hemisphere Spring evaluated the safety, reactogenicity, and immunogenicity of mRNA-1020 and mRNA-1030 candidate seasonal influenza vaccines in healthy adults (NCT05333289). Participants were recruited into this study from March 31 to May 10, 2022. Study vaccine was administered on study Day 1, and safety follow-up continued through Day 181.
Outcomes	The primary objectives and secondary objectives of this study were to evaluate the safety, reactogenicity, and humoral immunogenicity of mRNA-1020, mRNA-1030, and mRNA-1010 against vaccine-matched influenza A and B strains.<br>The primary safety/reactogenicity end points were frequency and grade of each solicited local and systemic adverse reaction within 7 days after vaccination; frequency and severity of any unsolicited adverse events within 28 days after vaccination; and the frequency of any serious adverse event, adverse event of special interest, medically attended adverse event, or adverse event leading to withdrawal from study (Day 1 through to the end of study [Day 181]). The primary immunogenicity end points included geometric mean titers of anti-HA and anti-NA antibodies at baseline (Day 1) and Day 29; geometric mean fold rise of anti-HA and anti-NA antibodies at Day 29 versus baseline; percentage of participants with seroconversion at Day 29, as measured by the HAI assay; and percentage of participants with ≥2-fold, ≥3-fold, and ≥4-fold rise in titers at Day 29 as measured by NAI assay. Secondary immunogenicity end points included geometric mean titers and geometric mean fold rise (versus baseline) of anti-HA or anti-NA antibodies at all evaluable time points.

Plants

Seed stocks

Report on the source of all seed stocks or other plant material used. If applicable, state the seed stock centre and catalogue number. If plant specimens were collected from the field, describe the collection location, date and sampling procedures.

Novel plant genotypes

Describe the methods by which all novel plant genotypes were produced. This includes those generated by transgenic approaches, gene editing, chemical/radiation-based mutagenesis and hybridization. For transgenic lines, describe the transformation method, the number of independent lines analyzed and the generation upon which experiments were performed. For gene-edited lines, describe the editor used, the endogenous sequence targeted for editing, the targeting guide RNA sequence (if applicable) and how the editor was applied.

Authentication

Describe any authentication procedures for each seed stock used or novel genotype generated. Describe any experiments used to assess the effect of a mutation and, where applicable, how potential secondary effects (e.g. second site T-DNA insertions, mosiacism, off-target gene editing) were examined.
